# Organocatalytic asymmetric Michael/acyl transfer reaction between α-nitroketones and 4-arylidenepyrrolidine-2,3-diones

**DOI:** 10.3762/bjoc.17.100

**Published:** 2021-06-14

**Authors:** Chandrakanta Parida, Subhas Chandra Pan

**Affiliations:** 1 Department of Chemistry, Indian Institute of Technology Guwahati, North Guwahati, Assam, 781039, India

**Keywords:** acyl transfer, enantioselectivity, Michael reaction, organocatalysis, pyrrolidine-2,3-dione

## Abstract

An organocatalytic asymmetric Michael/acyl transfer reaction between α-nitroketones and 4-arylidenepyrrolidine-2,3-diones is reported. A bifunctional thiourea catalyst was found to be effective for this reaction. With 10 mol % of the catalyst, good results were attained for a variety of 1,5-dihydro-2*H*-pyrrol-2-ones under mild reaction conditions.

## Introduction

The Michael reaction is a powerful reaction that has been so far applied for the formation of carbon–carbon and carbon–heteroatom bonds in organic synthesis [1–2]. After the renaissance of organocatalysis in the year 2000, this field has been applied tremendously for the development of catalytic asymmetric conjugate addition reactions [3–5]. In particular, the conjugate addition of nitroalkanes and their derivatives to enones has drawn the attention of organic chemists as the corresponding products can be chemoselectively converted to a variety of useful structures [6]. Thus a variety of methods has been developed with a range of different catalysts [7–9]. One of the challenges is to employ highly substituted enones in the reaction. Indeed, additional substituents, especially at the α-position of enones/activated olefins, decreases the reactivity significantly because of unfavorable steric interactions. To overcome this problem, reactive Michael donors must be used to achieve a good conversion in the reaction. In recent years, α-nitroketones have emerged as active nucleophiles in Michael reactions and a range of substrates have been explored [10]. Also, α-nitroketones have been found to be a popular nucleophilic acyl transfer reagent. In 2011, three research groups namely Wang, Yan and Kwong independently revealed the organocatalytic asymmetric conjugate addition of α-nitroketones to β,γ-unsaturated α*-*keto esters with the concomitant acyl transfer reaction to the keto group [11–13]. Consequently, our group developed an organocatalytic asymmetric Michael–acyl transfer reaction of α-nitroketones with unsaturated pyrazolones, 2-hydroxycinnamaldehydes, γ/δ*-*hydroxyenones, *o*-quinone methides, etc. [14–18]. Other groups also contributed contemporarily [19–21].

**Scheme 1:**
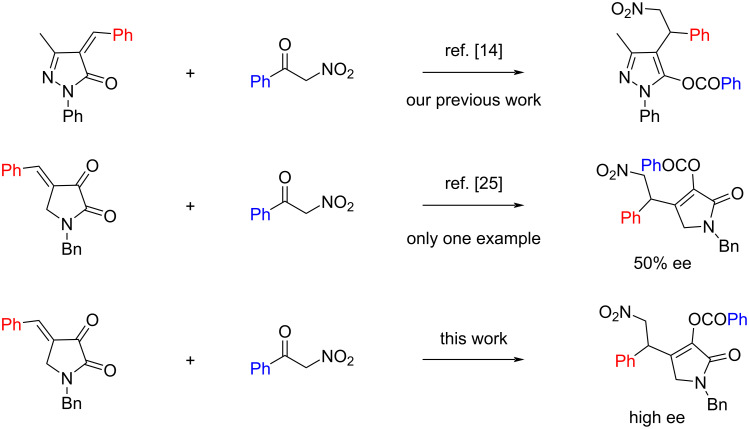
Reactions of α-nitroketones with unsaturated pyrazolone and with 4-benzylidenepyrrolidine-2,3-dione.

In recent years 4-arylidenepyrrolidine-2,3-diones have been explored mainly for the preparation of bicyclic dihydropyran derivatives through the catalytic inverse-electron-demand hetero-Diels–Alder reaction [22–24]. We postulated that 4-arylidenepyrrolidine-2,3-diones could also be suitable reaction partners of α-nitroketones. However, during the progress of our work, Bonne, Bugaut and co-workers have shown one example for the reaction of 2-nitroacetophenone with 4-benzylidenepyrrolidine-2,3-dione and only moderate enantioselectivity (50% ee) was achieved (Scheme 1) [25]. Herein, we report a better enantioselective version of the reaction between α-nitroketones and 4-arylidenepyrrolidine-2,3-diones (Scheme 1).

## Results and Discussion

Initially a model reaction was examined between *N*-benzyl-4-benzylidenepyrrolidine-2,3-dione (**1a**) and 2-nitro-1-phenylethanone (**2a**) in the presence of the quinine-derived bifunctional squaramide catalyst **I** in dichloromethane at room temperature (Table 1). Delightfully, after stirring for 12 hours, a product was isolated in 70% yield that was characterized as compound **3a** and was supposed to be formed through conjugate addition followed by benzoyl-transfer reaction. However, only 20% enantiomeric excess was achieved. Then, the *tert*-leucine-derived squaramide catalyst **II** was employed and here both yield and ee slightly improved. Next, we turned our attention to bifunctional thiourea catalysts [26–27] that proved to be fruitful. Thus, the quinine and cinchonidine-derived bifunctional thiourea catalysts **III** and **IV** were employed in the reaction and moderate enantiomeric excesses were achieved. The yield and enantioselectivity further improved when using the *tert*-leucine-derived thiourea catalyst **V**. Also, Takemoto’s catalyst **VI** [28] was suitable for the reaction though a moderate enantiomeric excess was detected. Finally, the best catalyst turned out to be the pyrrolidine-containing bifunctional thiourea catalyst **VII** and the desired product was isolated in 80% yield with 80% ee. Then, solvent optimization was carried out to obtain better enantioselectivities. A similar enantioselectivity was attained in α,α,α-trifluorotoluene and tetrahydrofuran as the solvent, whereas in chloroform a slightly improved enantioselectivity of 86% ee was observed. Finally, the best solvent was found to be 1,2-dichloroethane and the product **3a** was obtained in 82% yield with 90% ee.

**Table 1:** Catalyst screening and optimization of the reaction conditions.

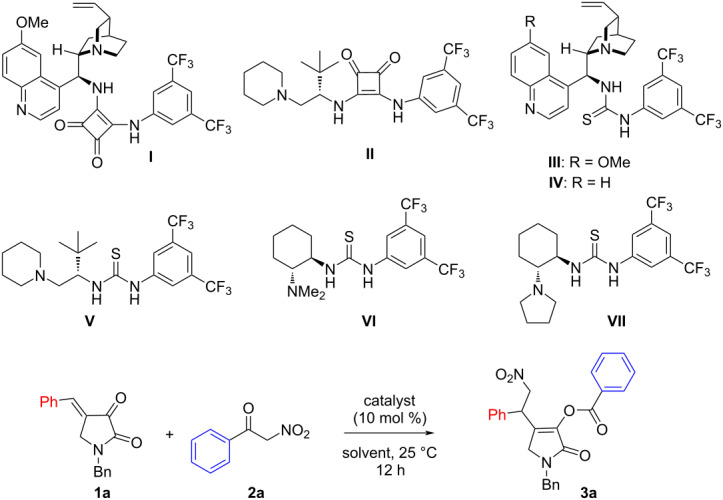				

entry^a^	catalyst	solvent	yield^b^	ee^c^

1	**I**	CH_2_Cl_2_	70	20
2	**II**	CH_2_Cl_2_	73	34
3	**III**	CH_2_Cl_2_	76	55
4	**IV**	CH_2_Cl_2_	78	52
5	**V**	CH_2_Cl_2_	80	74
6	**VI**	CH_2_Cl_2_	75	50
7	**VII**	CH_2_Cl_2_	80	80
8	**VII**	PhCF_3_	78	78
9	**VII**	THF	80	80
10	**VII**	CHCl_3_	80	86
11	**VII**	(CH_2_Cl)_2_	82	90

^a^Reactions were carried out with 0.1 mmol of **1a** and 0.1 mmol of **2a** in 0.6 mL solvent at 25 °C for 12 hours; ^b^isolated yield after silica gel column chromatography; ^c^determined by chiral HPLC.

After having identified the optimized conditions we ventured in the scope and generality of the reaction. Initially a variety of α-nitroketones **1** having different aryl substituents were tested (Table 2). In fact, different *ortho*-, *meta*-, and *para*-substitutions on the phenyl group were compatible with the reaction conditions and satisfactory results were obtained (Table 2, entries 2–11). For example, *p*-tolyl-containing nitroketone **2b** delivered the product **3b** in 80% yield with 88% ee (Table 2, entry 2). A similar enantioselectivity was obtained for product **3c** with a *p*-anisyl group (Table 2, entry 3). Interestingly, the enantioselectivity dropped slightly when replacing a *p*-methoxy substituent with a *p*-ethoxy group and product **3d** was isolated in 78% yield with 80% ee (Table 2, entry 4). Also, a biphenyl group was tolerated and a good result was achieved (Table 2, entry 5). Then, 4-fluoro and 4-bromo-containing nitroketones **2f** and **2g** were employed in the reaction and gratifyingly the same 90% ee were obtained for both products **3f** and **3g** (Table 2, entries 6 and 7). *meta*-Substitutions were also tolerated in the reaction although decreased enantioselectivities were detected for the products **3h** and **3i**, respectively (Table 2, entries 8 and 9). Then, *o*-methyl*- and o*-methoxyphenyl-substituted nitroketones **2j** and **2k** were employed in the reaction. Here also, the reactions progressed well to provide products **3j** and **3k** in moderate yields and enantioselectivities (Table 2, entries 10 and 11). The 2-naphthyl-substituted nitroketone **2l** also participated in the reaction to deliver **3l** in 80% ee (Table 2, entry 12). Moreover, the hydrocinnamyl group containing nitroketone **2m** also took part in the reaction and the corresponding product **3m** was isolated in 65% yield with 64% ee (Table 2, entry 13). Finally, nitroketone **2n** with a cyclohexyl group was engaged in the reaction and a moderate enantioselectivity was detected for product **3n** (Table 2, entry 14).

**Table 2:** Scope of α-nitroketones **2** in the reaction with **1a**.

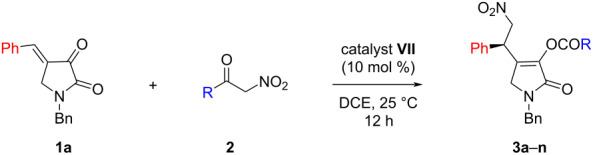				

entry^a^	R	**3**	yield^b^	ee^c^

1	Ph	**3a**	80	90
2	4-MeC_6_H_4_	**3b**	80	88
3	4-MeOC_6_H_4_	**3c**	82	88
4	4-EtOC_6_H_4_	**3d**	78	80
5	4-PhC_6_H_4_	**3e**	82	82
6	4-FC_6_H_4_	**3f**	79	90
7	4-BrC_6_H_4_	**3g**	78	90
8	3-MeC_6_H_4_	**3h**	70	72
9	3-MeOC_6_H_4_	**3i**	72	66
10	2-MeC_6_H_4_	**3j**	65	68
11	2-MeOC_6_H_4_	**3k**	68	70
12	2-naphthyl	**3l**	75	80
13	PhCH_2_CH_2_	**3m**	65	64
14	cyclohexyl	**3n**	70	72

^a^The reactions were carried out with 0.1 mmol of **1a** and 0.1 mmol of **2** in 0.6 mL 1,2-dichloroethane at 25 °C for 12 hours; ^b^isolated yield after silica gel column chromatography; ^c^determined by chiral HPLC.

In the next step, we investigated the scope of the reaction of substrate **2a** with a variety of pyrrolidine-2,3-diones **1** having different benzylidene substituents under the optimized conditions (Table 3). It turned out that a range of substitutions was tolerated and good results were attained. Initially, different *para*-substituted arylidene substrates were screened that smoothly afforded products **3o**–**s** (Table 3, entries 1–5). For example, the pyrrolidine-2,3-dione **1b** with a 4-methylbenzylidene-substituent provided the product **3o** in 83% yield and 72% ee (Table 3, entry 1). A similar enantioselectivity was obtained with the 4-*tert*-butylenzylidene-substituted pyrrolidine-2,3-dione **1c** (Table 3, entry 2). Then, different 4-halobenzylidene-substituted pyrrolidine-2,3-diones **1d–f** were employed in the reaction and mixed results were obtained. Although product **3q** having a 4-fluorophenyl-substitution was isolated in 80% yield and 84% ee, slightly decreased enantioselectivities were obtained for the corresponding 4-chloro- (**3r**, 70% ee) and 4-bromophenyl (**3s**, 76% ee) derivatives (Table 3, entries 3–5). These products could be particularly useful for further transformations via cross-coupling reactions. The *ortho*-fluoroarylidene-substituted pyrrolidine-2,3-dione **1g** also participated in the reaction to deliver product **3t** in 86% ee (Table 3, entry 6). 2,4-Disubstitution at the aromatic ring was also tolerated in the reaction and a moderate enantioselectivity was observed for the 2,4-difluorophenyl-substituted product **3u** (Table 3, entry 7). The 3,5-dimethoxybenzylidene-containing pyrrolidine-2,3-dione **1i** was prepared and also engaged in the reaction. Here also, a smooth conversion was detected and the product **3v** was isolated in 80% yield with 72% ee (Table 3, entry 8). Finally, pyrrolidine-2,3-dione **1j** containing a heteroaromatic group was also screened and an acceptable enantioselectivity for the 2-thienyl-substituted product **3w** was witnessed (Table 3, entry 9).

**Table 3:** Scope of pyrrolidine-2,3-diones **1** in the reaction with **2a**.

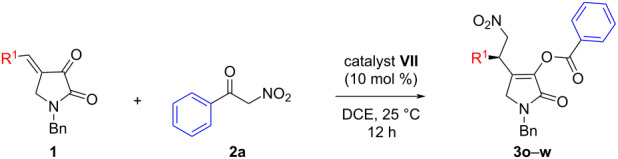					

entry^a^	R^1^	**1**	**3**	yield^b^	ee^c^

1	4-MeC_6_H_4_	**1b**	**3o**	83	72
2	4-*t-*BuC_6_H_4_	**1c**	**3p**	80	72
3	4-FC_6_H_4_	**1d**	**3q**	80	84
4	4-ClC_6_H_4_	**1e**	**3r**	79	70
5	4-BrC_6_H_4_	**1f**	**3s**	82	76
6	2-FC_6_H_4_	**1g**	**3t**	79	86
7	2,4-F_2_C_6_H_3_	**1h**	**3u**	78	72
8	3,5-(MeO)_2_C_6_H_3_	**1i**	**3v**	80	72
9	2-thienyl	**1j**	**3w**	81	82

^a^Reactions were carried out with 0.1 mmol of **1** and 0.1 mmol of **2a** in 0.6 mL 1,2-dichloroethane at 25 °C for 12 hours; ^b^isolated yield after silica gel column chromatography; ^c^determined by chiral HPLC.

**Scheme 2:**
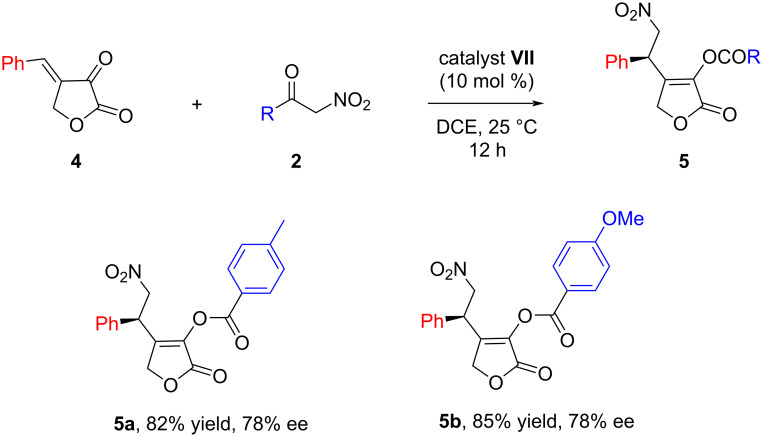
Reaction of 4-benzylidenedihydrofuran-2,3-dione (**4**) with α-nitroketones **2b**,**c**. Reaction conditions: furan **4** (0.1 mmol), α-nitroketone **2** (0.1 mmol), 10 mol % **VII** in 0.6 mL 1,2-dichloroethane were reacted at 25 °C for 12 hours. Yields correspond to isolated yields after silica gel column chromatography and ees were determined by chiral HPLC.

To further expand the scope of the reaction, 4-benzylidenedihydrofuran-2,3-dione (**4**) was prepared and reacted with nitroketones **2b** and **2c**, respectively. To our delight, the reactions proceeded smoothly at room temperature providing the desired products **5a** and **5b** in good yields and enantioselectivities (Scheme 2).

## Conclusion

In summary, in this paper we reported an organocatalytic asymmetric Michael/acyl transfer reaction between α-nitroketones and 4-arylidenepyrrolidine-2,3-diones/4-benzylidenedihydrofuran-2,3-dione. The products were obtained in good yields with moderate to high enantioselectivities. An easily available bifunctional thiourea catalyst was employed in the methodology.

## Supporting Information

File 1 Experimental part.
