# Supplementary material for: Organocatalytic asymmetric Michael/acyl transfer reaction between α-nitroketones and 4-arylidenepyrrolidine-2,3-diones
Source: Beilstein J Org Chem. 2021 Jun 14;17:1447–52. doi: 10.3762/bjoc.17.100 (PMC8218544; doi:10.3762/bjoc.17.100)
Supplement: File 1 — Experimental part. [file Beilstein_J_Org_Chem-17-1447-s001.pdf]

BEILSTEIN JOURNAL OF ORGANIC CHEMISTRY

Supporting Information

for

Organocatalytic asymmetric Michael/acyl transfer reaction between α-nitroketones and 4-arylidenepyrrolidine-2,3-diones

Chandrakanta Parida and Subhas Chandra Pan

Beilstein J. Org. Chem. **2021,** *17,* 1447–1452. doi:10.3762/bjoc.17.100

Experimental part

License and Terms: This is a supporting information file under the terms of the Creative Commons Attribution License (https://creativecommons.org/licenses/by/4.0). Please note that the reuse, redistribution and reproduction in particular requires that the author(s) and source are credited and that individual graphics may be subject to special legal provisions.
The license is subject to the *Beilstein Journal of Organic Chemistry* terms and conditions: (https://www.beilstein-journals.org/bjoc/terms)

Table of contents

1. General information S2

2. General procedure for the synthesis of 4-arylidene-pyrrolidine-2,3-diones.......................S2

3. General procedure for the synthesis of α-nitroketone...S2

4. General procedure for the synthesis of compound **3a and 5a** S2

5. Characterization of the products S3–S14

6. NMR spectra of the products... S15–S39

7. HPLC chromatogram of the products S40–S64

8. References..S64

1. General information

Chemicals and solvents were purchased from commercial suppliers and were used as received. ^{1}H NMR spectra were recorded at 400 MHz, 500MHz, and 600 MHz; ^{13}C NMR spectra were recorded at 100 MHz and 150 MHz. Chemical shifts are reported in parts per million (ppm), and the residual solvent peak was used as an internal reference: proton (chloroform δ 7.260), carbon (chloroform δ 77.23). Multiplicity was indicated as follows: s (singlet), d (doublet), t (triplet), q (quartet), m (multiplet), dd (doublet of doublet), brs (broad singlet). Coupling constants were reported in hertz (Hz). ESI was used for HRMS spectra. Enantiomeric ratios were determined by HPLC analysis performed on Chiral columns using a Daicel Chiralpak IA, IF, ID and AD-H column. For visualizing the products UV light and I_2 were used. Silica gel (60–120 mesh) was used for the column chromatography. Reactions were monitored by TLC on silica gel 60 F254 (0.25 mm).

2. Procedure for the synthesis of 4-arylidene-pyrrolidine-2,3-diones[1]

4-Arylidene-pyrrolidine-2,3-diones were prepared according to reported procedures.

3. Procedure for the synthesis of α-nitroketone[2]

α-Nitroketone was prepared according to reported procedures.

4. General procedure for the synthesis of compounds 3 and 5

In an oven-dried round-bottomed flask, compound **1a** (27.7 mg, 0.1 mmol), **2a** (16.5 mg, 0.1 mmol) and 10 mol % of catalyst **VII** were placed. Then, 0.6 mL 1,2-DCE was added and the reaction mixture was stirred at room temperature for 12 h. The progress of the reaction was monitored by TLC. After completion, the mixture was concentrated and directly purified by column chromatography on silica gel eluting with hexane/ethyl acetate (10–12%) to afford the desired product **3a**–**w**.

In an oven-dried round-bottomed flask, compound **4a** (19 mg, 0.1 mmol), **2a** (16.5 mg, 0.1 mmol) and 10 mol % of catalyst **VII** were placed. Then, 0.6 mL 1,2-DCE was added and the reaction mixture was stirred at room temperature for 12 h. The progress of the reaction was monitored by TLC. After completion the mixture was concentrated and directly purified by column chromatography on silica gel eluting with DCM to afford the desired product **5a**–**c**.

5. Characterization of the products

(*R*)-1-Benzyl-4-(2-nitro-1-phenylethyl)-2-oxo-2,5-dihydro-1*H*-pyrrol-3-yl benzoate (3a)

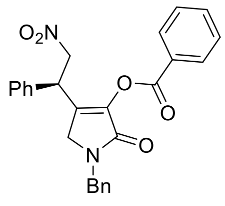

3a

Yellow sticky, 80% (35mg) yield. **^{1}H NMR (400 MHz, $CDCl_3$)** δ 8.21 – 8.15 (m, 2H), 7.70 – 7.64 (m, 1H), 7.53 (t, J = 7.8 Hz, 2H), 7.37 – 7.27 (m, 6H), 7.22 (ddd, J = 7.8, 3.7, 1.6 Hz, 4H), 4.99 (dd, J = 13.5, 8.0 Hz, 1H), 4.78 (dd, J = 13.5, 8.0 Hz, 1H), 4.66 (d, J = 15.1 Hz, 1H), 4.60 (t, J = 7.9 Hz, 1H), 4.52 (d, J = 15.1 Hz, 1H), 3.70 (s, 2H).**^{13}C NMR (100 MHz, $CDCl_3$)** δ 164.44, 163.89, 140.87, 136.49, 136.16, 135.91, 134.56, 130.90, 129.78, 129.12, 128.97, 128.88, 128.26, 128.07, 127.99, 127.86, 77.00, 48.96, 46.99, 42.76.

ESI HRMS: calcd. For $C_{26}H_{22}N_2O_5$ $[M+H]^+$ 443.1601, found 443.1601.

HPLC Analysis: ee 90%, IF column, *n*-hexane/iPrOH 70:30, flow rate 1.0 mL/min, λ = 220 nm (t_{major}= 20.9 min, t_{minor} = 23.3 min).

From literature study, the optical rotation of the compound 3a is $[\alpha]_D^{20}$ = 30.69 (*c* 1.03, $CHCl_3$, 25.1 °C).[3]

This sample was measured on an Autopol I, Serial #35386
Manufactured by Rudolph Research Analytical, Hackettstown, NJ, USA.

Measurement Date : Friday, 12-MAR-2021

Set Temperature : OFF

Time Delay : Disabled

n	Average	Std.Dev.	% RSD	Maximum	Minimum
5	-35.00	3.70	-10.57	-31.67	-41.11

S.No	Sample ID	Time	Result	Scale	OR °Arc	WLG.nm	Lg.mm	Temp.
1	mm acyl1	02:30 PM	-41.11	SR	-0.074	589	100.00	27.5
2	mm acyl1	02:30 PM	-33.89	SR	-0.061	589	100.00	27.5
3	mm acyl1	02:30 PM	-32.78	SR	-0.059	589	100.00	27.5
4	mm acyl1	02:30 PM	-31.67	SR	-0.057	589	100.00	27.5
5	mm acyl1	02:31 PM	-35.56	SR	-0.064	589	100.00	27.5

$[\alpha]_D^{20}$ = −35.00 (c 1.03, $CHCl_3$, 27.5 °C)

(*R*)-1-Benzyl-4-(2-nitro-1-phenylethyl)-2-oxo-2,5-dihydro-1*H*-pyrrol-3-yl 4-methylbenzoate (3b).

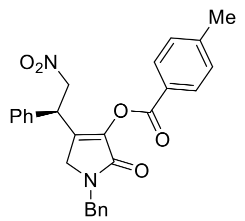

3b

Light orange sticky, 80% (36mg) yield. **^{1}H NMR (400 MHz, $CDCl_3$)** δ 8.07 (d, J = 8.2 Hz, 2H), 7.41 – 7.26 (m, 8H), 7.26 – 7.15 (m, 4H), 4.98 (dd, J = 13.5, 7.9 Hz, 1H), 4.77 (dd, J = 13.5, 8.0 Hz, 1H), 4.67 (d, J = 15.1 Hz, 1H), 4.57 (t, J = 8.0 Hz, 1H), 4.51 (d, J = 15.1 Hz, 1H), 3.69 (d, J = 3.0 Hz, 2H), 2.46 (s, 3H). **^{13}C NMR (100 MHz, $CDCl_3$)** δ 164.51, 163.95, 145.62, 140.87, 136.51, 136.00, 135.95, 130.98,

129.78, 129.70, 129.11, 128.88, 128.26, 128.06, 127.88, 125.16, 76.91, 48.96, 46.95, 42.81, 22.08.

ESI HRMS: calcd. For $C_{27}H_{24}N_2O_5$ $[M+H]^+$ 457.1758, found 457.1758.

HPLC Analysis: ee 88%, IF column, *n*-hexane/iPrOH 70:30, flow rate 1.0 mL/min, λ = 220 nm (t_{major}= 24.6 min, t_{minor} = 26.5 min).

(*R*)-1-Benzyl-4-(2-nitro-1-phenylethyl)-2-oxo-2,5-dihydro-1*H*-pyrrol-3-yl 4-methoxybenzoate (3c).

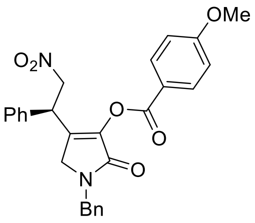

3c

Colourless sticky solid, 82% (38mg) yield. **^{1}H NMR (400 MHz, CDCl$_3$)** δ 8.13 (d, J = 8.9 Hz, 2H), 7.42 – 7.27 (m, 6H), 7.22 (dt, J = 10.5, 4.1 Hz, 4H), 7.04 – 6.96 (m, 2H), 4.99 (dd, J = 13.5, 7.9 Hz, 1H), 4.77 (dd, J = 13.5, 8.0 Hz, 1H), 4.67 (d, J = 15.0 Hz, 1H), 4.57 (t, J = 8.0 Hz, 1H), 4.50 (d, J = 15.1 Hz, 1H), 3.91 (s, 3H), 3.68 (d, J = 3.1 Hz, 2H). **^{13}C NMR (100 MHz, CDCl$_3$)** δ 164.74, 164.61, 163.59, 140.88, 136.51, 135.97, 135.95, 133.15, 129.75, 129.09, 128.85, 128.25, 128.04, 127.89, 120.14, 114.28, 76.91, 55.79, 48.95, 46.93, 42.80.

ESI HRMS: calcd. For $C_{27}H_{24}N_2O_6$ $[M+H]^+$ 473.1707, found 473.1707.

HPLC Analysis: ee 80%, Chiralpak IA column, *n*-hexane/iPrOH 80:20, flow rate 1.0 mL/min, λ = 254 nm (t_{major} = 43.5 min, t_{minor} = 47.4 min).

(*R*)-1-Benzyl-4-(2-nitro-1-phenylethyl)-2-oxo-2,5-dihydro-1H-pyrrol-3-yl 4-ethoxybenzoate (3d).

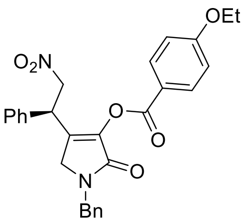

3d

Pale yellow semi solid, 78% (38mg) yield. **^{1}H NMR (400 MHz, CDCl$_3$)** δ 8.12 (d, J = 8.9 Hz, 2H), 7.38 – 7.26 (m, 6H), 7.25 – 7.18 (m, 4H), 6.98 (d, J = 8.9 Hz, 2H), 4.96 (s, 1H), 4.78 (s, 1H), 4.65 (s, 1H), 4.57 (s, 1H), 4.52 (s, 1H), 4.13 (d, J = 7.0 Hz, 2H), 3.68 (d, J = 3.4 Hz, 2H), 1.46 (s, 3H).**^{13}C NMR (100 MHz, CDCl$_3$)** δ 164.64, 164.18, 163.64, 140.90, 136.52, 135.99, 135.93, 133.15, 129.76, 129.09, 128.85, 128.25, 128.04, 127.90, 119.88, 114.70, 76.91, 64.11, 48.95, 46.94, 42.82, 14.85.

ESI HRMS: calcd. For $C_{28}H_{26}N_2O_6$ $[M+H]^+$ 487.1864, found 487.1863.

HPLC Analysis: ee 80%, Chiralpak IA column, *n*-hexane/iPrOH 80:20, flow rate 1.0 mL/min, λ = 254 nm (t_{major} = 38.6 min, t_{minor} = 44.5 min).

(*R*)-1-Benzyl-4-(2-nitro-1-phenylethyl)-2-oxo-2,5-dihydro-1*H*-pyrrol-3-yl [1,1'-biphenyl]-4-carboxylate (3e)

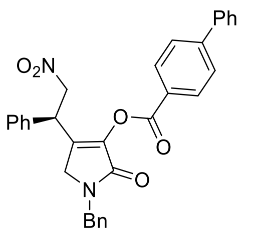

3e

Light yellow sticky solid, 82% (42mg) yield. **^{1}H NMR (400 MHz, CDCl$_3$)** δ 8.28 – 8.23 (m, 2H), 7.78 – 7.73 (m, 2H), 7.67 (dd, J = 5.2, 3.3 Hz, 2H), 7.54 – 7.47 (m, 2H), 7.46 – 7.41 (m, 1H), 7.37 – 7.28 (m, 6H), 7.23 (dt, J = 10.8, 4.1 Hz, 4H), 5.01 (dd, J = 13.5, 8.0 Hz, 1H), 4.79 (dd, J = 13.5, 7.9 Hz, 1H), 4.68 (d, J = 15.1 Hz, 1H), 4.61 (t, J = 8.0 Hz, 1H), 4.53 (d, J = 15.1 Hz, 1H), 3.71 (d, J = 2.2 Hz, 2H).**^{13}C NMR (100 MHz, CDCl$_3$)** δ 164.51, 163.82, 147.36, 140.88, 139.94, 136.48, 136.11, 135.90, 131.46, 129.81, 129.23, 129.13, 128.92, 128.68, 128.27, 128.09, 127.89, 127.64, 127.61, 126.58, 77.43, 48.98, 46.98, 42.80.

ESI HRMS: calcd. For $C_{32}H_{26}N_2O_5$ $[M+H]^+$ 519.1914, found 519.1914.

HPLC Analysis: ee 82%, Chiralpak IA column, *n*-hexane/iPrOH 80:20, flow rate 1.0 mL/min, λ = 254 nm (t_{major} = 45.7 min, t_{minor} = 51.5 min).

(*R*)-1-Benzyl-4-(2-nitro-1-phenylethyl)-2-oxo-2,5-dihydro-1H-pyrrol-3-yl 4-fluorobenzoate (3f)

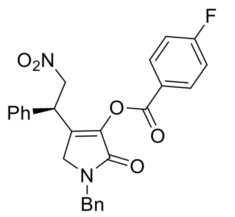

3f

Yellow semi solid, 79% (36mg) yield. **^{1}H NMR (400 MHz, CDCl$_3$)** δ 8.26 – 8.12 (m, 2H), 7.38 – 7.27 (m, 6H), 7.26 – 7.14 (m, 6H), 4.97 (dd, J = 13.5, 8.3 Hz, 1H), 4.76 (dd, J = 13.5, 7.6 Hz, 1H), 4.67 (d, J = 15.0 Hz, 1H), 4.61 (d, J = 8.0 Hz, 1H), 4.52 (d, J = 15.1 Hz, 1H), 3.70 (s, 2H).**^{13}C NMR (100 MHz, CDCl$_3$)** 13C NMR (101 MHz, CDCl3) δ 168.12, 165.57, 164.34, 162.92, 140.73, 136.41, 136.20, 135.77, 133.69, 133.59, 129.82, 129.14, 128.94, 128.25, 128.11, 127.84, 124.22, 124.19, 116.40, 116.18, 77.43, 48.94, 46.98, 42.70.

ESI HRMS: calcd. For $C_{26}H_{21}FN_2O_5$ $[M+H]^+$ 461.1507, found 461.1506.

HPLC Analysis: ee 90%, Chiralpak IF column, *n*-hexane/iPrOH 80:20, flow rate 1.0 mL/min, λ = 220 nm (t_{major} = 39.3 min, t_{minor} = 42.7 min).

(*R*)-1-Benzyl-4-(2-nitro-1-phenylethyl)-2-oxo-2,5-dihydro-1*H*-pyrrol-3-yl 4-bromobenzoate (3g)

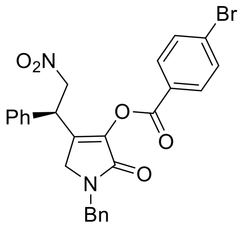

3g

Light yellow sticky solid, 78% (40mg) yield. **^{1}H NMR (400 MHz, $CDCl_3$)** δ 8.03 (d, J = 8.6 Hz, 2H), 7.68 (d, J = 8.6 Hz, 2H), 7.38 – 7.26 (m, 6H), 7.21 (d, J = 6.9 Hz, 4H), 4.96 (dd, J = 13.5, 8.4 Hz, 1H), 4.75 (dd, J = 13.5, 7.5 Hz, 1H), 4.66 (d, J = 15.0 Hz, 1H), 4.60 (t, J = 8.0 Hz, 1H), 4.52 (d, J = 15.1 Hz, 1H), 3.70 (s, 2H). **^{13}C NMR (100 MHz, $CDCl_3$)** δ 164.26, 163.23, 140.72, 136.41, 136.27, 135.75, 132.40, 132.30, 130.03, 129.83, 129.15, 128.96, 128.26, 128.12, 127.83, 126.88, 77.43, 48.95, 47.01, 42.69.

ESI HRMS: calcd. For $C_{26}H_{21}BrN_2O_5$ $[M+H]^+$ 521.0707, found 521.0708.

HPLC Analysis: ee 90%, Chiralpak IA column, *n*-hexane/iPrOH 80:20, flow rate 1.0 mL/min, λ = 254 nm (t_{major} = 43.6 min, t_{minor} = 47.9 min).

(*R*)-1-Benzyl-4-(2-nitro-1-phenylethyl)-2-oxo-2,5-dihydro-1*H*-pyrrol-3-yl 3-methylbenzoate (3h)

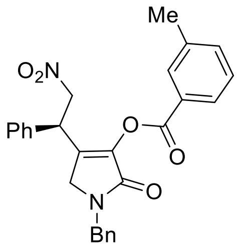

3h

Yellow sticky, 70% (32mg) yield. **^{1}H NMR (600 MHz, $CDCl_3$)** δ 8.01 – 7.96 (m, 2H), 7.48 (d, J = 7.4 Hz, 1H), 7.42 (t, J = 7.6 Hz, 1H), 7.36 – 7.27 (m, 6H), 7.24 – 7.19 (m, 4H), 4.99 (dd, J = 13.5, 7.9 Hz, 1H), 4.78 (dd, J = 13.5, 8.1 Hz, 1H), 4.67 (d, J = 15.1 Hz, 1H), 4.58 (t, J = 8.0 Hz, 1H), 4.51 (d, J = 15.1 Hz, 1H), 3.70 (d, J = 5.5 Hz, 2H), 2.45 (s, 3H). **^{13}C NMR (150 MHz, $CDCl_3$)** δ 164.45, 164.10, 140.79, 138.84, 136.46, 136.07, 135.87, 135.38, 131.41, 129.76, 129.09, 128.87, 128.85, 128.24, 128.05, 127.86, 127.78,76.98, 48.94, 46.93, 42.76, 21.49.

ESI HRMS: calcd. For $C_{27}H_{24}N_2O_5$ $[M+H]^+$ 457.1758, found 457.1757.

HPLC Analysis: ee 72%, Chiralpak IF column, *n*-hexane/iPrOH 70:30, flow rate 1.0 mL/min, λ = 220 nm (t_{major} = 21.0 min, t_{minor} = 23.3 min).

(*R*)-1-Benzyl-4-(2-nitro-1-phenylethyl)-2-oxo-2,5-dihydro-1*H*-pyrrol-3-yl 3-methoxybenzoate (3i)

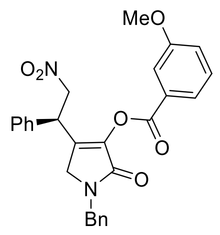

3i

Orange semi solid 72% (34mg) yield. **^{1}H NMR (600 MHz, CDCl$_3$)** δ 7.79 (dt, J = 7.6, 1.2 Hz, 1H), 7.68 (dd, J = 2.7, 1.6 Hz, 1H), 7.43 (t, J = 8.0 Hz, 1H), 7.36 – 7.27 (m, 6H), 7.22 (ddd, J = 6.8, 4.6, 2.5 Hz, 5H), 5.00 (dd, J = 13.6, 8.3 Hz, 1H), 4.76 (dd, J = 13.6, 7.7 Hz, 1H), 4.68 (d, J = 15.1 Hz, 1H), 4.58 (t, J = 8.0 Hz, 1H), 4.51 (d, J = 15.1 Hz, 1H), 3.89 (s, 3H), 3.70 (d, J = 4.8 Hz, 2H).**^{13}C NMR (150 MHz, CDCl$_3$)** δ 164.37, 163.79, 159.93, 140.79, 136.43, 136.07, 135.78, 129.98, 129.79, 129.10, 129.06, 128.90, 128.23, 128.06, 127.85, 123.36, 121.52, 114.82, 76.96, 55.76, 48.96, 46.93, 42.76.

ESI HRMS: calcd. For $C_{27}H_{24}N_2O_6$ $[M+H]^+$ 473.1707, found 473.1714.

HPLC Analysis: ee 66%, Chiralpak IA column, *n*-hexane/iPrOH 70:30, flow rate 1.0 mL/min, λ = 220 nm (t_{major} = 22.4 min, t_{minor} = 18.5 min).

(*R*)-1-Benzyl-4-(2-nitro-1-phenylethyl)-2-oxo-2,5-dihydro-1*H*-pyrrol-3-yl 2-methylbenzoate (3j)

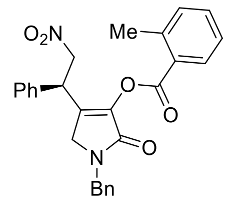

3j

Pale yellow semi solid, 65% (29mg) yield. **^{1}H NMR (400 MHz, CDCl$_3$)** δ 8.14 (dd, J = 8.2, 1.5 Hz, 1H), 7.51 (td, J = 7.5, 1.5 Hz, 1H), 7.41 – 7.26 (m, 8H), 7.22 (ddd, J = 7.7, 4.0, 1.6 Hz, 4H), 5.00 (dd, J = 13.4, 7.8 Hz, 1H), 4.79 (dd, J = 13.4, 8.1 Hz, 1H), 4.67 (d, J = 15.1 Hz, 1H), 4.59 (t, J = 7.9 Hz, 1H), 4.53 (d, J = 15.0 Hz, 1H), 3.70 (d, J = 2.0 Hz, 2H), 2.70 (s, 3H).**^{13}C NMR (100 MHz, CDCl$_3$)** δ 164.57, 164.15, 142.43, 140.91, 136.46, 136.00, 135.96, 133.71, 132.25, 131.94, 129.77, 129.12, 128.88, 128.27, 128.08, 127.85, 126.94, 126.31,77.43, 48.97, 46.98, 42.71, 22.17.

ESI HRMS: calcd. For $C_{27}H_{24}N_2O_5$ $[M+H]^+$ 457.1758, found 457.1757.

HPLC Analysis: ee 68%, Chiralpak ADH column, *n*-hexane/iPrOH 80:20, flow rate 1.0 mL/min, λ = 220 nm (t_{major} = 15.9 min, t_{minor} = 14.9 min).

(*R*)-1-Benzyl-4-(2-nitro-1-phenylethyl)-2-oxo-2,5-dihydro-1*H*-pyrrol-3-yl 2-methoxybenzoate (3k)

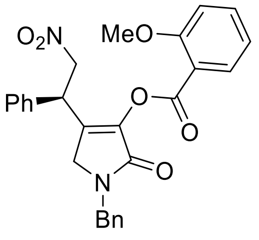

3j

Yellow sticky solid, 68% (32mg) yield. **^{1}H NMR (400 MHz, CDCl$_3$)** δ 8.04 (dd, J = 7.8, 1.8 Hz, 1H), 7.61 – 7.56 (m, 1H), 7.35 – 7.27 (m, 8H), 7.23 – 7.19 (m, 2H), 7.09 – 7.03 (m, 2H), 5.10 (dd, J = 13.5, 6.9 Hz, 1H), 4.94 (dd, J = 13.6, 9.0 Hz, 1H), 4.68 (d, J = 15.0 Hz, 1H), 4.56 – 4.47 (m, 2H), 3.96 (s, 3H), 3.68 (d, J = 10.5 Hz, 2H).**^{13}C NMR (100 MHz, CDCl$_3$)** δ 164.56, 163.34, 160.16, 136.57, 136.31, 135.98, 135.33, 133.27, 129.72, 129.09, 128.83, 128.24, 128.03, 128.00, 120.68, 117.58, 112.26, 77.43, 56.21, 49.10, 46.91, 42.98.

ESI HRMS: calcd. For $C_{27}H_{24}N_2O_6$ $[M+H]^+$ 473.1707, found 473.1711.

HPLC Analysis: ee 70%, Chiralpak ID column, *n*-hexane/iPrOH 70:30, flow rate 1.0 mL/min, λ = 220 nm (t_{major} = 59.0 min, t_{minor} = 72.9 min).

(*R*)-1-Benzyl-4-(2-nitro-1-phenylethyl)-2-oxo-2,5-dihydro-1*H*-pyrrol-3-yl 2-naphthoate (3l)

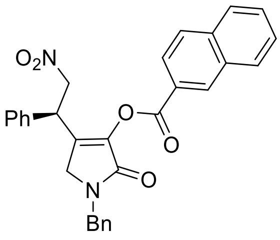

3l

White sticky solid, 75% (37mg) yield. **^{1}H NMR (600 MHz, CDCl$_3$)** δ 8.78 (s, 1H), 8.15 (dd, J = 8.6, 1.8 Hz, 1H), 8.02 (d, J = 8.1 Hz, 1H), 7.97 (d, J = 8.6 Hz, 1H), 7.93 (d, J = 8.1 Hz, 1H), 7.66 (ddd, J = 8.1, 6.8, 1.3 Hz, 1H), 7.60 (ddd, J = 8.0, 6.8, 1.2 Hz, 1H), 7.34 (td, J = 7.8, 7.3, 1.7 Hz, 4H), 7.30 (dd, J = 7.4, 2.0 Hz, 2H), 7.24 (ddd, J = 11.1, 6.9, 1.8 Hz, 4H), 5.03 (dd, J = 13.6, 8.1 Hz, 1H), 4.79 (dd, J = 13.6, 7.9 Hz, 1H), 4.69 (d, J = 15.1 Hz, 1H), 4.63 (t, J = 8.0 Hz, 1H), 4.53 (d, J = 15.1 Hz, 1H), 3.73 (d, J = 4.2 Hz, 2H).**^{13}C NMR (150 MHz, CDCl$_3$)** δ 164.49, 164.11, 140.85, 136.45, 136.33, 136.20, 135.84, 133.11, 132.60, 129.90, 129.79, 129.27, 129.12, 128.89, 128.83, 128.26, 128.08, 127.88, 127.21, 125.70, 125.03, 76.98, 48.97, 46.95, 42.77.

ESI HRMS: calcd. For $C_{30}H_{24}N_2O_5$ $[M+H]^+$ 493.1758, found 493.1767.

HPLC Analysis: ee 80%, Chiralpak IF column, *n*-hexane/iPrOH 70:30, flow rate 1.0 mL/min, λ = 254 nm (t_{major} = 35.9 min, t_{minor} = 40.3 min).

(*R*)-1-Benzyl-4-(2-nitro-1-phenylethyl)-2-oxo-2,5-dihydro-1*H*-pyrrol-3-yl 3-phenylpropanoate (3m)

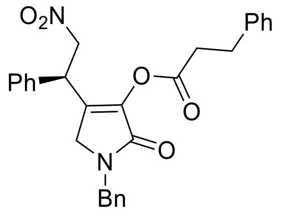

3m

Colourless semi solid, 65% (31mg) yield. **^{1}H NMR (600 MHz, CDCl$_3$)** δ 7.33 – 7.27 (m, 10H), 7.21 – 7.16 (m, 3H), 7.11 – 7.07 (m, 2H), 4.62 (dtd, J = 21.6, 13.5, 8.0 Hz, 3H), 4.50 – 4.40 (m, 2H), 3.60 (d, J = 8.6 Hz, 2H), 3.09 (t, J = 7.6 Hz, 2H), 3.00 – 2.95 (m, 2H).**^{13}C NMR (100 MHz, CDCl$_3$)** δ 170.14, 164.41, 140.40, 139.97, 136.38, 135.85, 135.82, 129.71, 129.11, 128.89, 128.86, 128.59, 128.20, 128.08, 127.81, 126.81, 77.44, 48.85, 46.91, 42.58, 35.47, 30.79.

ESI HRMS: calcd. For $C_{28}H_{26}N_2O_5$ $[M+H]^+$ 479.1914, found 479.1915.

HPLC Analysis: ee 72%, Chiralpak IF column, *n*-hexane/iPrOH 80:20, flow rate 1.0 mL/min, λ = 220 nm (t_{major} = 37.3 min, t_{minor} = 35.4 min).

(*R*)-1-benzyl-4-(2-nitro-1-phenylethyl)-2-oxo-2,5-dihydro-1H-pyrrol-3-yl cyclohexanecarboxylate (3n)

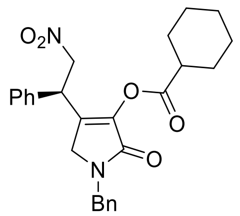

3n

Pale yellow semi solid, 70% (31mg) yield. **^{1}H NMR (600 MHz, CDCl$_3$)** δ 7.36 – 7.29 (m, 5H), 7.28 – 7.26 (m, 1H), 7.21 – 7.14 (m, 4H), 4.95 – 4.87 (m, 1H), 4.80 – 4.73 (m, 1H), 4.66 – 4.58 (m, 1H), 4.54 – 4.45 (m, 2H), 3.67 – 3.56 (m, 2H), 2.68 – 2.60 (m, 1H), 2.15 – 2.07 (m, 2H), 1.89 – 1.80 (m, 2H), 1.72 – 1.65 (m, 2H), 1.43 – 1.27 (m, 4H). **^{13}C NMR (150 MHz, CDCl$_3$)** δ 173.28, 164.47, 140.72, 136.44, 136.00, 135.48, 129.74, 129.08, 128.85, 128.20, 128.03, 127.83, 48.84, 46.88, 42.92, 42.64, 29.05, 25.81, 25.43.

ESI HRMS: calcd. For $C_{26}H_{28}N_2O_5$ $[M+H]^+$ 449.2071, found 449.2072.

HPLC Analysis: ee 72%, Chiralpak IA column, *n*-hexane/iPrOH 80/20, flow rate 1.0 mL/min, λ = 220 nm (t_{major} = 16.7 min, t_{minor} = 15.5 min).

(*R*)-1-Benzyl-4-(2-nitro-1-(*p*-tolyl)ethyl)-2-oxo-2,5-dihydro-1*H*-pyrrol-3-yl benzoate (3o)

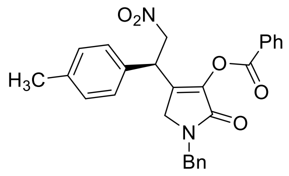

3o

Light yellow semi solid, 83% (38mg) yield. **^{1}H NMR (600 MHz, CDCl$_3$)** δ 8.17 (dd, J = 8.3, 1.2 Hz, 2H), 7.72 – 7.64 (m, 1H), 7.53 (dd, J = 10.8, 4.8 Hz, 2H), 7.36 – 7.29 (m, 3H), 7.23 – 7.19 (m, 4H), 7.06 – 6.99 (m, 2H), 4.97 (dd, J = 13.4, 7.7 Hz, 1H), 4.77 – 4.65 (m, 2H), 4.59 – 4.49 (m, 2H), 3.69 (d, J = 5.2 Hz, 2H), 1.60 (s, 3H). **^{13}C NMR (100 MHz, CDCl$_3$)** δ 164.30, 163.93, 140.90, 136.38, 135.81, 134.69, 131.66, 131.63, 130.90, 129.69, 129.61, 129.14, 129.01, 128.28, 128.13, 127.80, 116.93, 116.71, 77.02, 48.90, 46.98, 42.09.

ESI HRMS: calcd. For $C_{27}H_{24}N_2O_5$ [M+H]$^+$ 457.1758, found 457.1766.

HPLC Analysis: ee 72%, Chiralpak IA column, *n*-hexane/iPrOH 80/20, flow rate 1.0 mL/min, λ = 254 nm (t_{major} = 24.5 min, t_{minor} = 21.4 min).

(*R*)-1-Benzyl-4-(1-(4-(*tert*-butyl)phenyl)-2-nitroethyl)-2-oxo-2,5-dihydro-1*H*-pyrrol-3-yl benzoate (3p)

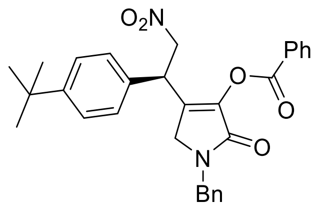

3p

White semi solid, 72% (40mg) yield. **^{1}H NMR (400 MHz, CDCl$_3$)** δ 8.21 – 8.15 (m, 2H), 7.71 – 7.62 (m, 1H), 7.53 (t, J = 7.8 Hz, 2H), 7.38 – 7.27 (m, 5H), 7.24 – 7.19 (m, 2H), 7.14 (d, J = 8.3 Hz, 2H), 4.97 (dd, J = 13.5, 8.2 Hz, 1H), 4.75 (dd, J = 13.5, 7.7 Hz, 1H), 4.67 (d, J = 15.1 Hz, 1H), 4.55 (dd, J = 19.1, 11.5 Hz, 2H), 3.73 (s, 2H), 1.27 (s, 10H). **^{13}C NMR (100 MHz, CDCl$_3$)** δ 164.50, 163.86, 151.91, 140.68, 136.54, 136.42, 134.51, 132.67, 130.89, 129.09, 128.94, 128.27, 128.04, 127.99, 127.51, 126.67, 77.07, 49.00, 46.94, 42.28, 31.38.

ESI HRMS: calcd. For $C_{30}H_{30}N_2O_5$ [M+H]$^+$ 499.2227, found 499.2228.

HPLC Analysis: ee 72%, Chiralpak ADH column, *n*-hexane/iPrOH 80:20, flow rate 1.0 mL/min, λ = 254 nm (t_{major} = 10.5 min, t_{minor} = 11.8 min).

(*R*)-1-Benzyl-4-(1-(4-fluorophenyl)-2-nitroethyl)-2-oxo-2,5-dihydro-1*H*-pyrrol-3-yl benzoate (3q)

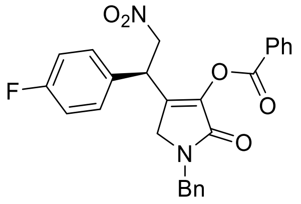

3q

Pale yellow semi solid, 80% (37mg) yield. **^{1}H NMR (400 MHz, CDCl$_3$)** δ 8.21 – 8.14 (m, 2H), 7.71 – 7.65 (m, 1H), 7.54 (t, J = 7.8 Hz, 2H), 7.38 – 7.27 (m, 3H), 7.25 – 7.17 (m, 4H), 7.10 – 6.96 (m, 2H), 4.97 (dd, J = 13.4, 7.6 Hz, 1H), 4.75 (dd, J = 13.4, 8.3 Hz, 1H), 4.68 (d, J = 15.0 Hz, 1H), 4.57 (t, J = 8.0 Hz, 1H), 4.52

(d, J = 15.0 Hz, 1H), 3.75 – 3.63 (m, 2H).^{13}**C NMR (125 MHz, CDCl$_3$)** δ 164.10, 163.73, 163.67, 161.70, 140.77, 136.21, 135.60, 134.46, 131.51, 131.48, 130.70, 129.49, 129.42, 128.95, 128.81, 128.09, 127.94, 127.68, 116.71, 116.54, 48.71, 46.82, 41.92, 29.71.

ESI HRMS: calcd. For $C_{26}H_{21}FN_2O_5$ $[M+H]^+$ 461.1507, found 461.1513.

HPLC Analysis: ee 84%, Chiralpak IF column, *n*-hexane/iPrOH 80:20, flow rate 1.0 mL/min, λ = 254 nm (t_{major} = 33.3 min, t_{minor} = 42.0 min).

(*R*)-1-Benzyl-4-(1-(4-chlorophenyl)-2-nitroethyl)-2-oxo-2,5-dihydro-1*H*-pyrrol-3-yl benzoate (3r)

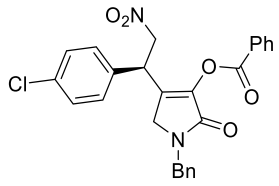

3r

Yellow semi solid, 79% (38mg) yield. ^{1}**H NMR (400 MHz, CDCl$_3$)** δ 8.16 (dd, J = 8.2, 1.0 Hz, 2H), 7.68 (dd, J = 10.6, 4.3 Hz, 1H), 7.53 (t, J = 7.8 Hz, 2H), 7.37 – 7.28 (m, 5H), 7.25 – 7.14 (m, 4H), 4.96 (dd, J = 13.5, 7.6 Hz, 1H), 4.75 (dd, J = 13.5, 8.3 Hz, 1H), 4.67 (d, J = 15.0 Hz, 1H), 4.60 – 4.49 (m, 2H), 3.69 (d, J = 4.4 Hz, 2H).^{13}**C NMR (100 MHz, CDCl$_3$)** δ 164.26, 163.91, 141.07, 136.35, 135.51, 135.00, 134.71, 134.33, 130.89, 130.00, 129.25, 129.16, 129.02, 128.30, 128.16, 76.79, 48.88, 47.01, 42.15.

ESI HRMS: calcd. For $C_{26}H_{21}ClN_2O_5$ $[M+H]^+$ 477.1212, found 477.1224.

HPLC Analysis: ee 76%, Chiralpak ADH column, *n*-hexane/iPrOH 70:30, flow rate 1.0 mL/min, λ = 254 nm (t_{major} = 12.7 min, t_{minor} = 14.8 min).

(*R*)-1-Benzyl-4-(1-(4-bromophenyl)-2-nitroethyl)-2-oxo-2,5-dihydro-1*H*-pyrrol-3-yl benzoate (3s)

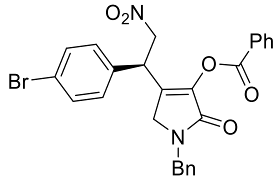

3s

Yellow semi solid, 82% (76mg) yield. ^{1}**H NMR (400 MHz, CDCl$_3$)** δ 8.20 – 8.13 (m, 2H), 7.68 (t, J = 7.5 Hz, 1H), 7.54 (t, J = 7.8 Hz, 2H), 7.49 – 7.42 (m, 2H), 7.39 – 7.28 (m, 3H), 7.25 – 7.19 (m, 2H), 7.11 (d, J = 8.4 Hz, 2H), 4.96 (dd, J = 13.5, 7.6 Hz, 1H), 4.75 (dd, J = 13.5, 8.3 Hz, 1H), 4.67 (d, J = 15.0 Hz, 1H), 4.59 – 4.49 (m, 2H), 3.69 (d, J = 4.4 Hz, 2H).^{13}**C NMR (100 MHz, CDCl$_3$)** δ 164.24, 163.90, 141.10, 136.34, 135.40, 134.84, 134.72, 132.97, 130.90, 129.55, 129.17, 129.03, 128.30, 128.17, 127.75, 123.10, 76.71, 48.88, 47.00, 42.22.

ESI HRMS: calcd. For $C_{26}H_{21}BrN_2O_5$ $[M+H]^+$ 521.0707, found 521.0726.

HPLC Analysis: ee 76%, Chiralpak ADH column, *n*-hexane/iPrOH 80:20, flow rate 1.0 mL/min, λ = 254 nm (t_{major} = 25.6 min, t_{minor} = 21.3 min).

(*S*)-1-Benzyl-4-(1-(2-fluorophenyl)-2-nitroethyl)-2-oxo-2,5-dihydro-1*H*-pyrrol-3-yl benzoate (3t)

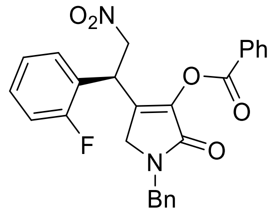

3t

Yellow semi solid, 79% (36mg) yield. **^{1}H NMR (400 MHz, CDCl$_3$)** δ 8.15 (dd, J = 8.3, 1.1 Hz, 2H), 7.70 – 7.63 (m, 1H), 7.52 (t, J = 7.8 Hz, 2H), 7.38 – 7.27 (m, 4H), 7.26 – 7.19 (m, 3H), 7.12 – 7.03 (m, 2H), 5.01 (dd, J = 11.4, 6.1 Hz, 1H), 4.91 – 4.80 (m, 2H), 4.67 (d, J = 15.0 Hz, 1H), 4.56 (d, J = 15.0 Hz, 1H), 3.78 (s, 2H).**^{13}C NMR (100 MHz, CDCl$_3$)** δ 164.30, 163.63, 161.82, 159.36, 141.37, 136.44, 134.97, 134.54, 130.87, 130.80, 130.72, 129.62, 129.58, 129.14, 128.93, 128.27, 128.10, 127.90, 125.45, 125.41, 122.78, 122.64, 116.71, 116.49, 75.41, 75.38, 48.95, 46.99, 36.80, 36.77.

ESI HRMS: calcd. For $C_{26}H_{21}FN_2O_5$ $[M+H]^+$ 461.1507, found 461.1516.

HPLC Analysis: ee 86%, Chiralpak ID column, *n*-hexane/iPrOH 75:25, flow rate 1.0 mL/min, λ = 254 nm (t_{major} = 56.1 min, t_{minor} = 68.1 min).

(*S*)-1-Benzyl-4-(1-(2,4-difluorophenyl)-2-nitroethyl)-2-oxo-2,5-dihydro-1*H*-pyrrol-3-yl benzoate (3u)

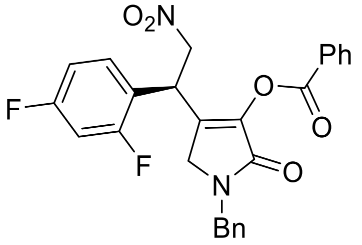

3u

White sticky solid, 78% (37mg) yield. **^{1}H NMR (400 MHz, CDCl$_3$)** δ 8.18 – 8.09 (m, 2H), 7.70 – 7.64 (m, 1H), 7.52 (t, J = 7.8 Hz, 2H), 7.39 – 7.28 (m, 3H), 7.26 – 7.18 (m, 3H), 6.88 – 6.78 (m, 2H), 5.02 – 4.93 (m, 1H), 4.82 (dq, J = 15.5, 7.7 Hz, 2H), 4.67 (d, J = 15.0 Hz, 1H), 4.56 (d, J = 15.0 Hz, 1H), 3.77 (d, J = 4.3 Hz, 2H). **^{13}C NMR (100 MHz, CDCl$_3$)** δ 163.96, 163.45, 141.32, 136.16, 134.43, 134.36, 130.65, 130.33, 130.27, 128.96, 128.77, 128.10, 127.95, 127.59, 118.51, 112.65, 112.44, 105.22, 104.96, 104.71, 75.12, 48.69, 46.81, 36.19, 36.17, 29.70.

ESI HRMS: calcd. For $C_{26}H_{20}F_2N_2O_5$ $[M+H]^+$ 479.1413, found 479.1424.

HPLC Analysis: ee 72%, Chiralpak ADH column, *n*-hexane/iPrOH 70:30, flow rate 1.0 mL/min, λ = 254 nm (t_{major} = 11.4 min, t_{minor} = 10.1 min).

(*R*)-1-Benzyl-4-(1-(3,5-dimethoxyphenyl)-2-nitroethyl)-2-oxo-2,5-dihydro-1*H*-pyrrol-3-yl benzoate (3v)

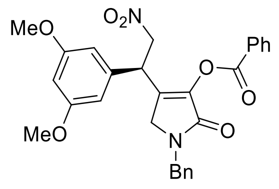

3v

Light yellow semi solid, 80% (40mg) yield. **^{1}H NMR (600 MHz, CDCl₃)** δ 8.20 – 8.15 (m, 2H), 7.68 (t, J = 7.4 Hz, 1H), 7.53 (t, J = 7.8 Hz, 2H), 7.33 (t, J = 7.2 Hz, 2H), 7.29 (dd, J = 8.5, 6.0 Hz, 1H), 7.22 (d, J = 7.0 Hz, 2H), 6.80 – 6.72 (m, 3H), 4.98 (dd, J = 13.5, 8.1 Hz, 1H), 4.73 (dd, J = 13.5, 7.9 Hz, 1H), 4.68 (d, J = 15.1 Hz, 1H), 4.55 – 4.48 (m, 2H), 3.83 (d, J = 3.0 Hz, 6H), 3.71 (d, J = 4.6 Hz, 2H).**^{13}C NMR (150 MHz, $CDCl_3$)** δ 164.48, 164.07, 149.94, 149.37, 140.63, 136.54, 136.48, 134.63, 130.85, 129.11, 129.00, 128.22, 128.08, 127.90, 119.98, 111.81, 110.71, 77.26, 56.20, 56.11, 49.05, 46.94, 42.55.

ESI HRMS: calcd. For $C_{28}H_{26}N_2O_7$ $[M+H]^+$ 503.1813, found 503.1814.

HPLC Analysis: ee 72%, Chiralpak ADH column, *n*-hexane/iPrOH 80:20, flow rate 1.0 mL/min, λ = 254 nm (t_{major} = 37.1 min, t_{minor} = 32.4 min).

(*R*)-1-Benzyl-4-(2-nitro-1-(thiophen-2-yl)ethyl)-2-oxo-2,5-dihydro-1*H*-pyrrol-3-yl benzoate (3w)

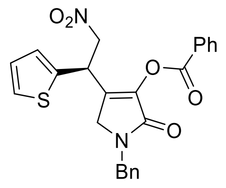

3w

Light brown semi solid, 81% (36mg) yield. **^{1}H NMR (400 MHz, $CDCl_3$)** δ 8.24 – 8.14 (m, 2H), 7.67 (dd, J = 10.6, 4.3 Hz, 1H), 7.53 (t, J = 7.8 Hz, 2H), 7.38 – 7.29 (m, 3H), 7.27 – 7.22 (m, 3H), 6.94 (d, J = 3.4 Hz, 2H), 4.98 – 4.91 (m, 2H), 4.85 – 4.79 (m, 1H), 4.63 (d, J = 3.4 Hz, 2H), 3.81 (d, J = 5.0 Hz, 2H).**^{13}C NMR (100 MHz, $CDCl_3$)** δ 164.28, 163.57, 140.87, 137.77, 136.42, 135.52, 134.58, 130.92, 130.38, 129.15, 128.97, 128.66, 128.25, 128.11, 127.90, 127.74, 126.70, 126.31, 77.32, 48.63, 47.00, 37.39.

ESI HRMS: calcd. For $C_{24}H_{20}N_2O_5S$ $[M+H]^+$ 449.1166, found 449.1167.

HPLC Analysis: ee 82%, Chiralpak IA column, *n*-hexane/iPrOH 80:20, flow rate 1.0 mL/min, λ = 254 nm (t_{major} = 34.1 min, t_{minor} = 36.9 min).

(*R*)-4-(2-Nitro-1-phenylethyl)-2-oxo-2,5-dihydrofuran-3-yl 4-methylbenzoate (5b)

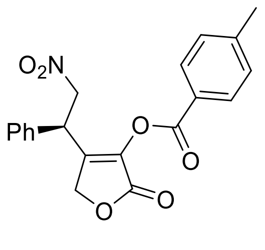

5a

Light yellow semi solid, 82% (30mg) yield. **^{1}H NMR (500 MHz, $CDCl_3$)** δ 8.03 (d, J = 8.2 Hz, 2H), 7.42 – 7.28 (m, 5H), 7.26 (dd, J = 6.6, 2.1 Hz, 2H), 5.04 (dd, J = 13.8, 8.3 Hz, 1H), 4.83 – 4.79 (m, 1H), 4.75 (s, 1H), 4.70 (d, J = 16.8 Hz, 1H), 4.65 (t, J = 7.9 Hz, 1H), 2.46 (s, 3H).**^{13}C NMR (150 MHz, $CDCl_3$)** δ 166.72, 163.37, 146.18, 145.67, 135.90, 134.41, 130.99, 130.07, 129.83, 129.40, 127.88, 124.36, 76.14, 68.64, 42.27, 22.13.

ESI HRMS: calcd. For $C_{20}H_{17}NO_6$ $[M+Na]^+$ 390.0948, found 390.0957.

HPLC Analysis: ee 78%, Chiralpak ADH column, *n*-hexane/iPrOH 85:25, flow rate 1.0 mL/min, λ = 200 nm (t_{major} = 12.5 min, t_{minor} = 13.5 min).

(*R*)-4-(2-Nitro-1-phenylethyl)-2-oxo-2,5-dihydrofuran-3-yl 4-methoxybenzoate (5c)

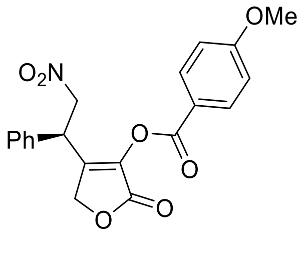

5b

Light yellow semi solid, 85% (33mg) yield. **^{1}H NMR (500 MHz, $CDCl_3$)** δ 8.02 (d, J = 8.5 Hz, 2H), 7.30 (dd, J = 13.0, 7.0 Hz, 3H), 7.22 – 7.16 (m, 2H), 6.93 (d, J = 8.5 Hz, 2H), 4.97 (dd, J = 13.9, 8.4 Hz, 1H), 4.76 – 4.55 (m, 4H), 3.83 (s, 3H).**^{13}C NMR (125 MHz, $CDCl_3$)** δ 166.74, 165.07, 162.99, 145.55, 134.61, 133.22, 130.04, 129.35, 127.90, 119.47, 114.45, 76.21, 68.58, 55.84, 42.32.

ESI HRMS: calcd. For $C_{20}H_{17}NO_7$ $[M+H]^+$ 384.1078, found 384.1087.

HPLC Analysis: ee 78%, Chiralpak ADH column, *n*-hexane/iPrOH 85:15, flow rate 1.0 mL/min, λ = 274 nm (t_{major} = 19.1 min, t_{minor} = 20.8 min).

6. NMR spectra of the products

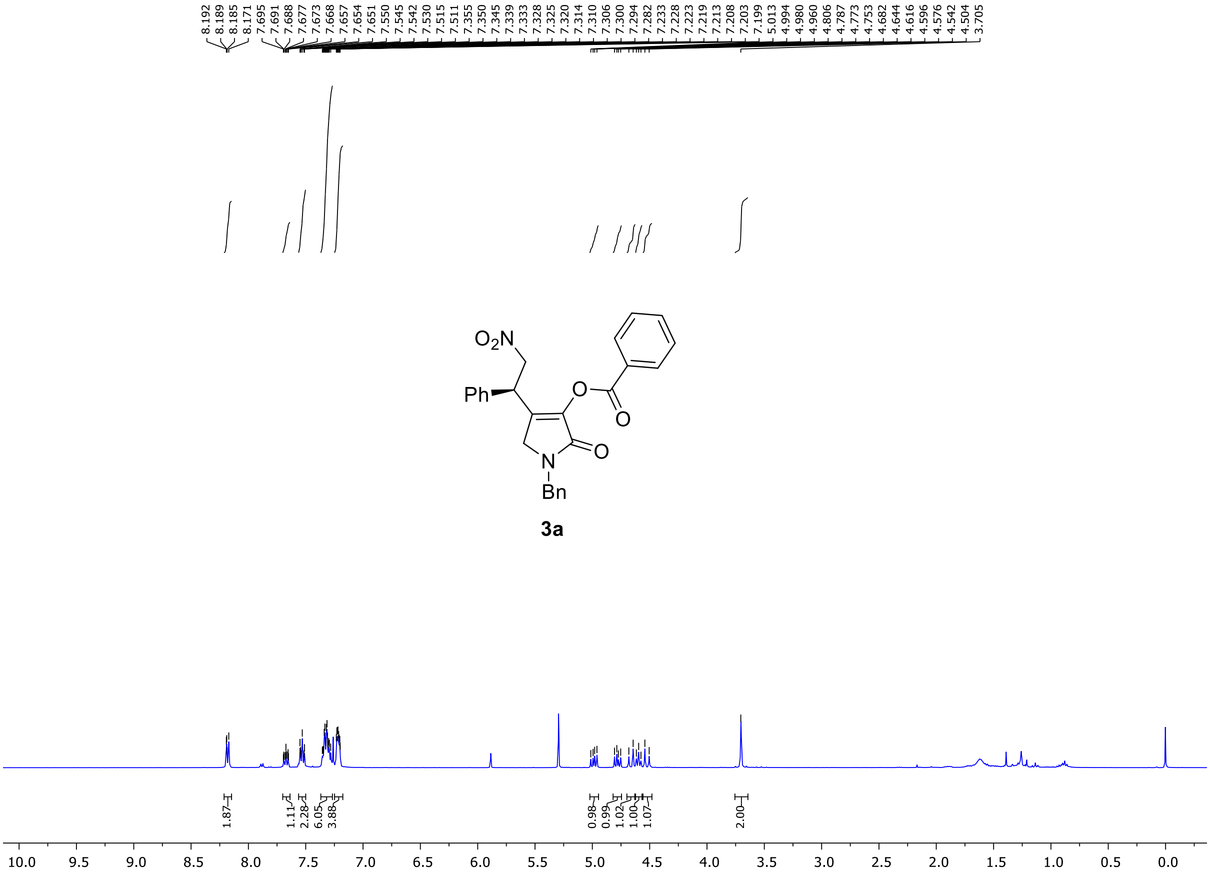

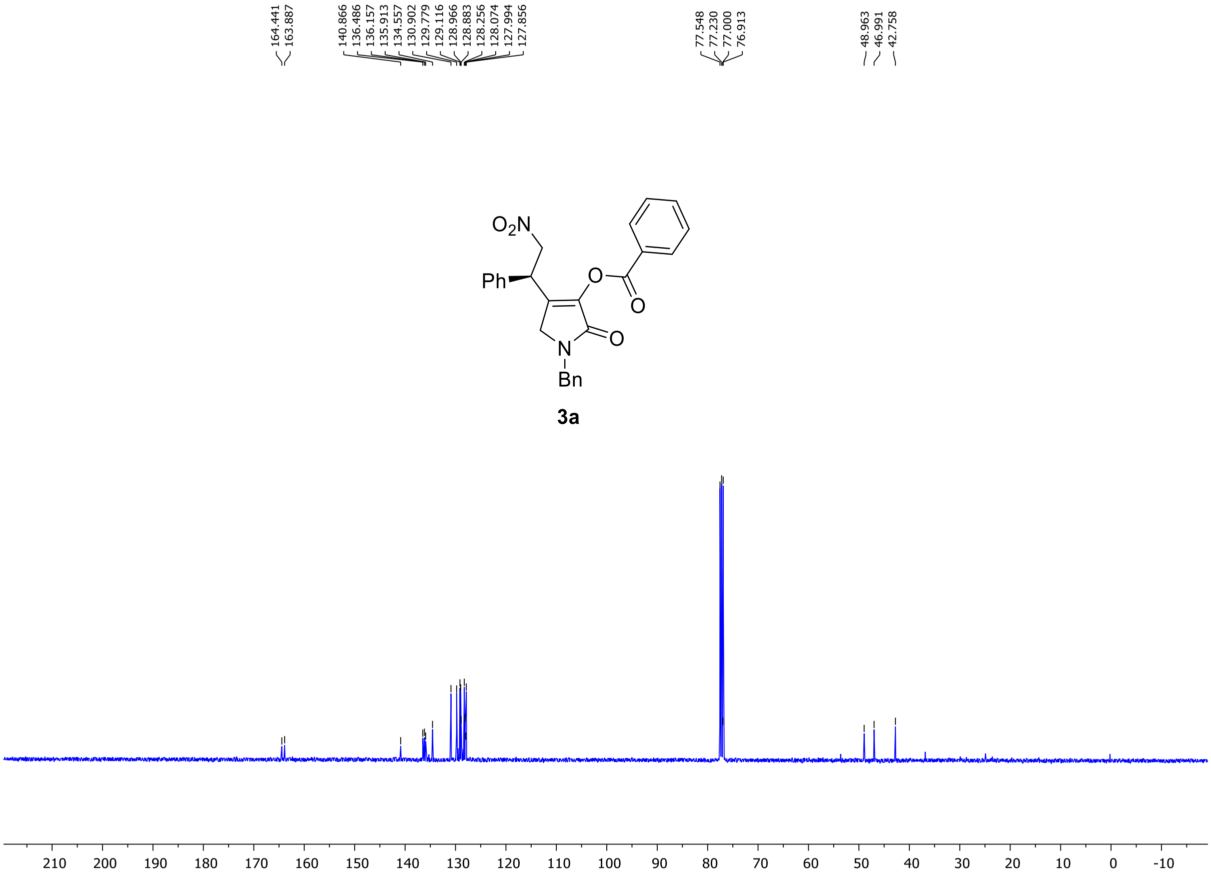

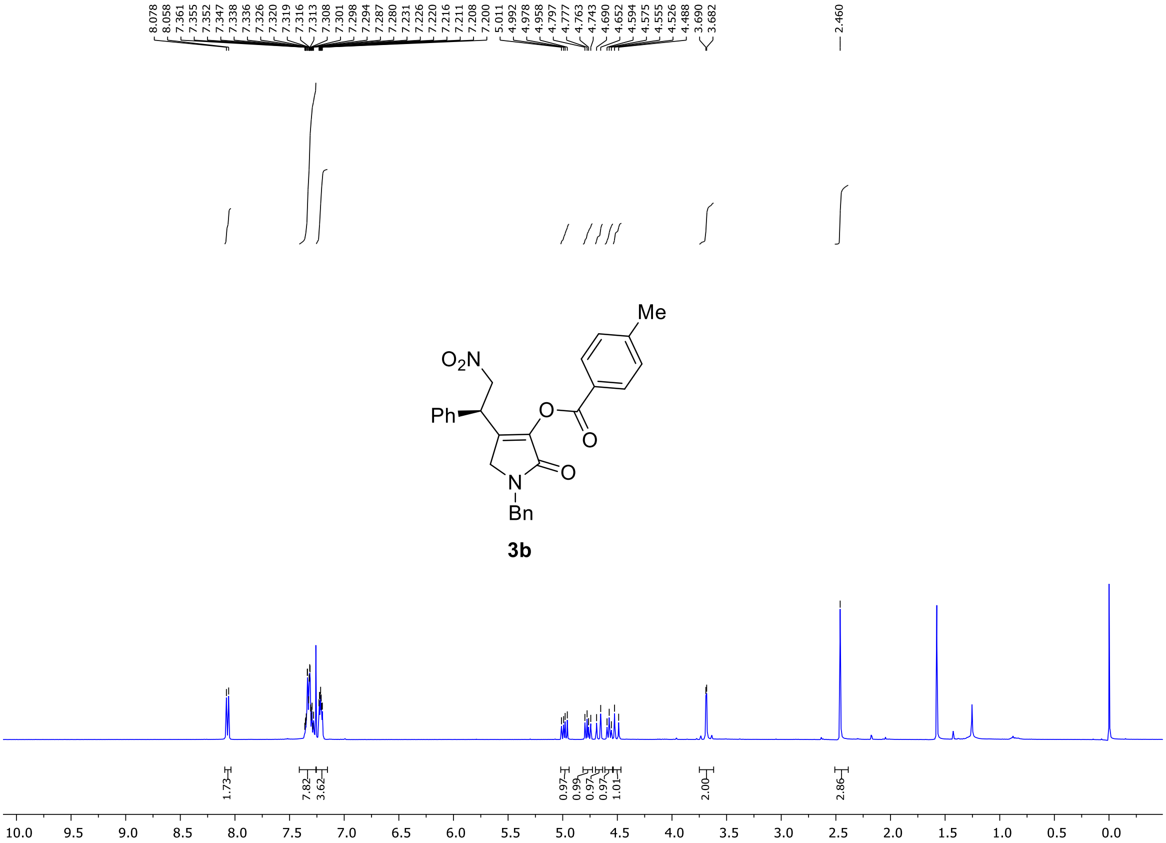
8.078 8.058 7.361 7.355 7.352 7.347 7.338 7.336 7.326 7.320 7.319 7.316 7.313 7.308 7.301 7.298 7.294 7.287 7.280 7.231 7.226 7.220 7.216 7.211 7.208 7.200 5.011 4.992 4.978 4.958 4.797 4.777 4.763 4.743 4.690 4.652 4.594 4.575 4.555 4.526 4.488 3.690 3.682
2.460
Me
O_2N
Ph
O
O
O
N
Bn
3b
1.73
7.82
3.62
0.97
0.99
0.97
0.97
1.01
2.00
2.86
10.0 9.5 9.0 8.5 8.0 7.5 7.0 6.5 6.0 5.5 5.0 4.5 4.0 3.5 3.0 2.5 2.0 1.5 1.0 0.5 0.0

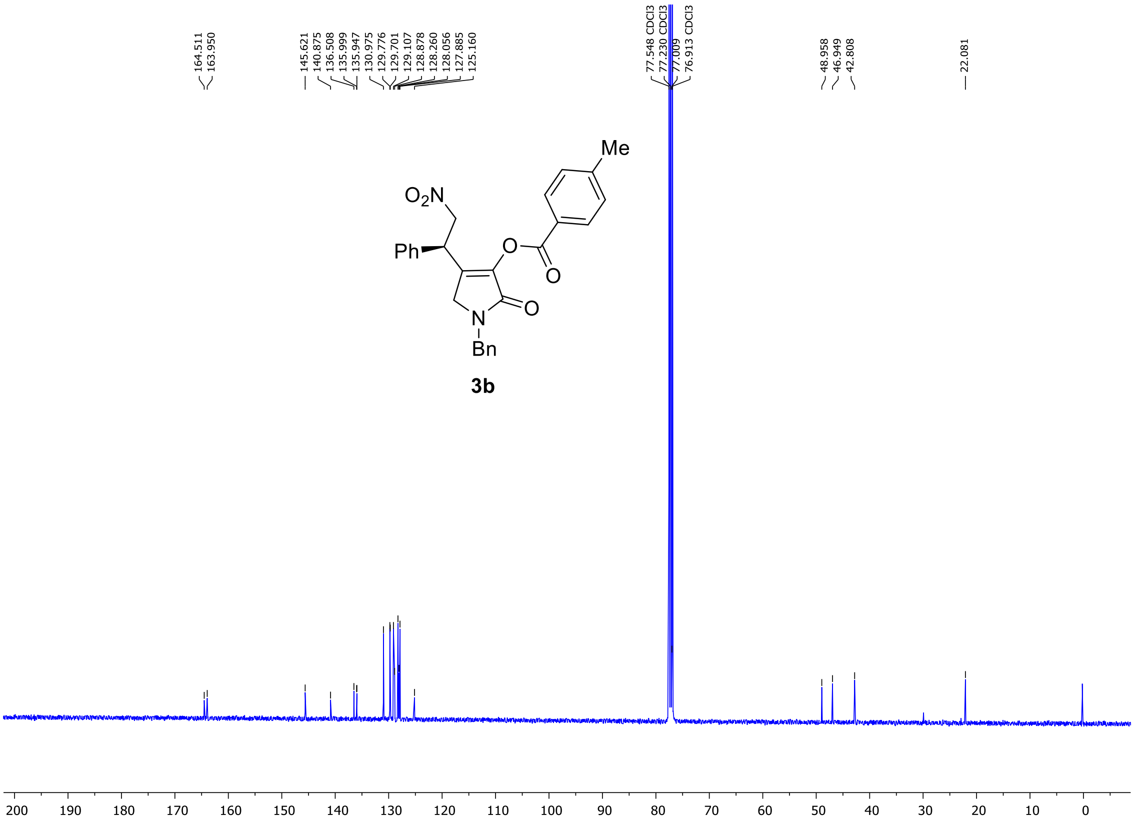
164.511 163.950
145.621 140.875 136.508 135.999 135.947 130.975 129.776 129.701 129.107 128.878 128.260 128.056 127.885 125.160
77.548 CDCl3 77.230 CDCl3 77.009 76.913 CDCl3
48.958 46.949 42.808
22.081
Me
O_2N
Ph
O
O
O
N
Bn
3b
200 190 180 170 160 150 140 130 120 110 100 90 80 70 60 50 40 30 20 10 0

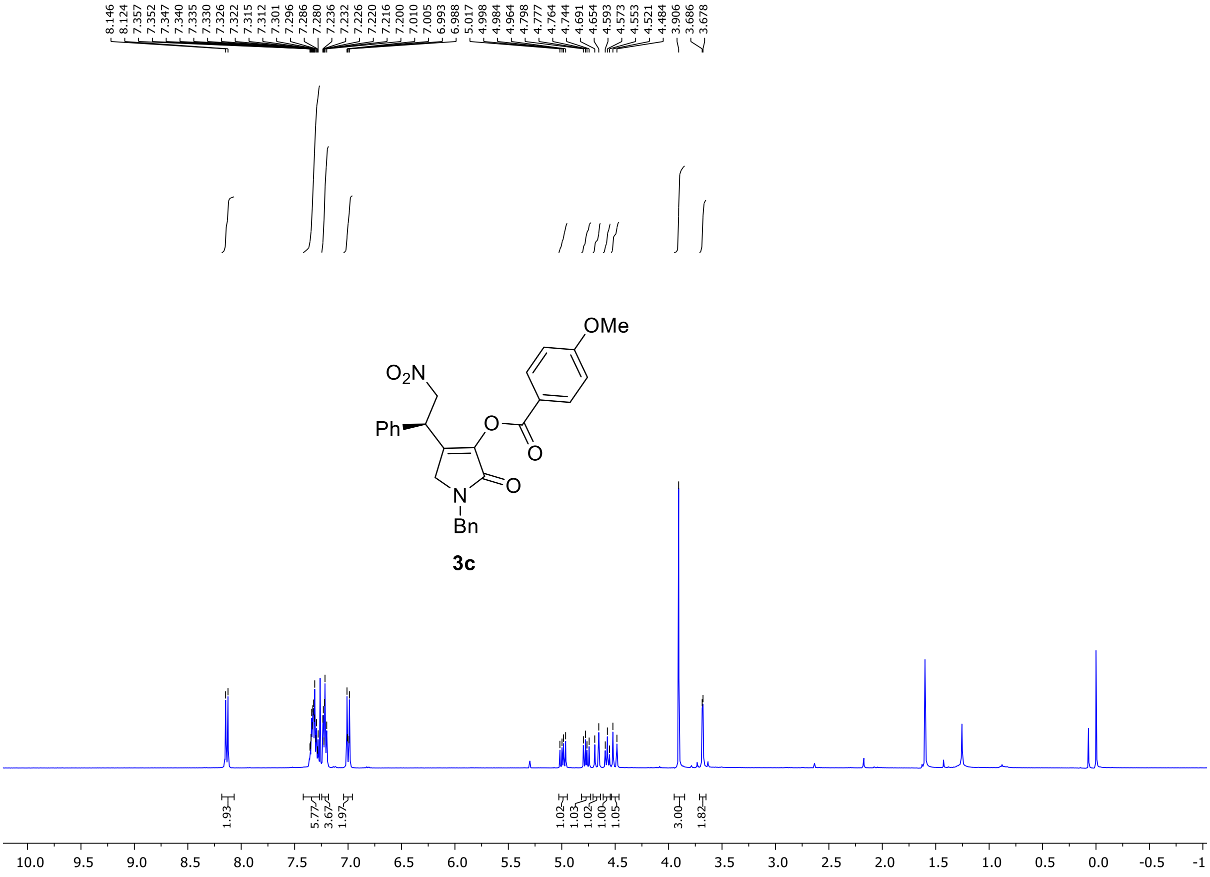

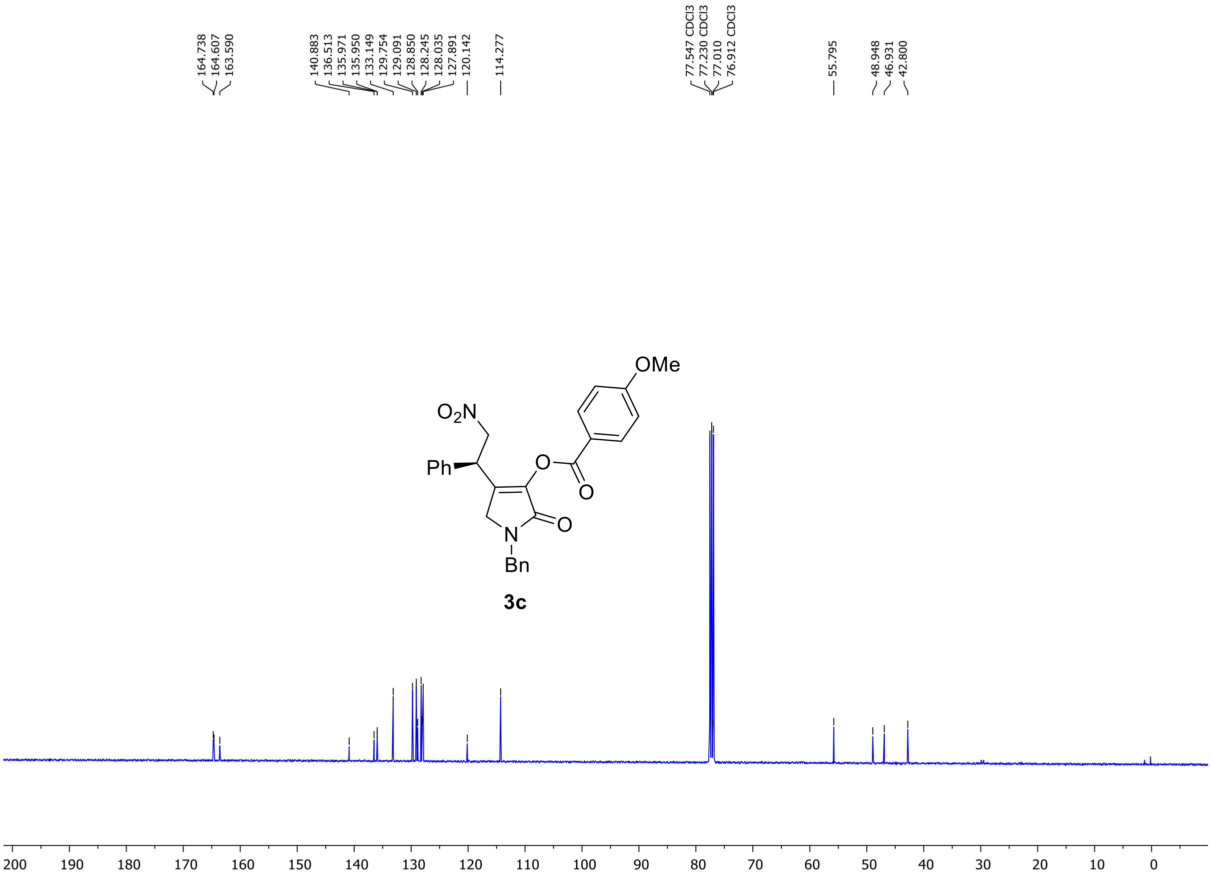

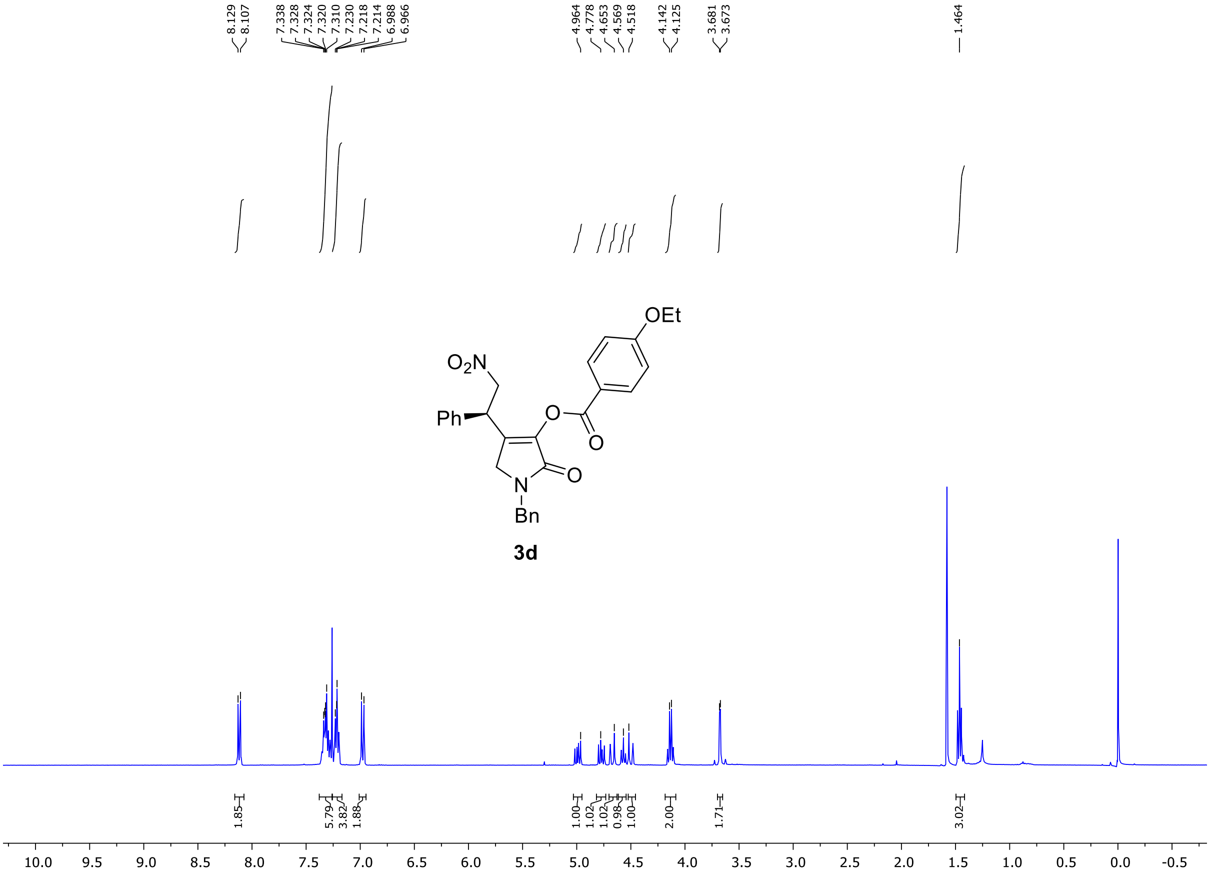
8.129
8.107
7.338
7.328
7.324
7.320
7.310
7.230
7.218
7.214
6.988
6.966
4.964
4.778
4.653
4.569
4.518
4.142
4.125
3.681
3.673
1.464
OEt
O_2N
Ph
O
O
O
N
Bn
3d
1.85
5.79
3.82
1.88
1.00
1.02
1.02
0.98
1.00
2.00
1.71
3.02
10.0
9.5
9.0
8.5
8.0
7.5
7.0
6.5
6.0
5.5
5.0
4.5
4.0
3.5
3.0
2.5
2.0
1.5
1.0
0.5
0.0
-0.5

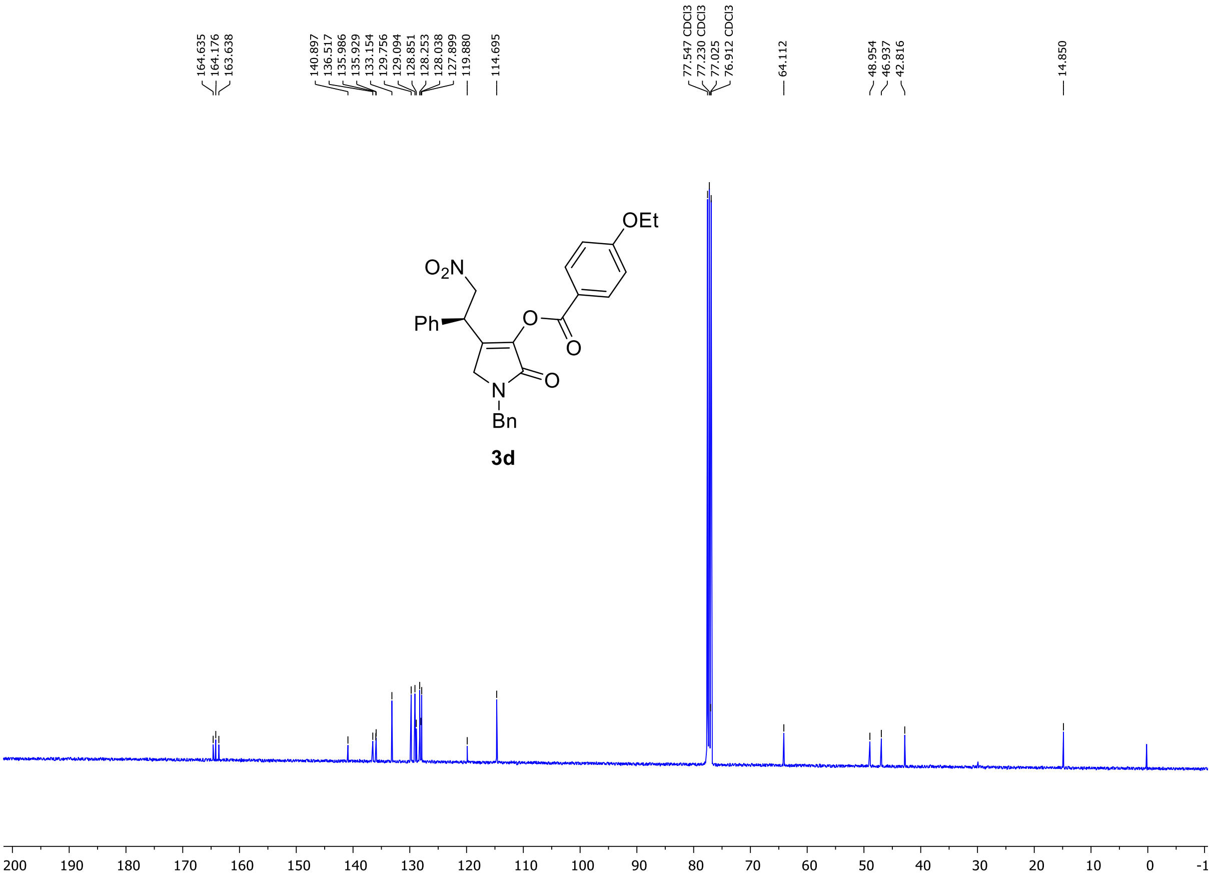
164.635
164.176
163.638
140.897
136.517
135.986
135.929
133.154
129.756
129.094
128.851
128.253
128.038
127.899
119.880
114.695
77.547 CDCl3
77.230 CDCl3
77.025
76.912 CDCl3
64.112
48.954
46.937
42.816
14.850
OEt
O_2N
Ph
O
O
O
N
Bn
3d
200
190
180
170
160
150
140
130
120
110
100
90
80
70
60
50
40
30
20
10
0
-1

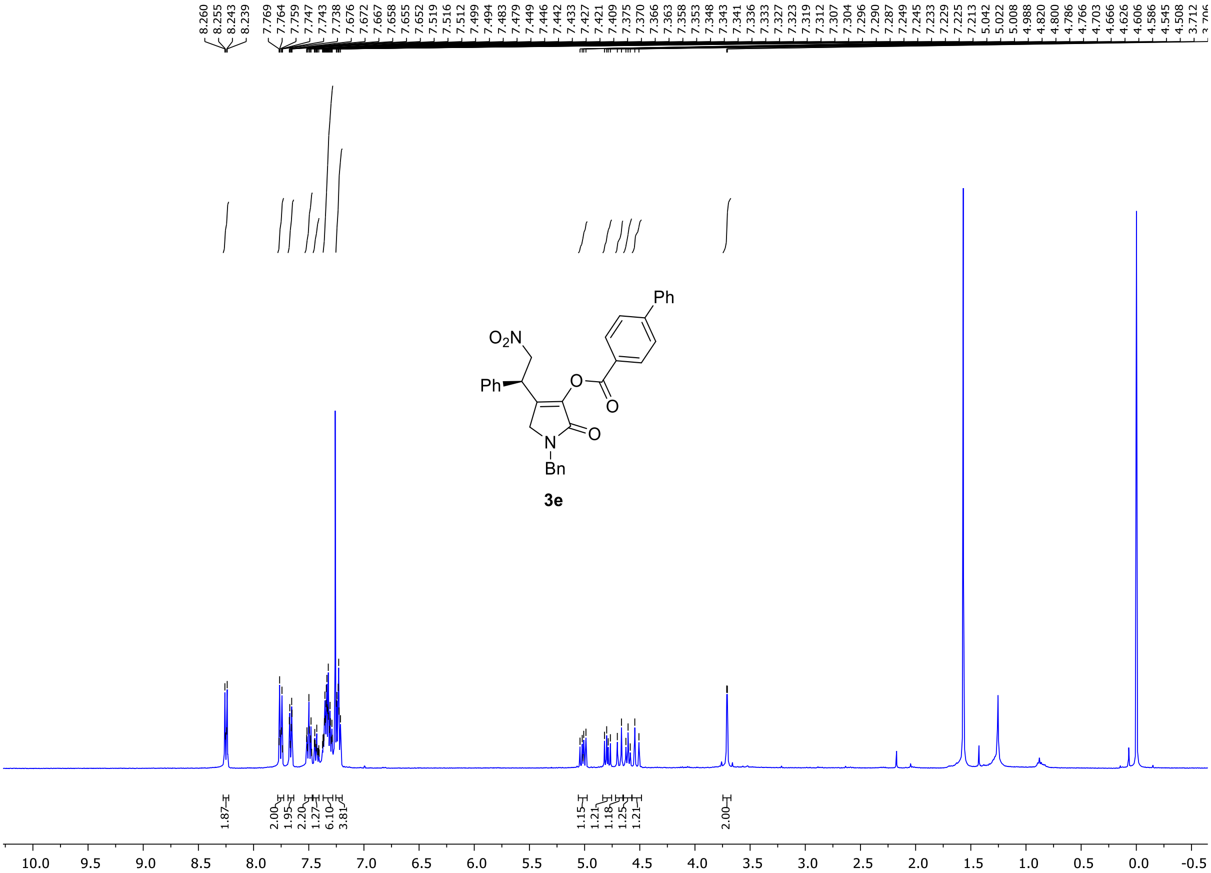
O2N
Ph
O
O
N
O
Bn
Ph
3e

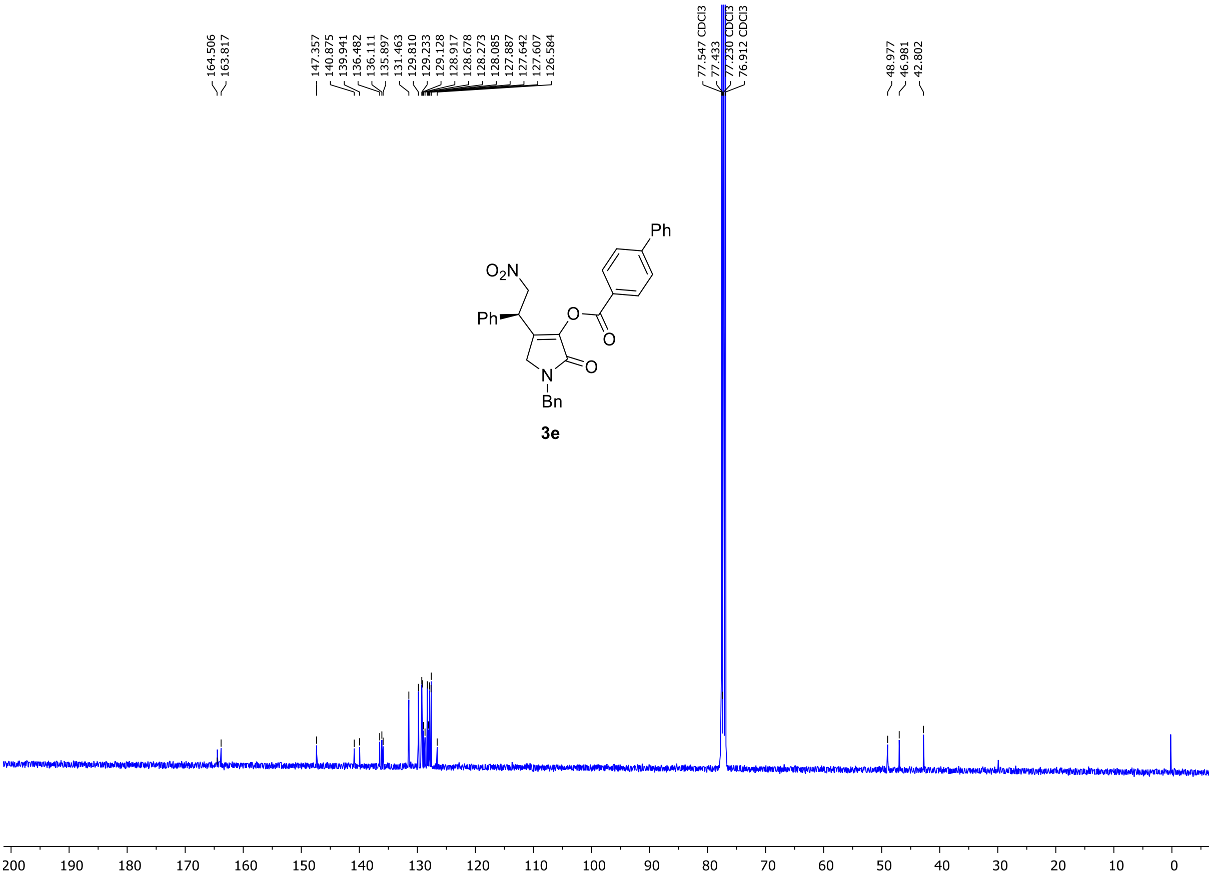
164.506
163.817
147.357
140.875
139.941
136.482
136.111
135.897
131.463
129.810
129.233
129.128
128.917
128.678
128.273
128.085
127.887
127.642
127.607
126.584
77.547 CDCl3
77.433
77.230 CDCl3
76.912 CDCl3
48.977
46.981
42.802
O2N
Ph
O
O
N
O
Bn
Ph
3e

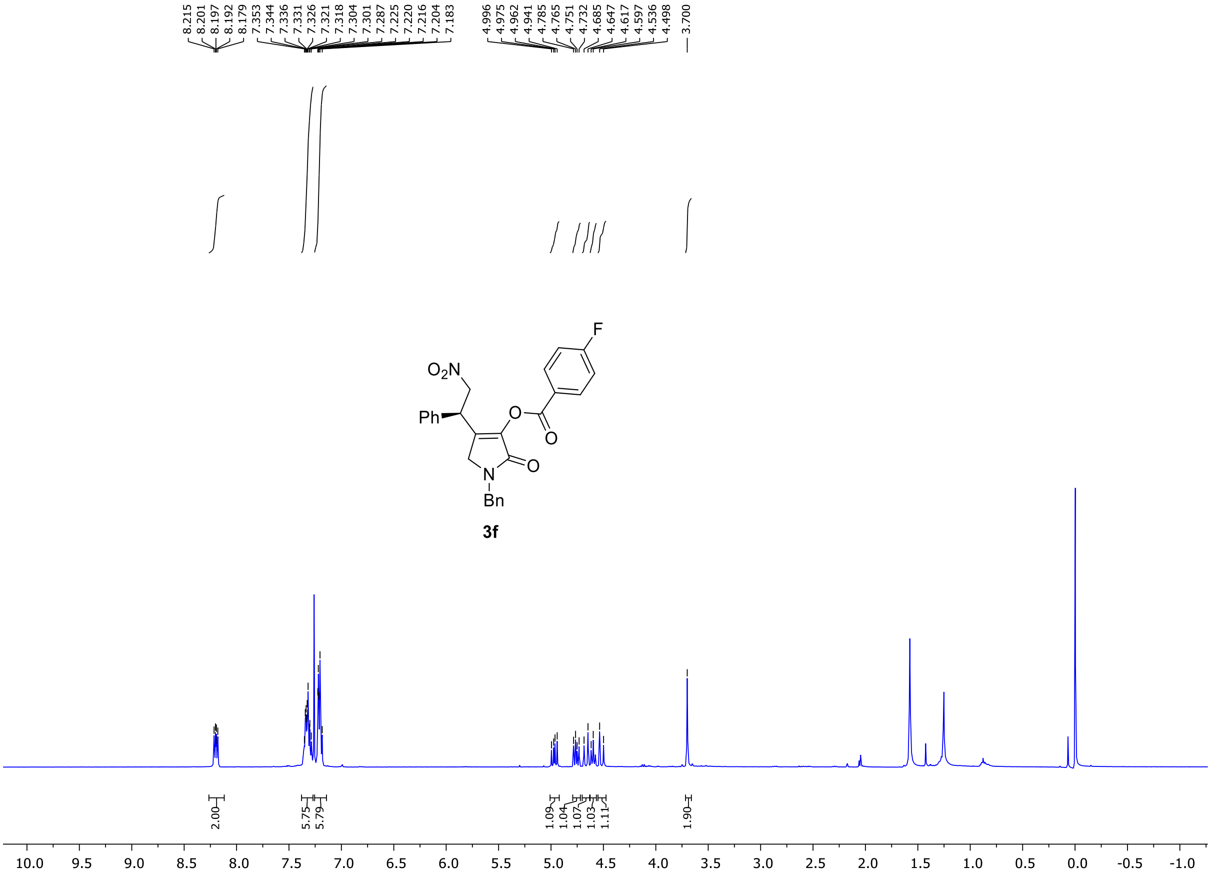
3f

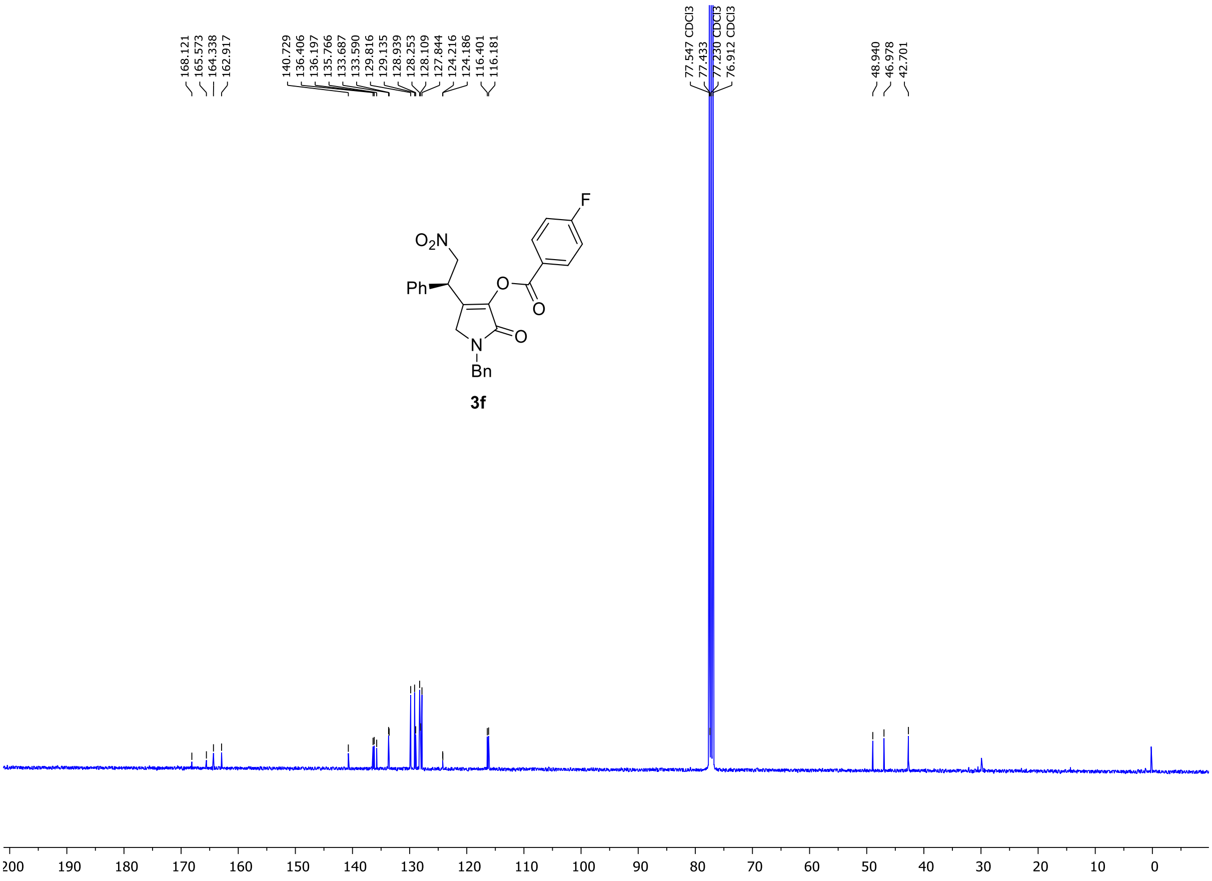
3f

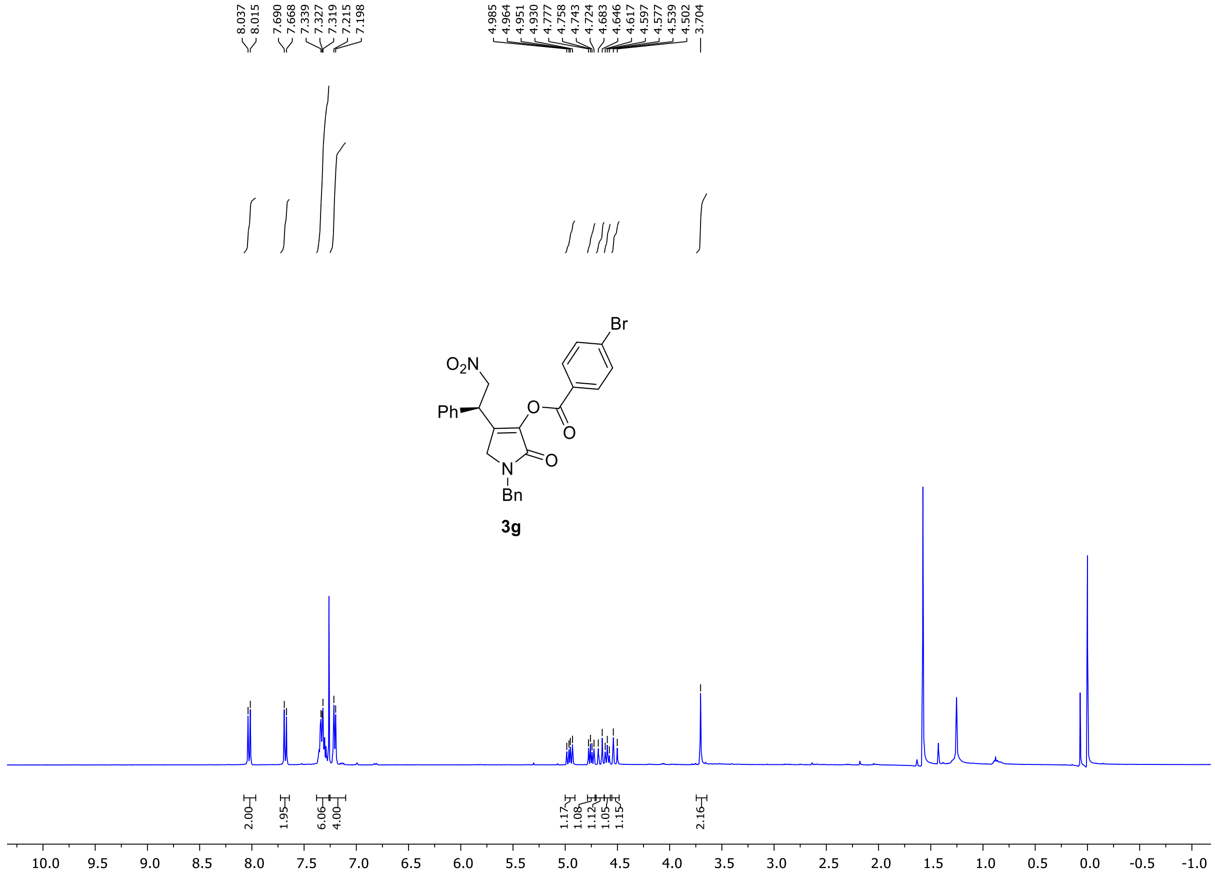
8.037
8.015
7.690
7.668
7.339
7.327
7.319
7.215
7.198
4.985
4.964
4.951
4.930
4.777
4.758
4.743
4.724
4.683
4.646
4.617
4.597
4.577
4.539
4.502
3.704
Br
O2N
Ph
O
O
N
O
Bn
3g
2.00
1.95
6.06
4.00
1.17
1.08
1.12
1.05
1.15
2.16
10.0 9.5 9.0 8.5 8.0 7.5 7.0 6.5 6.0 5.5 5.0 4.5 4.0 3.5 3.0 2.5 2.0 1.5 1.0 0.5 0.0 -0.5 -1.0

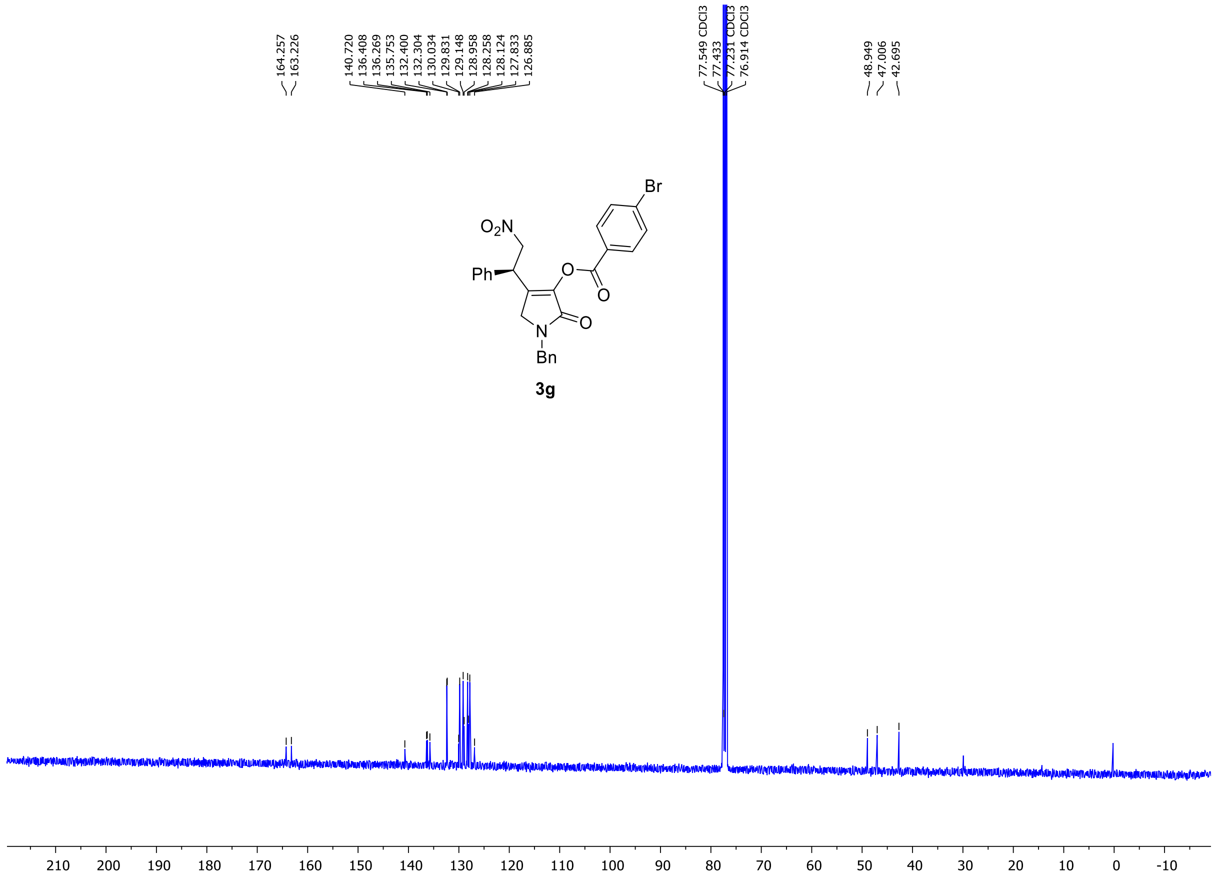
164.257
163.226
140.720
136.408
136.269
135.753
132.400
132.304
130.034
129.831
129.148
128.958
128.258
128.124
127.833
126.885
77.549 CDCl3
77.433
77.231 CDCl3
76.914 CDCl3
48.949
47.006
42.695
Br
O2N
Ph
O
O
N
O
Bn
3g
210 200 190 180 170 160 150 140 130 120 110 100 90 80 70 60 50 40 30 20 10 0 -10

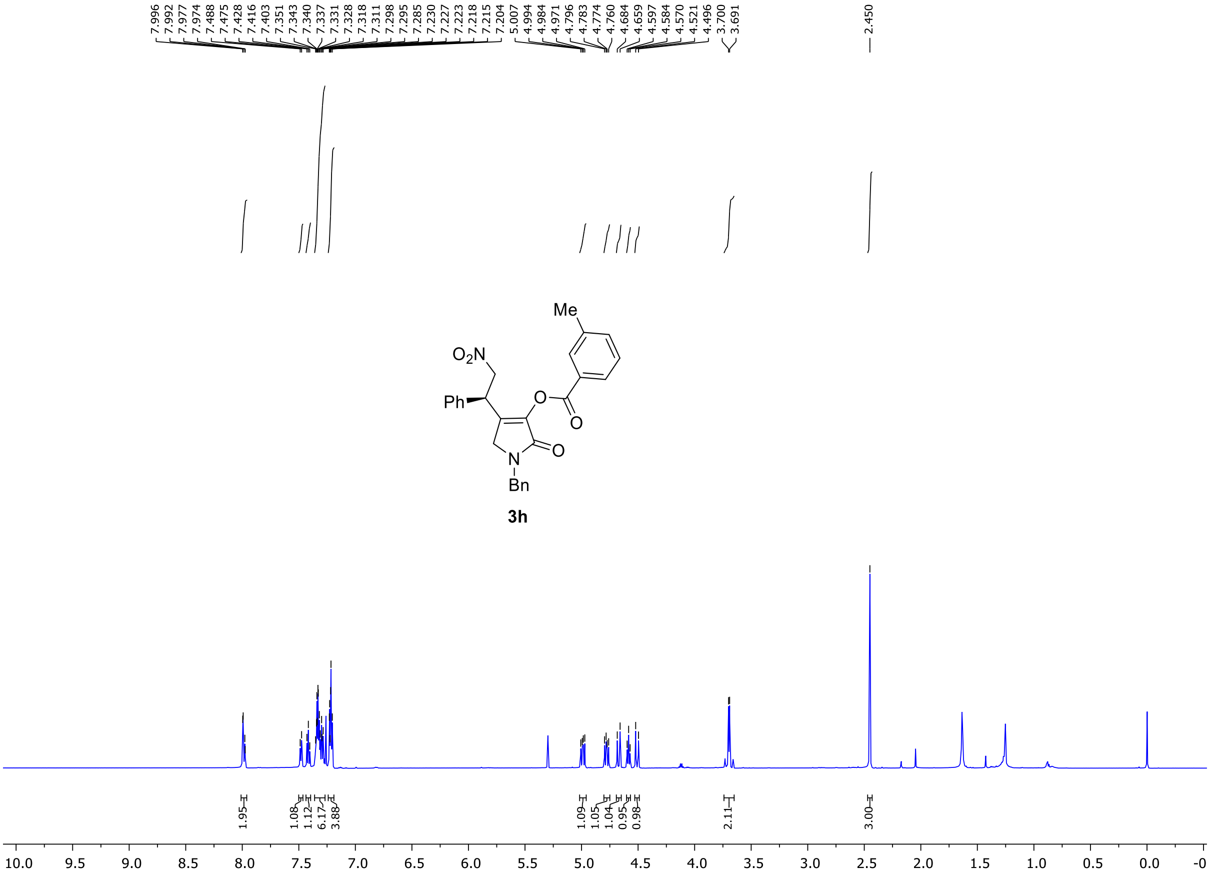

7.996
7.992
7.977
7.974
7.488
7.475
7.428
7.416
7.403
7.351
7.343
7.340
7.337
7.331
7.328
7.318
7.311
7.298
7.295
7.285
7.230
7.227
7.223
7.218
7.215
7.204
5.007
4.994
4.984
4.971
4.796
4.783
4.774
4.760
4.684
4.659
4.597
4.584
4.570
4.521
4.496
3.700
3.691
2.450
Me
O_2N
Ph
O
O
N
Bn
3h
1.95
1.08
1.12
6.17
3.88
1.09
1.05
1.04
0.95
0.98
2.11
3.00
10.0 9.5 9.0 8.5 8.0 7.5 7.0 6.5 6.0 5.5 5.0 4.5 4.0 3.5 3.0 2.5 2.0 1.5 1.0 0.5 0.0 -0

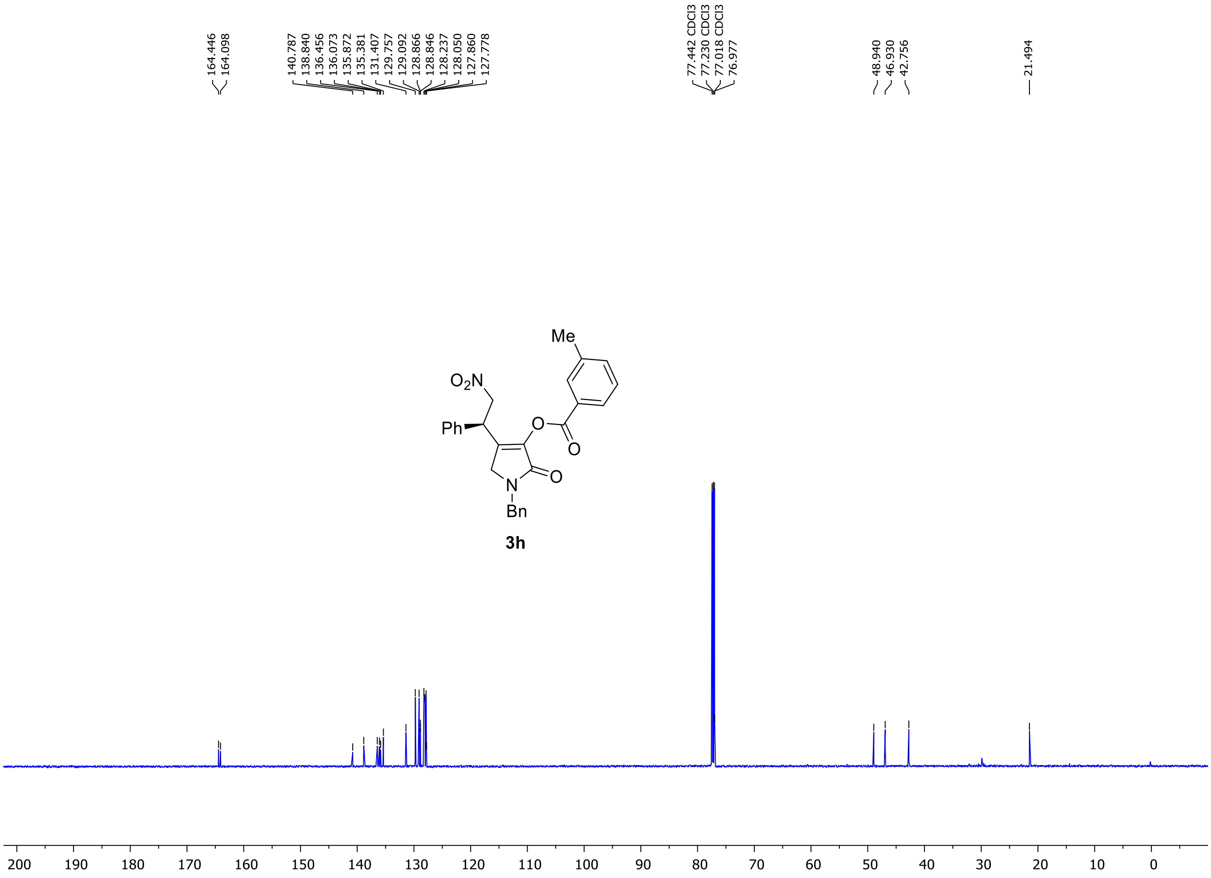

164.446
164.098
140.787
138.840
136.456
136.073
135.872
135.381
131.407
129.757
129.092
128.866
128.846
128.237
128.050
127.860
127.778
77.442 CDCl3
77.230 CDCl3
77.018 CDCl3
76.977
48.940
46.930
42.756
21.494
Me
O_2N
Ph
O
O
N
Bn
3h
200 190 180 170 160 150 140 130 120 110 100 90 80 70 60 50 40 30 20 10 0

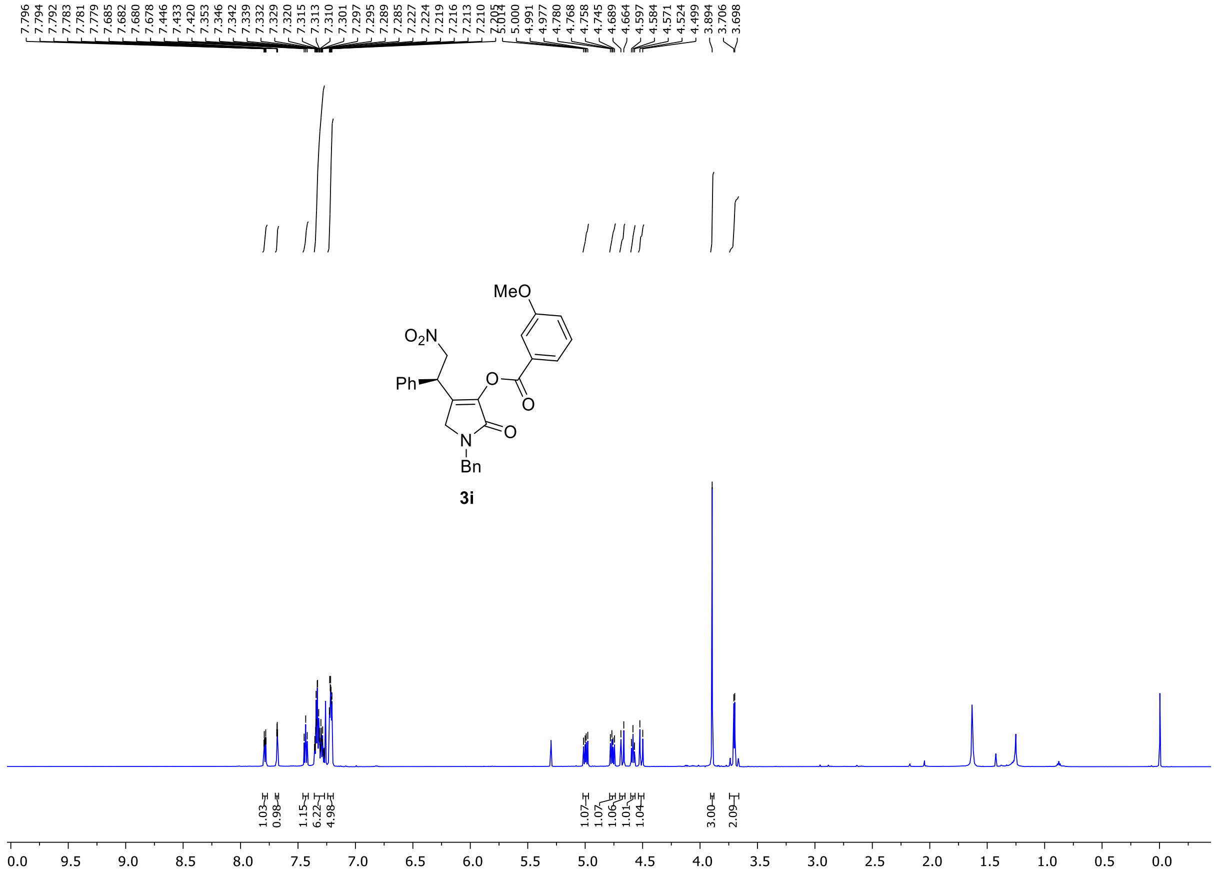
7.796
7.794
7.792
7.783
7.781
7.779
7.685
7.682
7.680
7.678
7.446
7.433
7.420
7.353
7.346
7.342
7.339
7.332
7.329
7.320
7.315
7.313
7.310
7.301
7.297
7.295
7.289
7.285
7.227
7.224
7.219
7.216
7.213
7.210
7.205
5.014
5.000
4.991
4.977
4.780
4.768
4.758
4.745
4.689
4.664
4.597
4.584
4.571
4.524
4.499
3.894
3.706
3.698
MeO
O2N
Ph
O
O
O
N
Bn
3i
1.03
0.98
1.15
6.22
4.98
1.07
1.07
1.06
1.01
1.04
3.00
2.09
0.0 9.5 9.0 8.5 8.0 7.5 7.0 6.5 6.0 5.5 5.0 4.5 4.0 3.5 3.0 2.5 2.0 1.5 1.0 0.5 0.0

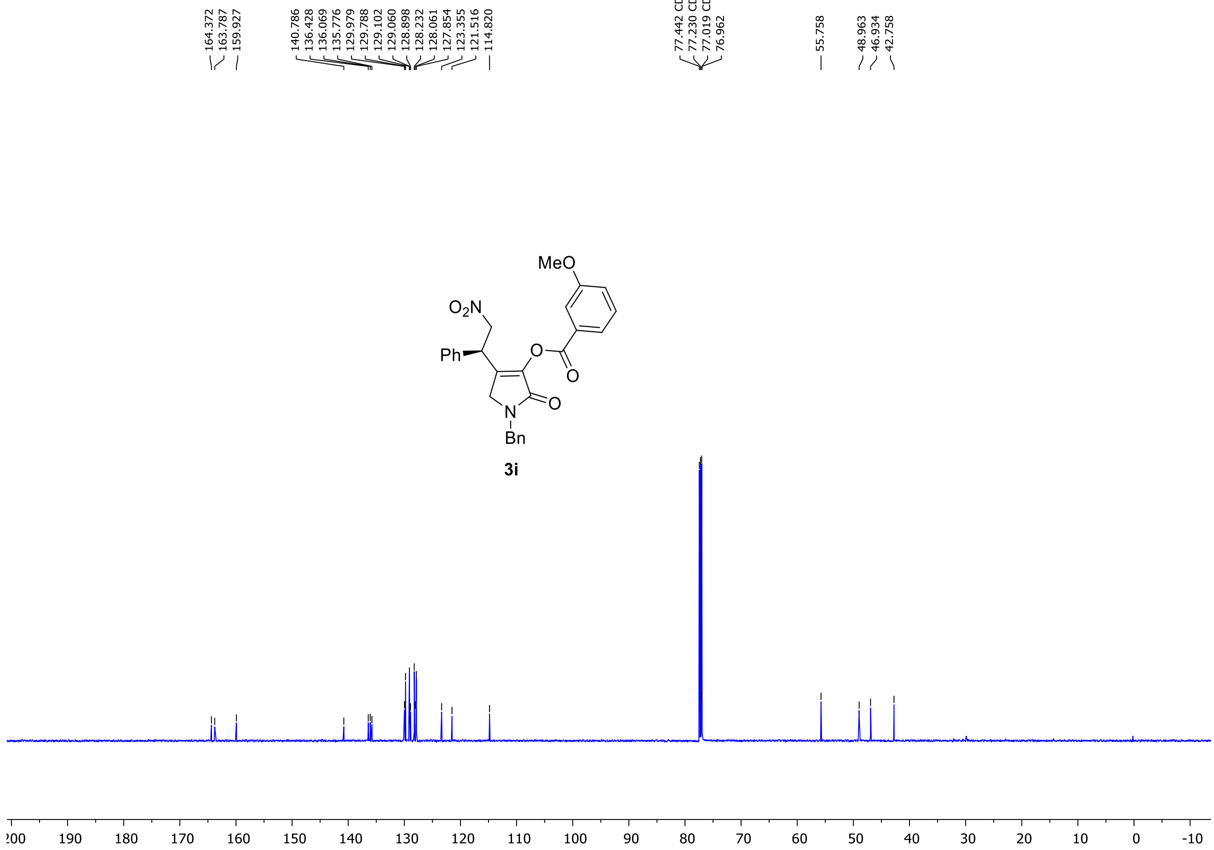
164.372
163.787
159.927
140.786
136.428
136.069
135.776
129.979
129.788
129.102
129.060
128.898
128.232
128.061
127.854
123.355
121.516
114.820
77.442 CDCl3
77.230 CDCl3
77.019 CDCl3
76.962
55.758
48.963
46.934
42.758
MeO
O2N
Ph
O
O
O
N
Bn
3i
200 190 180 170 160 150 140 130 120 110 100 90 80 70 60 50 40 30 20 10 0 -10

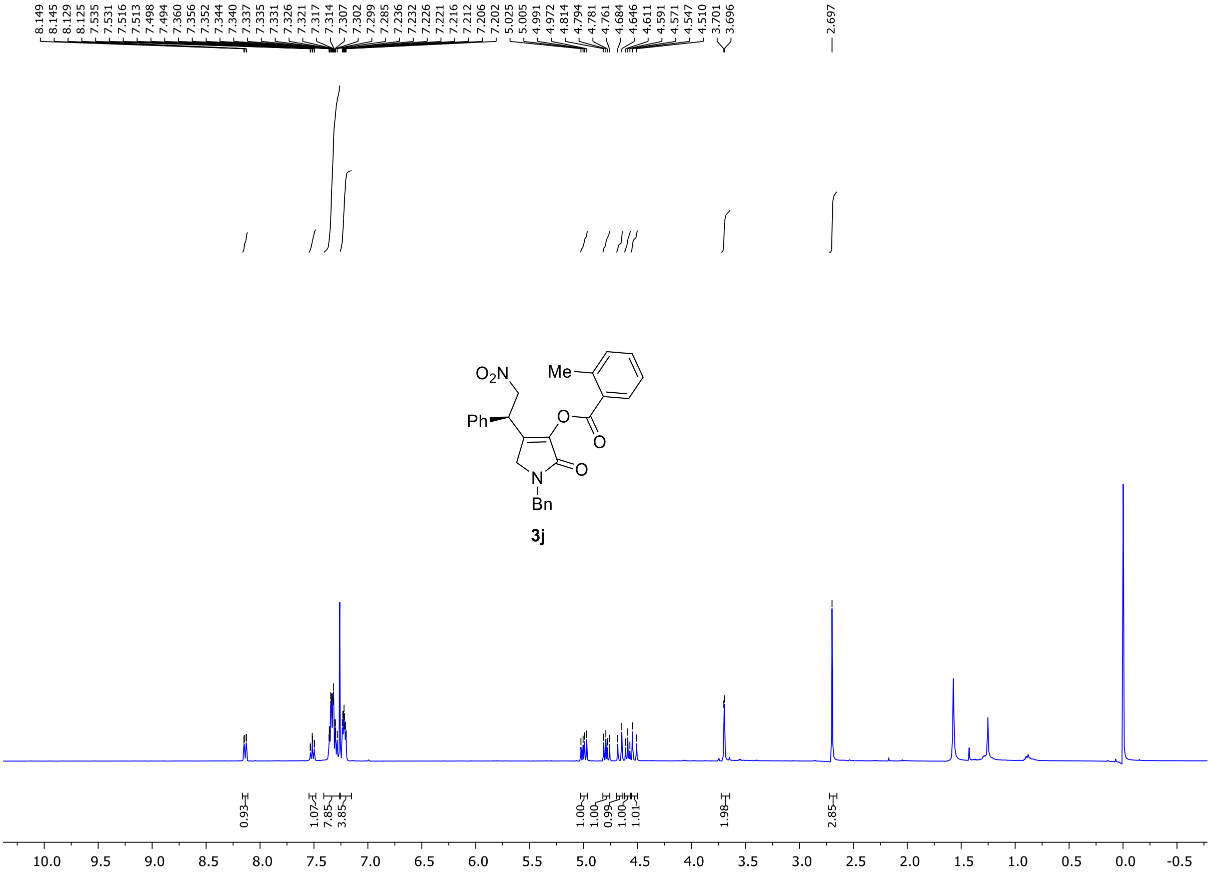

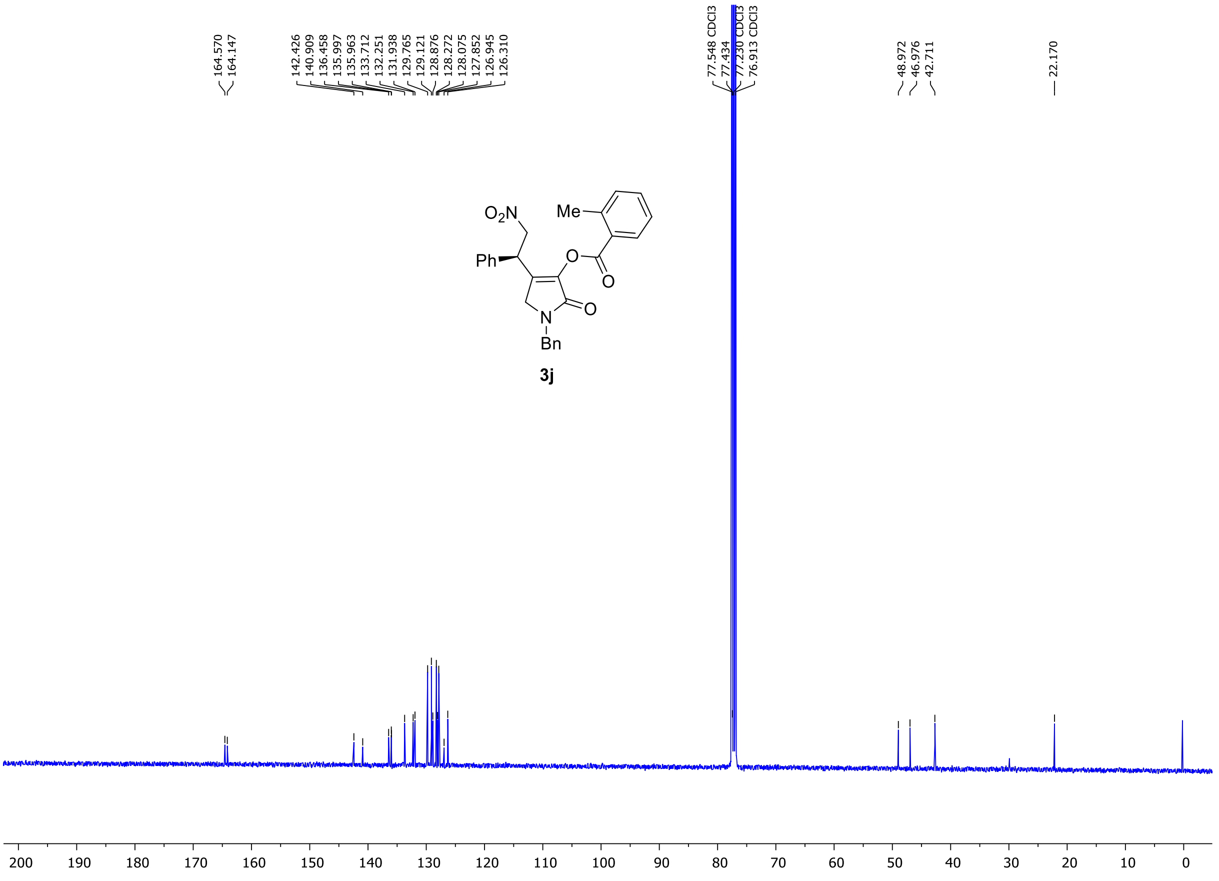

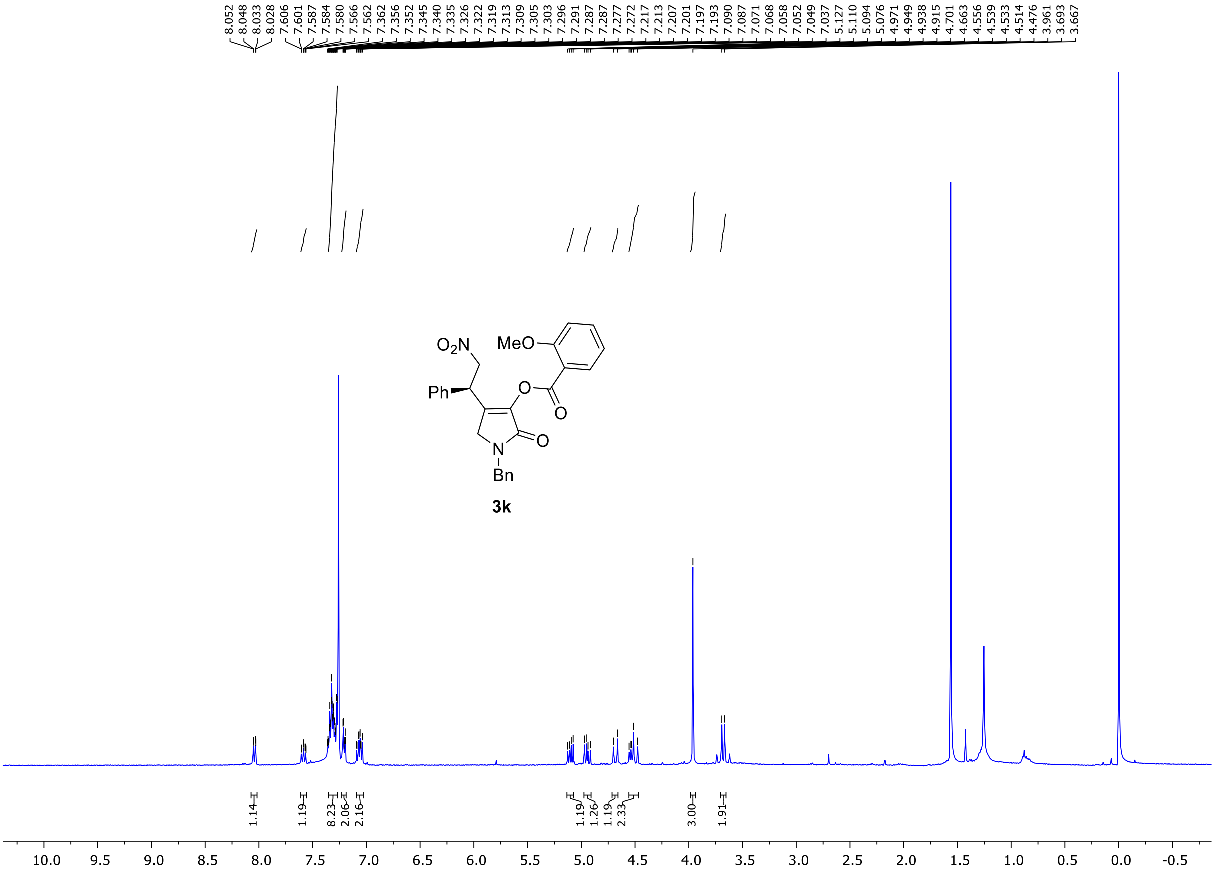

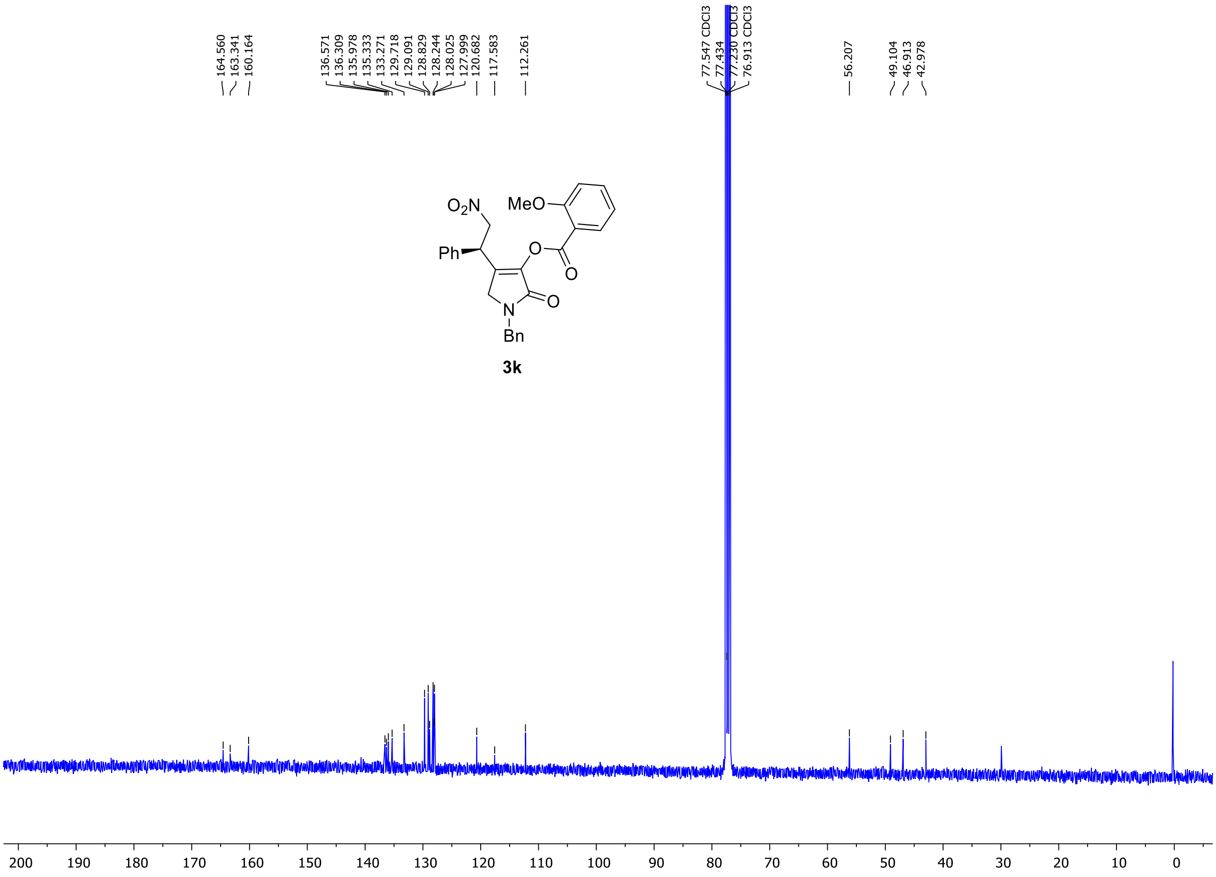

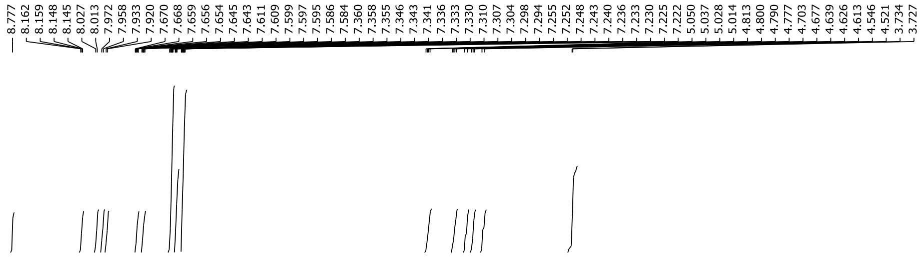
8.777 8.162 8.159 8.148 8.145 8.027 8.013 7.972 7.958 7.933 7.920 7.670 7.668 7.659 7.656 7.654 7.645 7.643 7.611 7.609 7.599 7.597 7.595 7.586 7.584 7.360 7.358 7.355 7.346 7.343 7.341 7.336 7.333 7.330 7.310 7.307 7.304 7.298 7.294 7.255 7.252 7.248 7.243 7.240 7.236 7.233 7.230 7.225 7.222 5.050 5.037 5.028 5.014 4.813 4.800 4.790 4.777 4.703 4.677 4.639 4.626 4.613 4.546 4.521 3.734 3.727

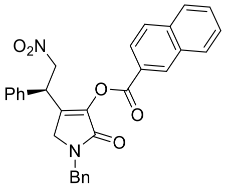

3l

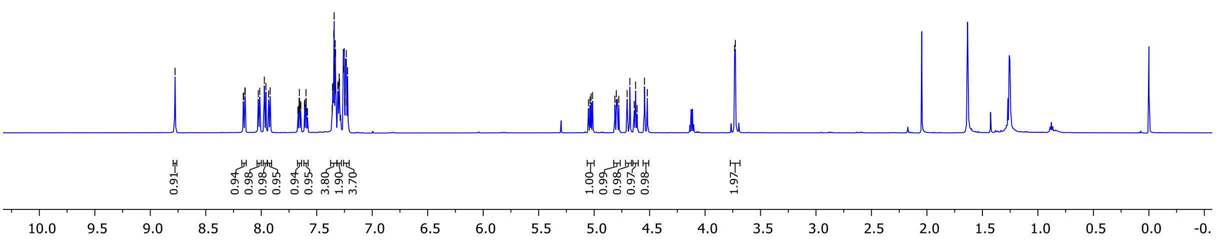
0.91 0.94 0.98 0.98 0.95 0.94 0.95 3.80 1.90 3.70 1.00 0.99 0.98 0.97 0.98 1.97

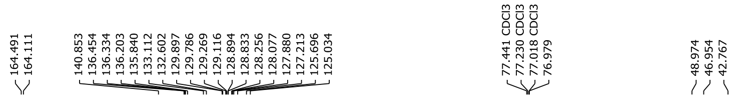
164.491 164.111 140.853 136.454 136.334 136.203 135.840 133.112 132.602 129.897 129.786 129.269 129.116 128.894 128.833 128.256 128.077 127.880 127.213 125.696 125.034 77.441 CDCl3 77.230 CDCl3 77.018 CDCl3 76.979 48.974 46.954 42.767

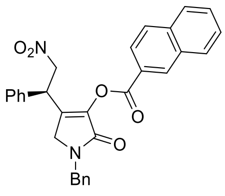

3l

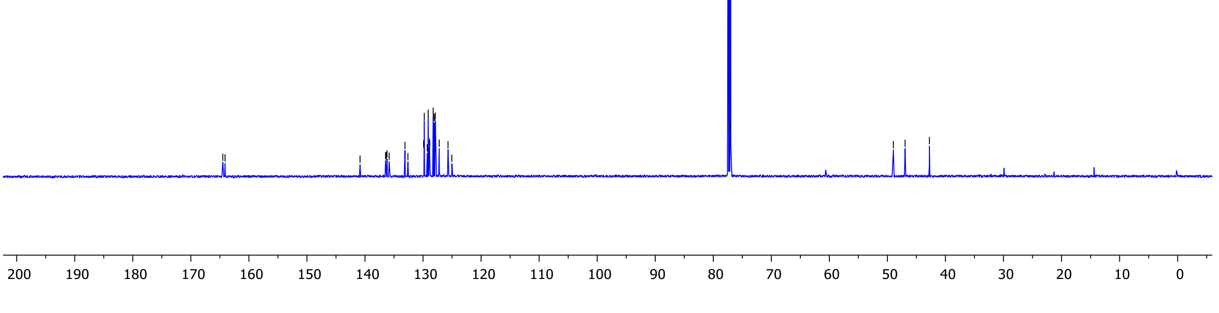

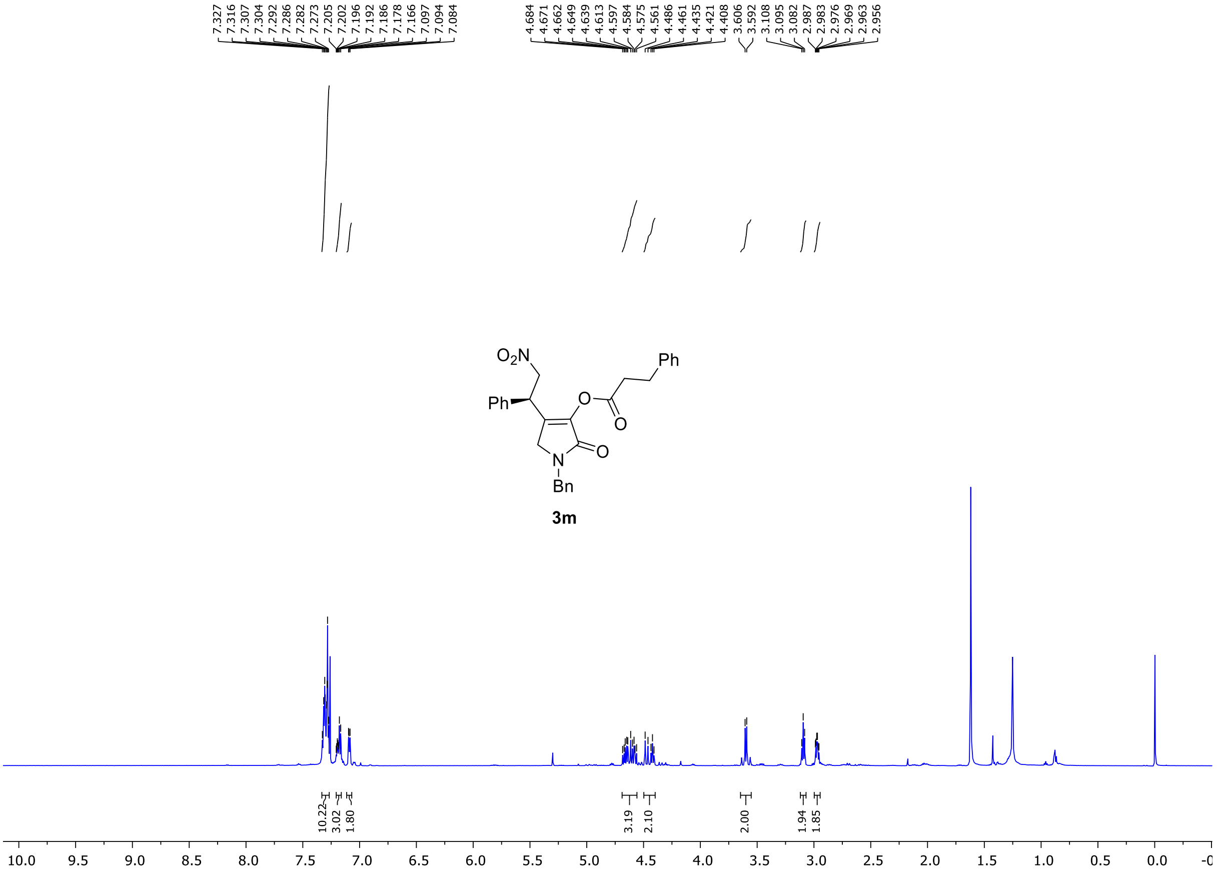
7.327 7.316 7.307 7.304 7.292 7.286 7.282 7.273 7.205 7.202 7.196 7.192 7.186 7.178 7.166 7.097 7.094 7.084
4.684 4.671 4.662 4.649 4.639 4.613 4.597 4.584 4.575 4.561 4.486 4.461 4.435 4.421 4.408 3.606 3.592 3.108 3.095 3.082 2.987 2.983 2.976 2.969 2.963 2.956
O2N
Ph
O
O
Ph
N
O
Bn
3m
10.22
3.02
1.80
3.19
2.10
2.00
1.94
1.85
10.0 9.5 9.0 8.5 8.0 7.5 7.0 6.5 6.0 5.5 5.0 4.5 4.0 3.5 3.0 2.5 2.0 1.5 1.0 0.5 0.0

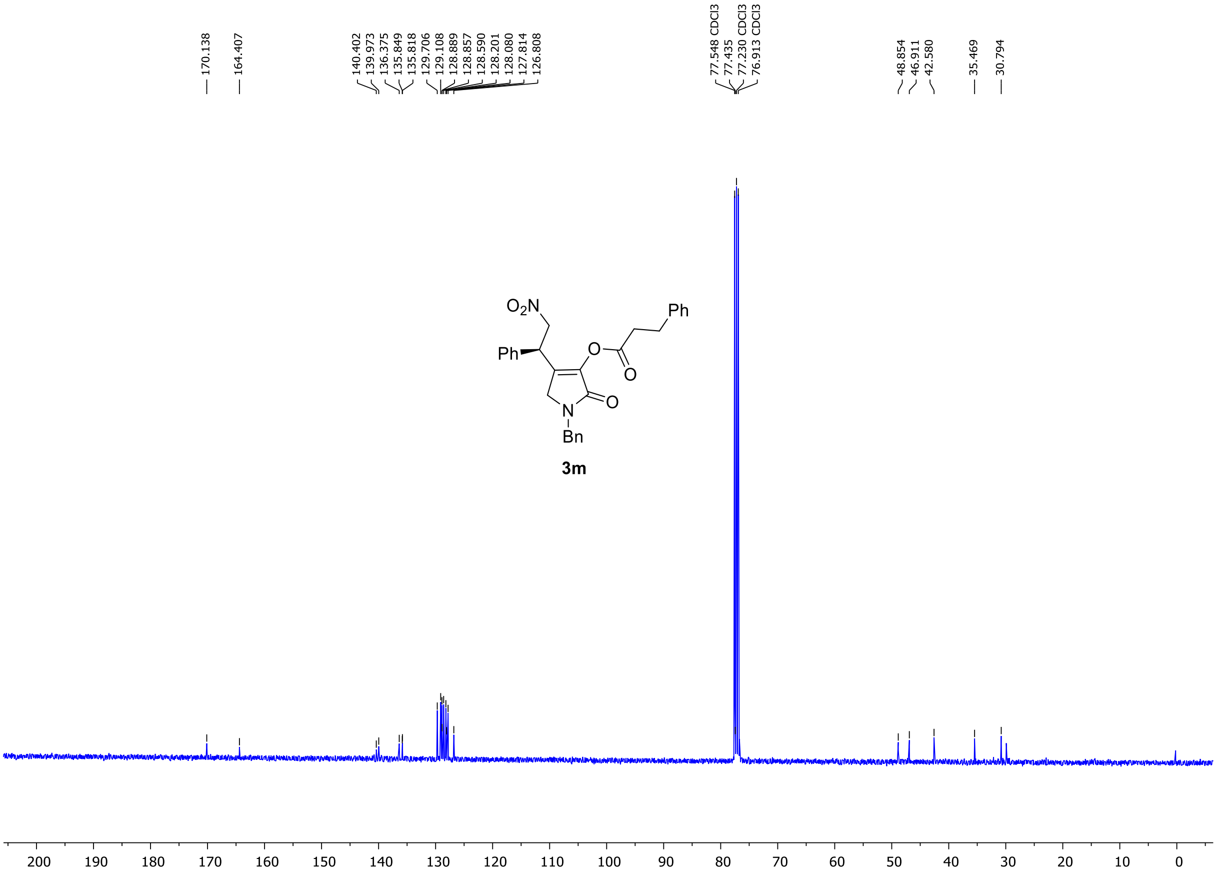
170.138
164.407
140.402 139.973 136.375 135.849 135.818 129.706 129.108 128.889 128.857 128.590 128.201 128.080 127.814 126.808
77.548 CDCl3
77.435 CDCl3
77.230 CDCl3
76.913 CDCl3
48.854 46.911 42.580
35.469
30.794
O2N
Ph
O
O
Ph
N
O
Bn
3m
200 190 180 170 160 150 140 130 120 110 100 90 80 70 60 50 40 30 20 10 0

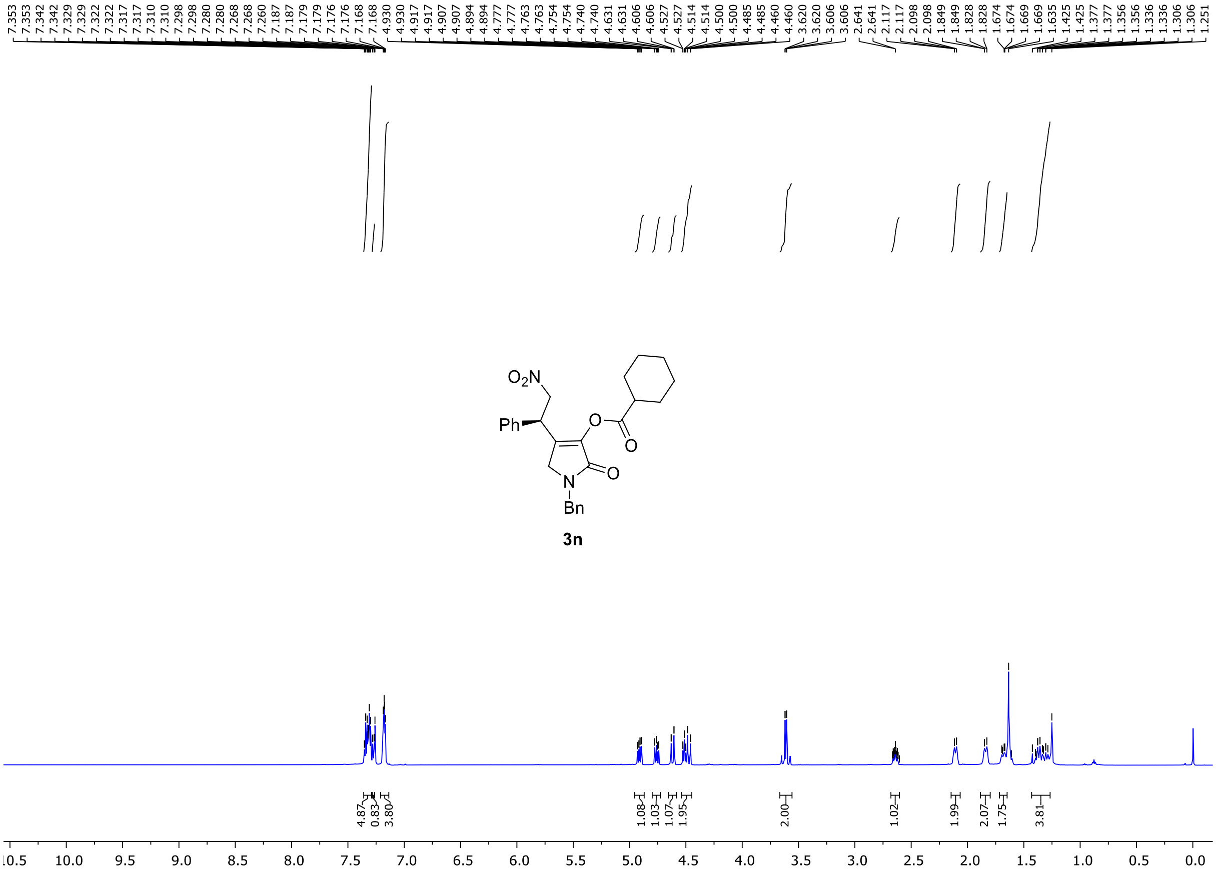
3n

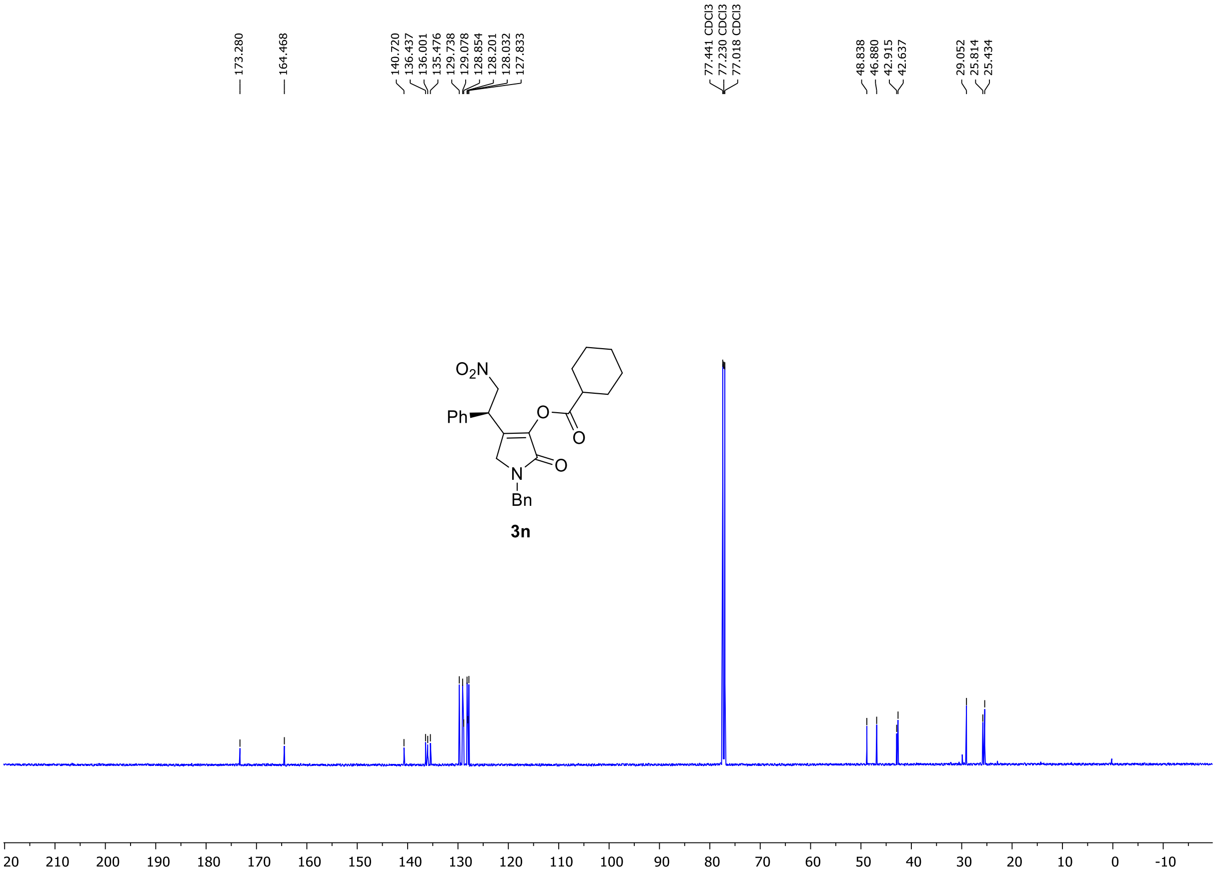
3n

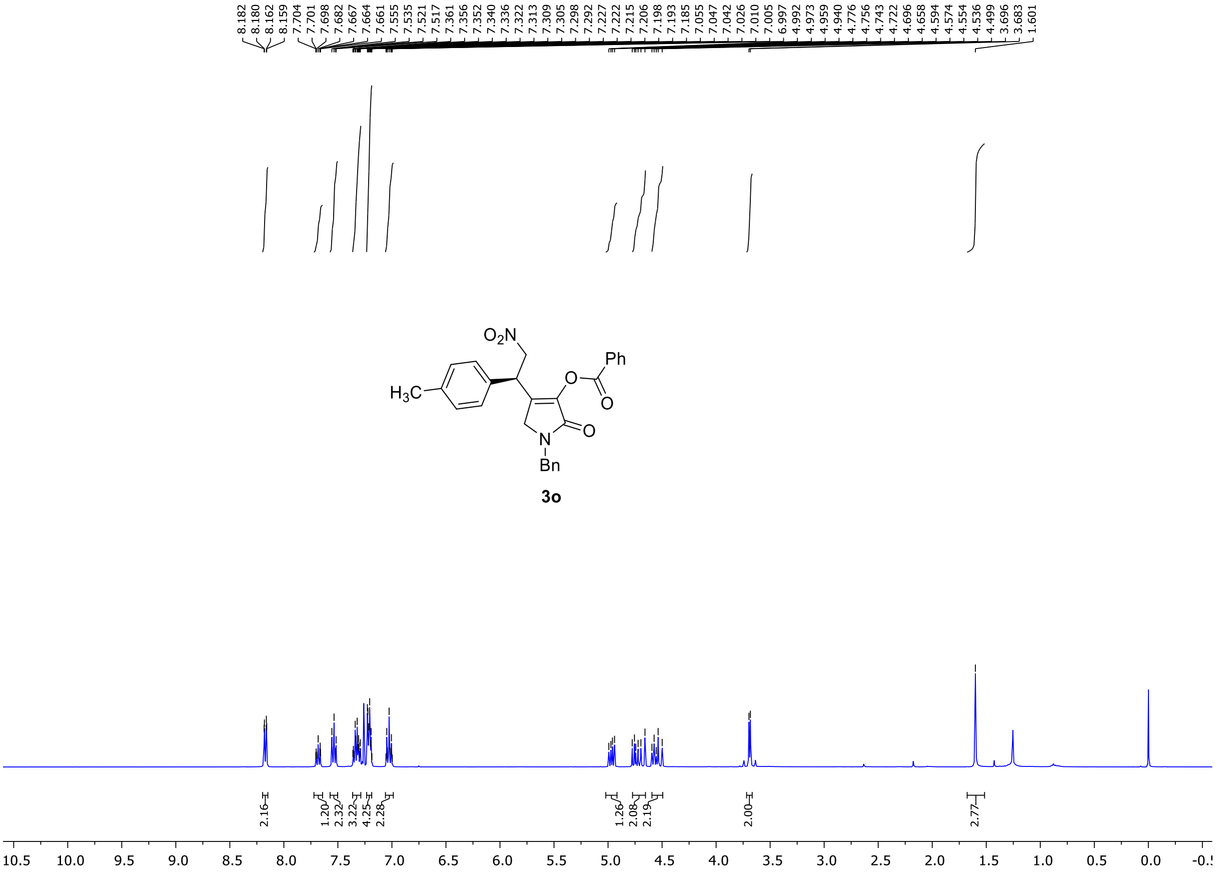

8.182
8.180
8.162
8.159
7.704
7.701
7.698
7.682
7.667
7.664
7.661
7.555
7.535
7.521
7.517
7.361
7.356
7.352
7.340
7.336
7.322
7.313
7.309
7.305
7.298
7.292
7.227
7.222
7.215
7.206
7.198
7.193
7.185
7.055
7.047
7.042
7.026
7.010
7.005
6.997
4.992
4.973
4.959
4.940
4.776
4.756
4.743
4.722
4.696
4.658
4.594
4.574
4.554
4.536
4.499
3.696
3.683
1.601
O_2N
Ph
H_3C
O
O
O
N
Bn
3o
2.16
1.20
2.32
3.22
4.25
2.28
1.26
2.08
2.19
2.00
2.77
10.5 10.0 9.5 9.0 8.5 8.0 7.5 7.0 6.5 6.0 5.5 5.0 4.5 4.0 3.5 3.0 2.5 2.0 1.5 1.0 0.5 0.0

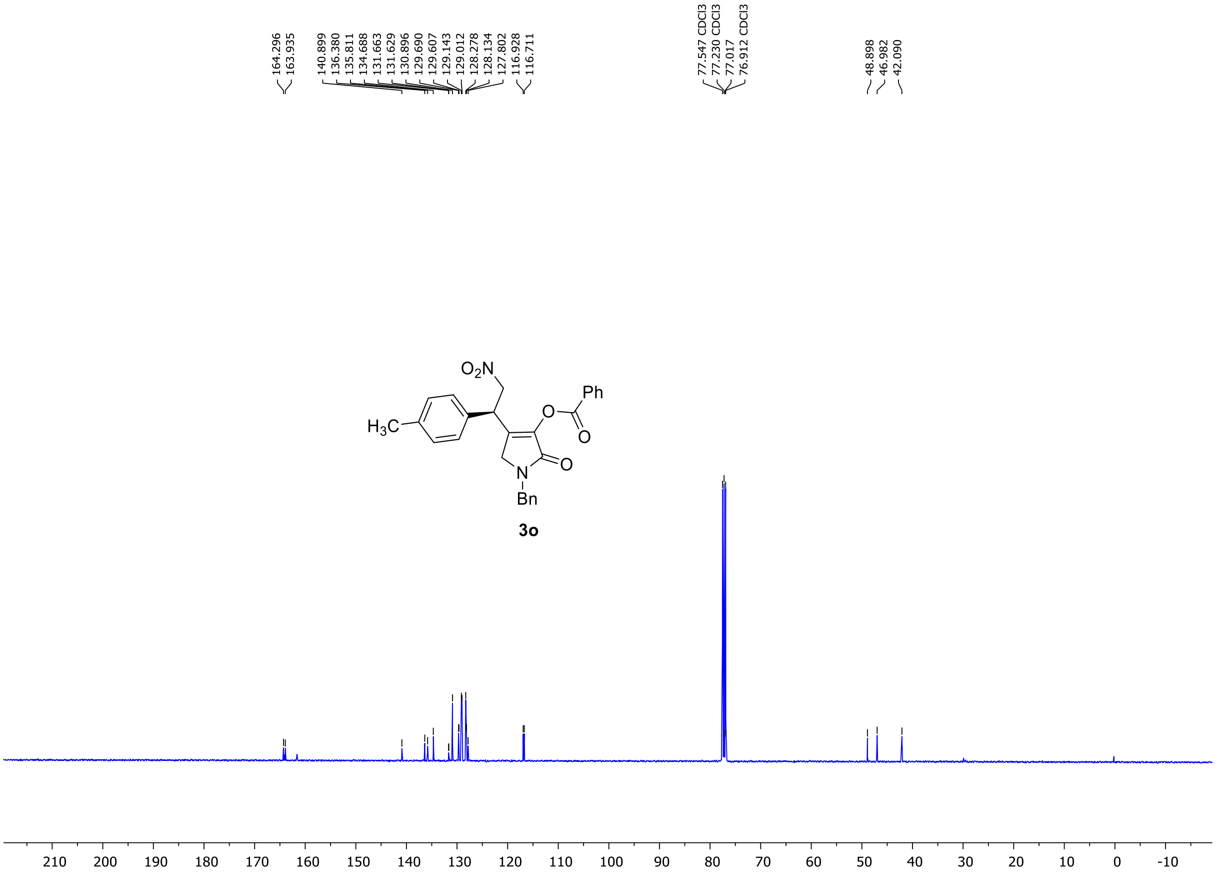

164.296
163.935
140.899
136.380
135.811
134.688
131.663
131.629
130.896
129.690
129.607
129.143
129.012
128.278
128.134
127.802
116.928
116.711
77.547 CDCl3
77.230 CDCl3
77.017
76.912 CDCl3
48.898
46.982
42.090
O_2N
Ph
H_3C
O
O
O
N
Bn
3o
210 200 190 180 170 160 150 140 130 120 110 100 90 80 70 60 50 40 30 20 10 0 -10

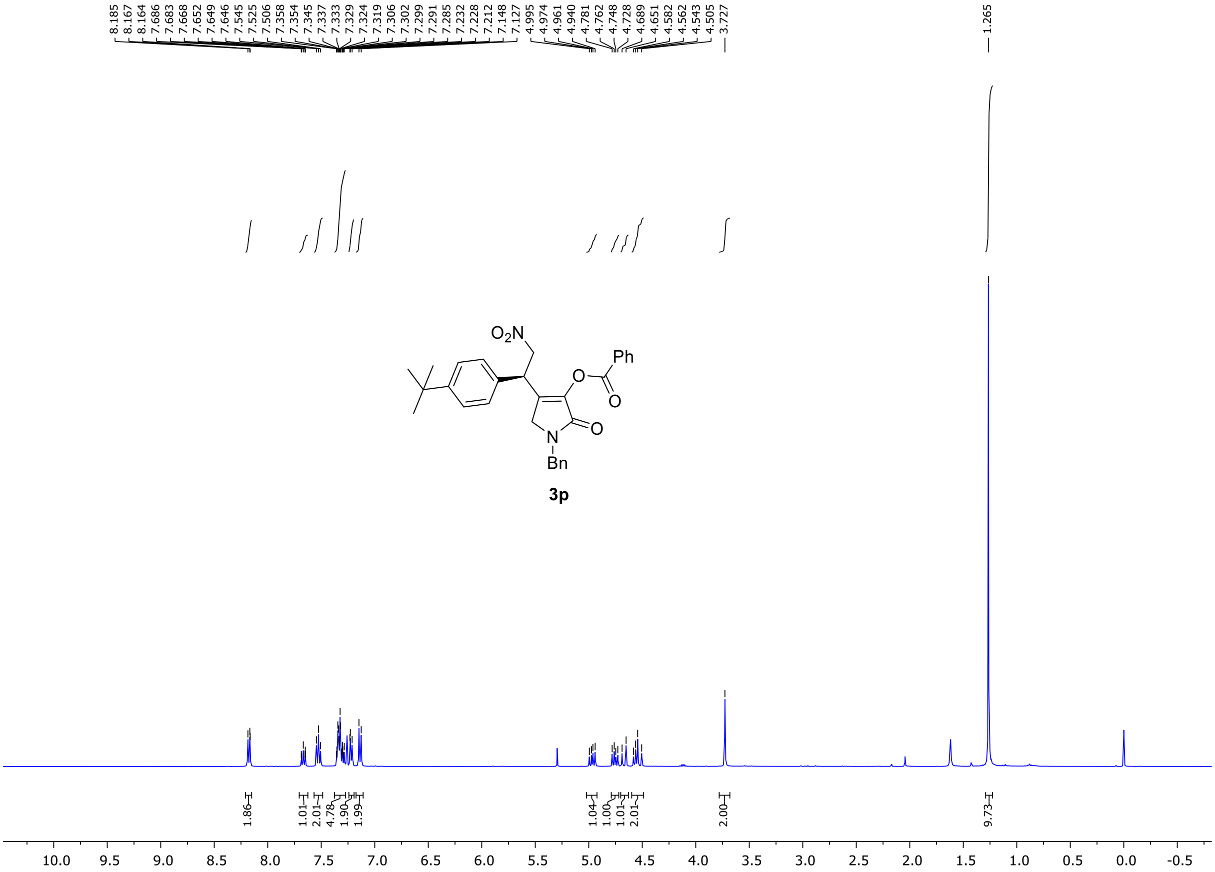

3p

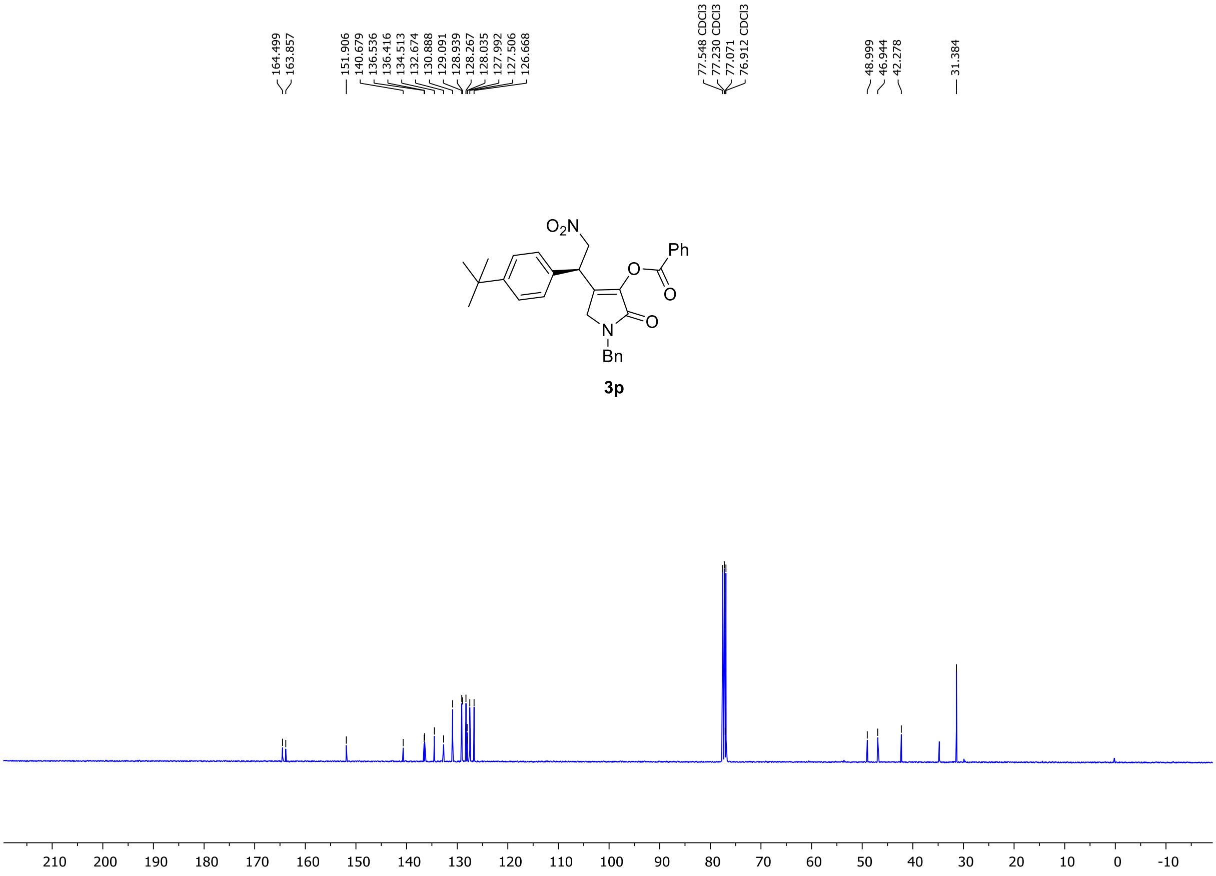

3p

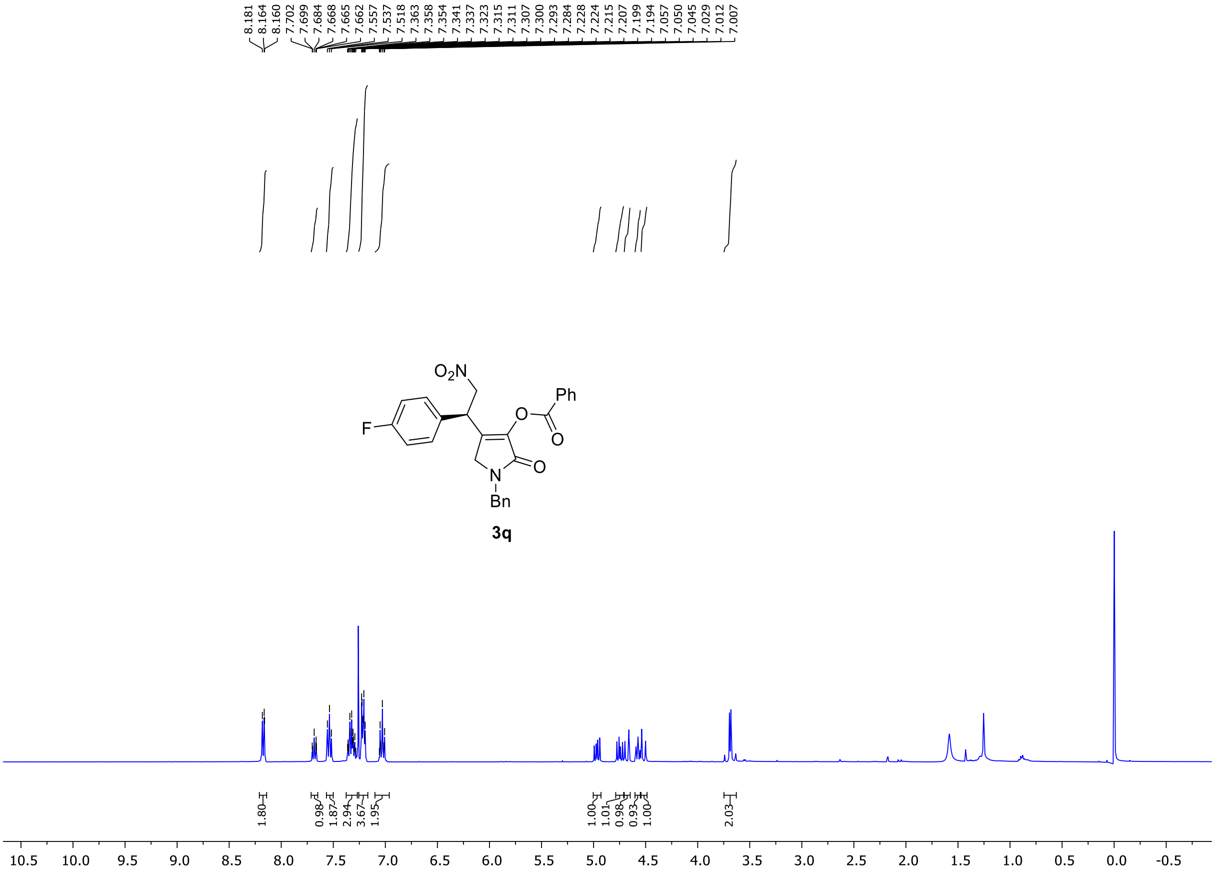

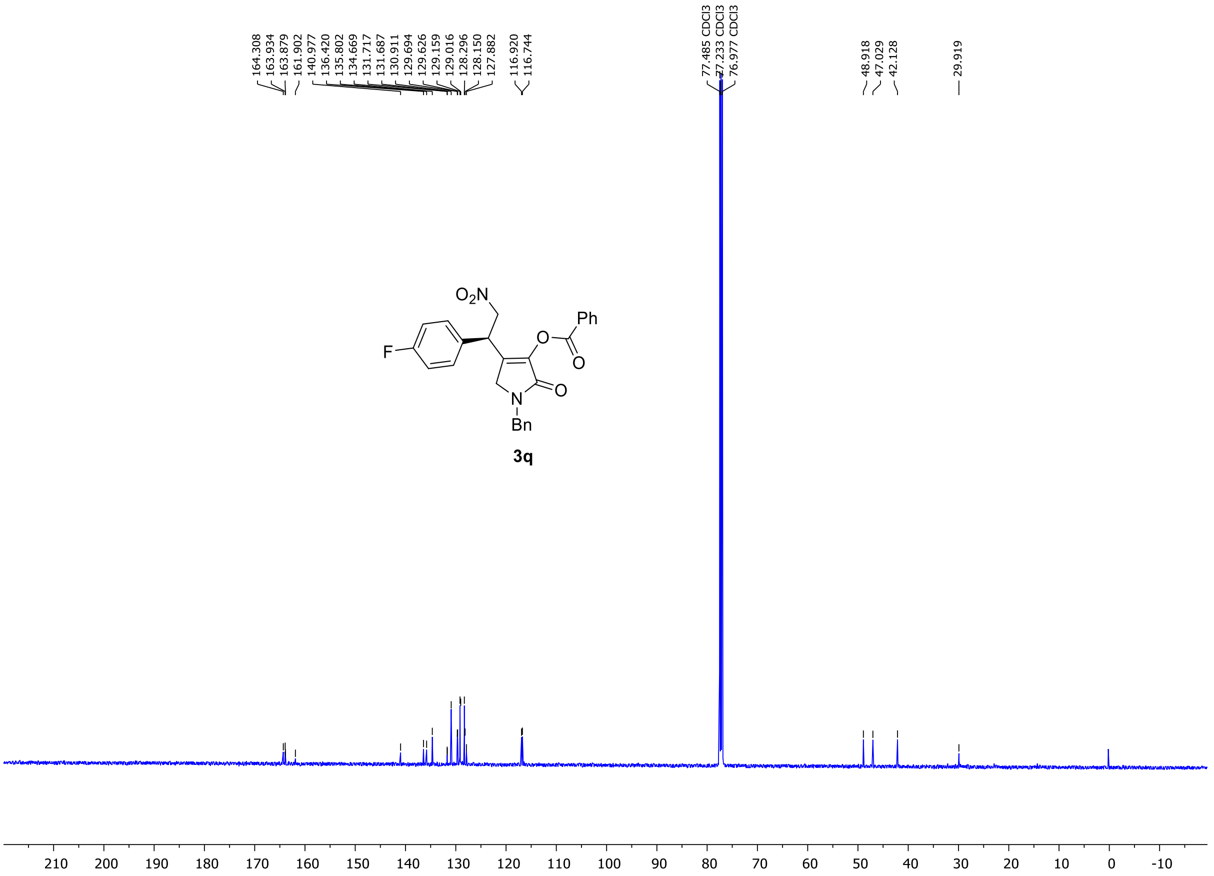

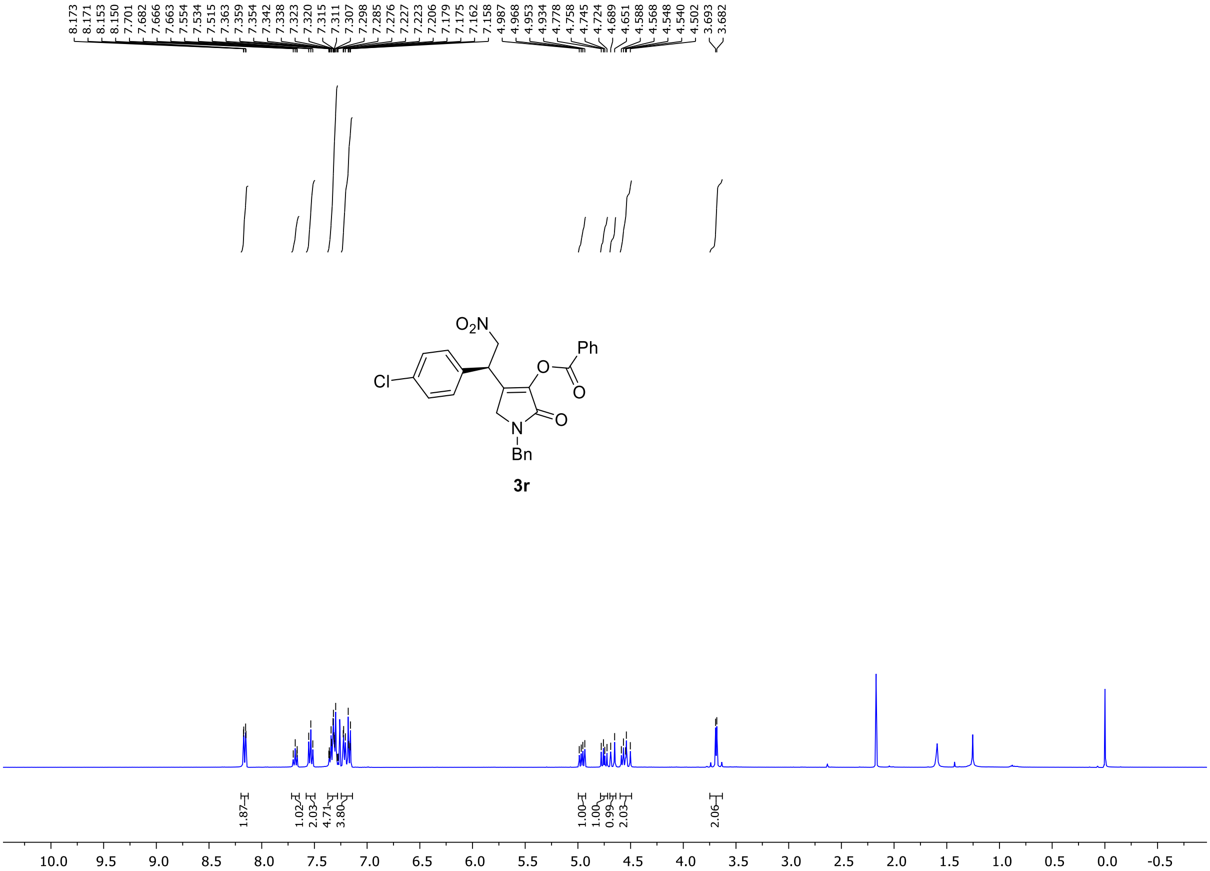
8.173 8.171 8.153 8.150 7.701 7.682 7.666 7.663 7.554 7.534 7.515 7.363 7.359 7.354 7.342 7.338 7.323 7.320 7.315 7.311 7.307 7.298 7.285 7.276 7.227 7.223 7.206 7.179 7.175 7.162 7.158 4.987 4.968 4.953 4.934 4.778 4.758 4.745 4.724 4.689 4.651 4.588 4.568 4.548 4.540 4.502 3.693 3.682
O_2N
Ph
Cl
O
O
N
O
Bn
3r
1.87
1.02
2.03
4.71
3.80
1.00
1.00
0.99
2.03
2.06
10.0 9.5 9.0 8.5 8.0 7.5 7.0 6.5 6.0 5.5 5.0 4.5 4.0 3.5 3.0 2.5 2.0 1.5 1.0 0.5 0.0 -0.5

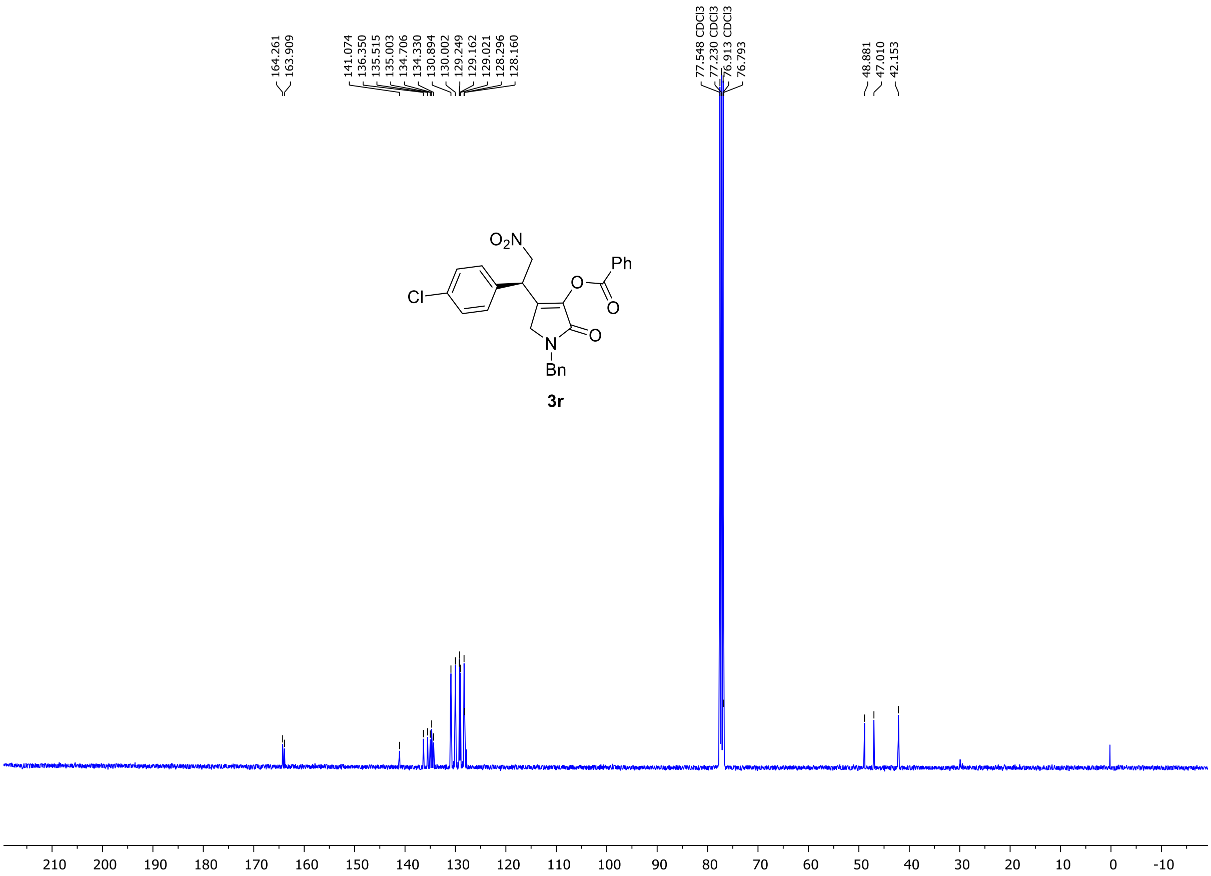
164.261 163.909
141.074 136.350 135.515 135.003 134.706 134.330 130.894 130.002 129.249 129.162 129.021 128.296 128.160
77.548 CDCl3 77.230 CDCl3 76.913 CDCl3 76.793
48.881 47.010 42.153
O_2N
Ph
Cl
O
O
N
O
Bn
3r
210 200 190 180 170 160 150 140 130 120 110 100 90 80 70 60 50 40 30 20 10 0 -10

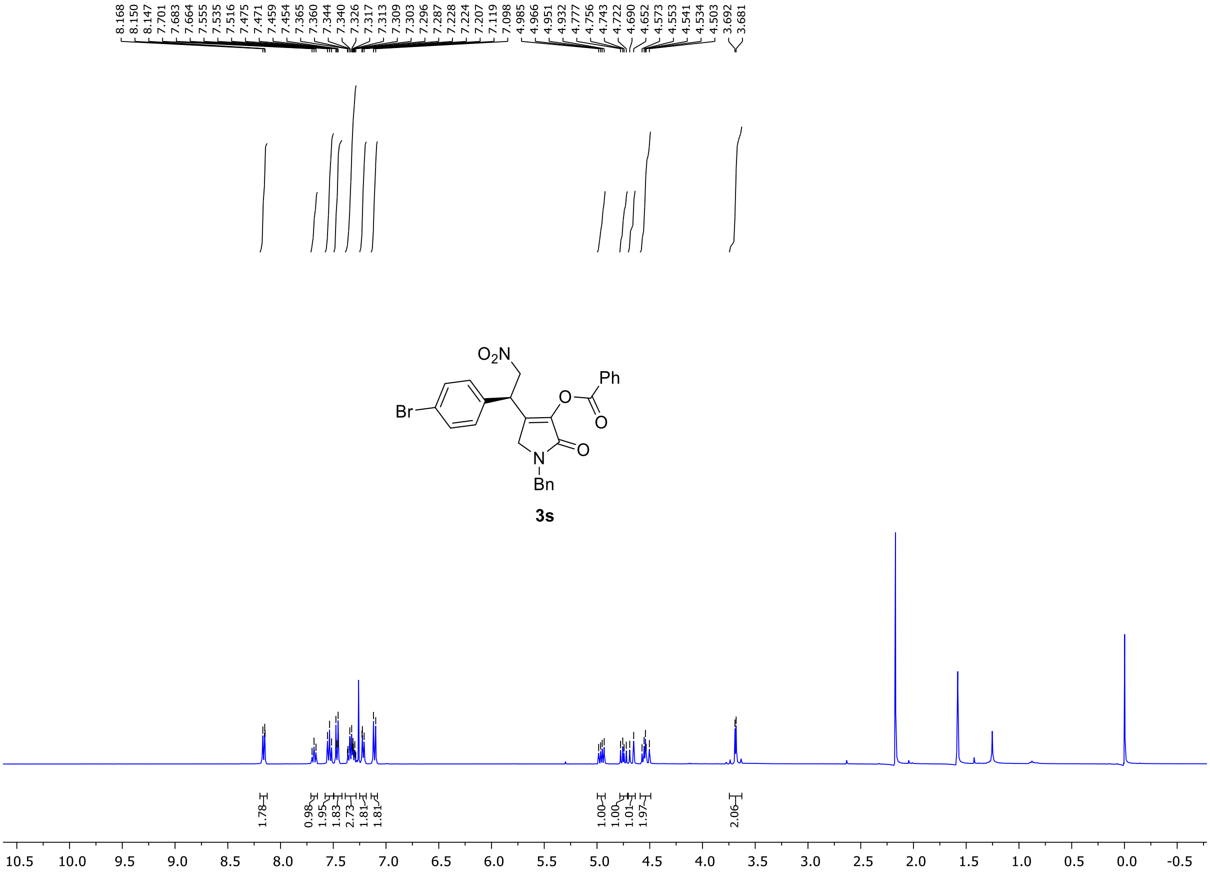
8.168
8.150
8.147
7.701
7.683
7.664
7.555
7.535
7.516
7.475
7.471
7.459
7.454
7.365
7.360
7.344
7.340
7.326
7.317
7.313
7.309
7.303
7.296
7.287
7.228
7.224
7.207
7.119
7.098
4.985
4.966
4.951
4.932
4.777
4.756
4.743
4.722
4.690
4.652
4.573
4.553
4.541
4.534
4.503
3.692
3.681
O2N
Ph
Br
O
N
Bn
3s
1.78
0.98
1.95
1.83
2.73
1.81
1.81
1.00
1.00
1.01
1.97
2.06
10.5 10.0 9.5 9.0 8.5 8.0 7.5 7.0 6.5 6.0 5.5 5.0 4.5 4.0 3.5 3.0 2.5 2.0 1.5 1.0 0.5 0.0 -0.5

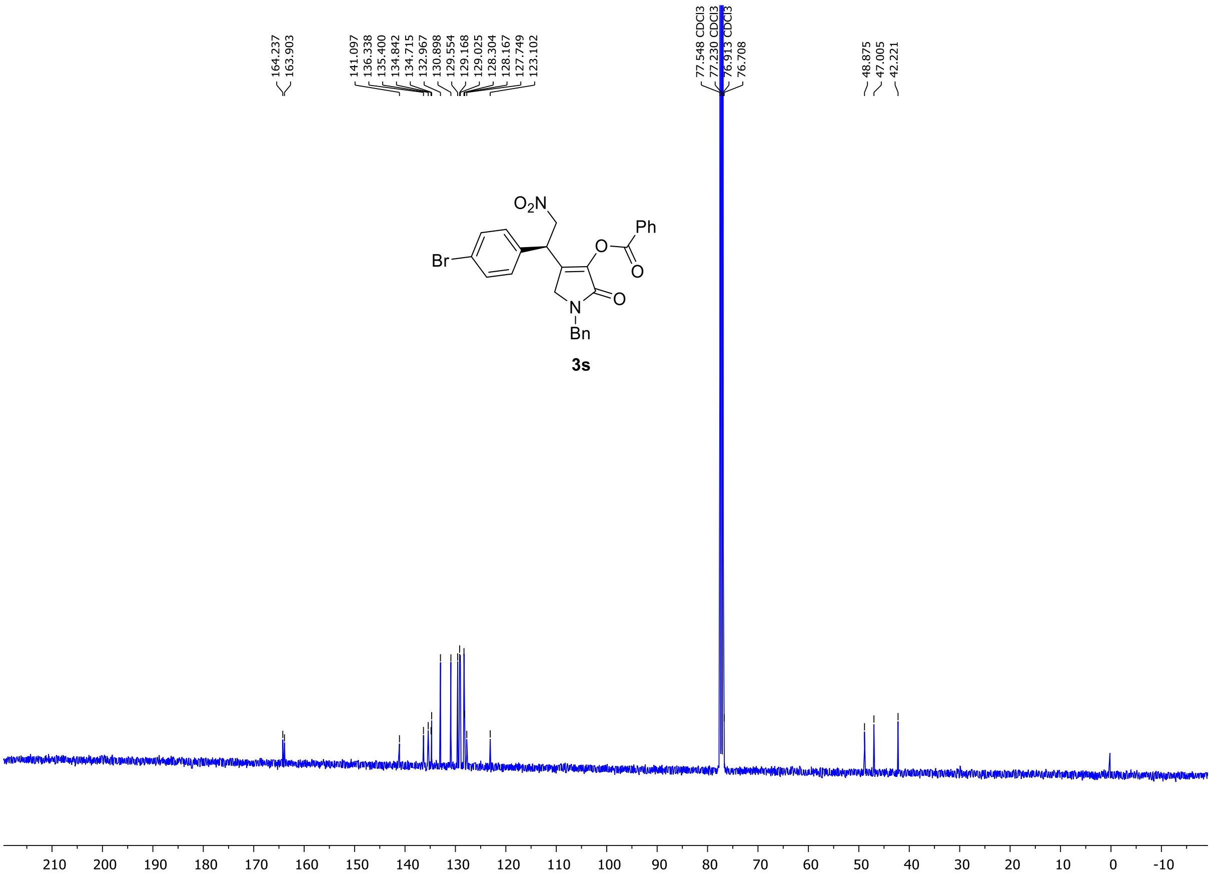
164.237
163.903
141.097
136.338
135.400
134.842
134.715
132.967
130.898
129.554
129.168
129.025
128.304
128.167
127.749
123.102
77.548 CDCl3
77.230 CDCl3
76.913 CDCl3
76.708
48.875
47.005
42.221
O2N
Ph
Br
O
N
Bn
3s
210 200 190 180 170 160 150 140 130 120 110 100 90 80 70 60 50 40 30 20 10 0 -10

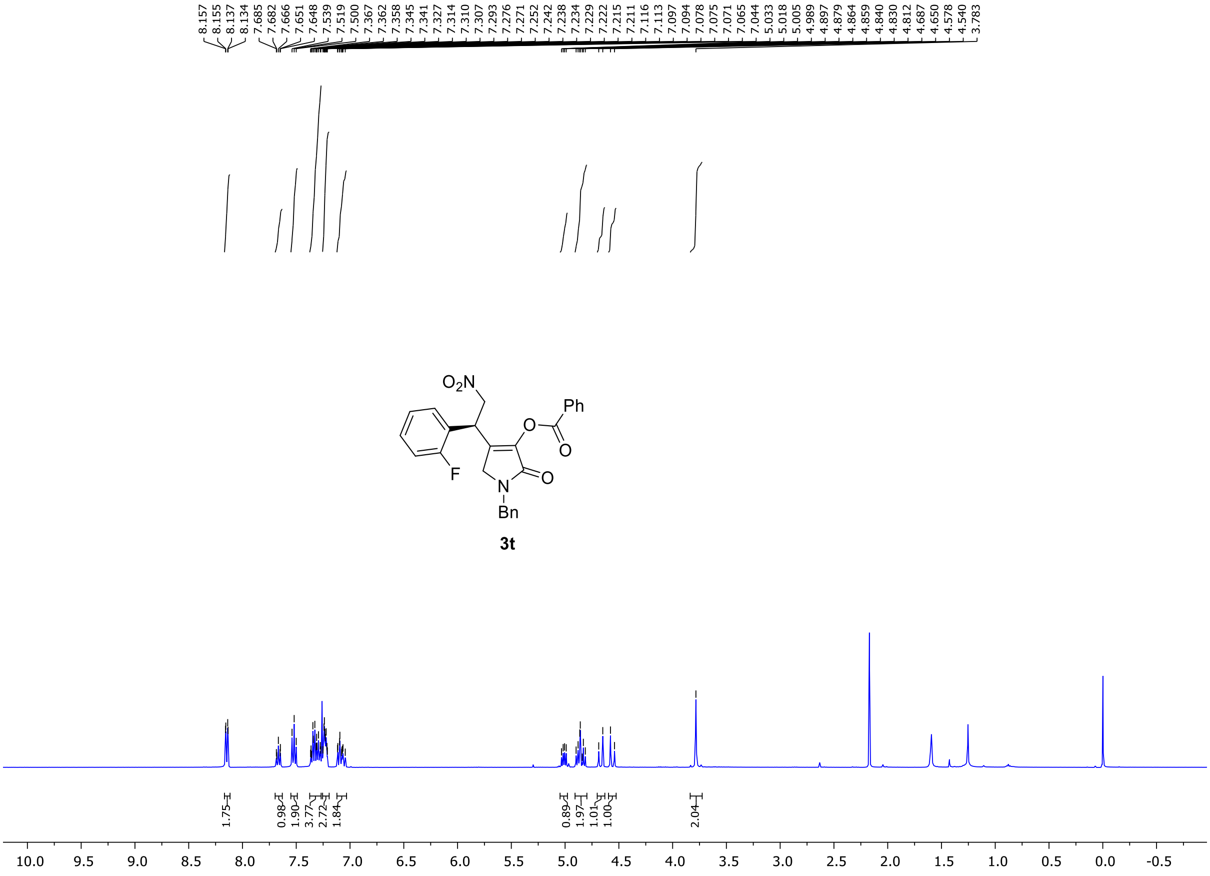
O2N
Ph
O
O
F
N
Bn
3t

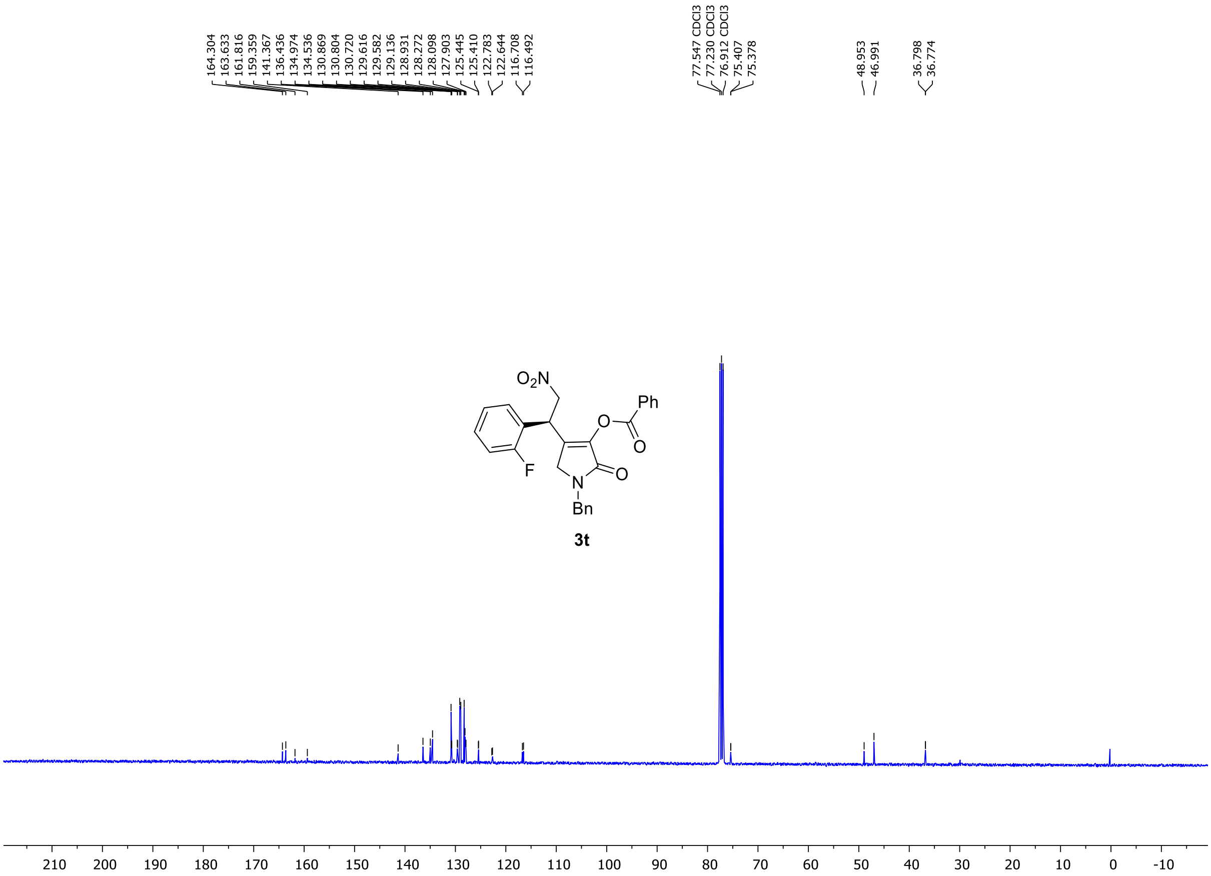
O2N
Ph
O
O
F
N
Bn
3t

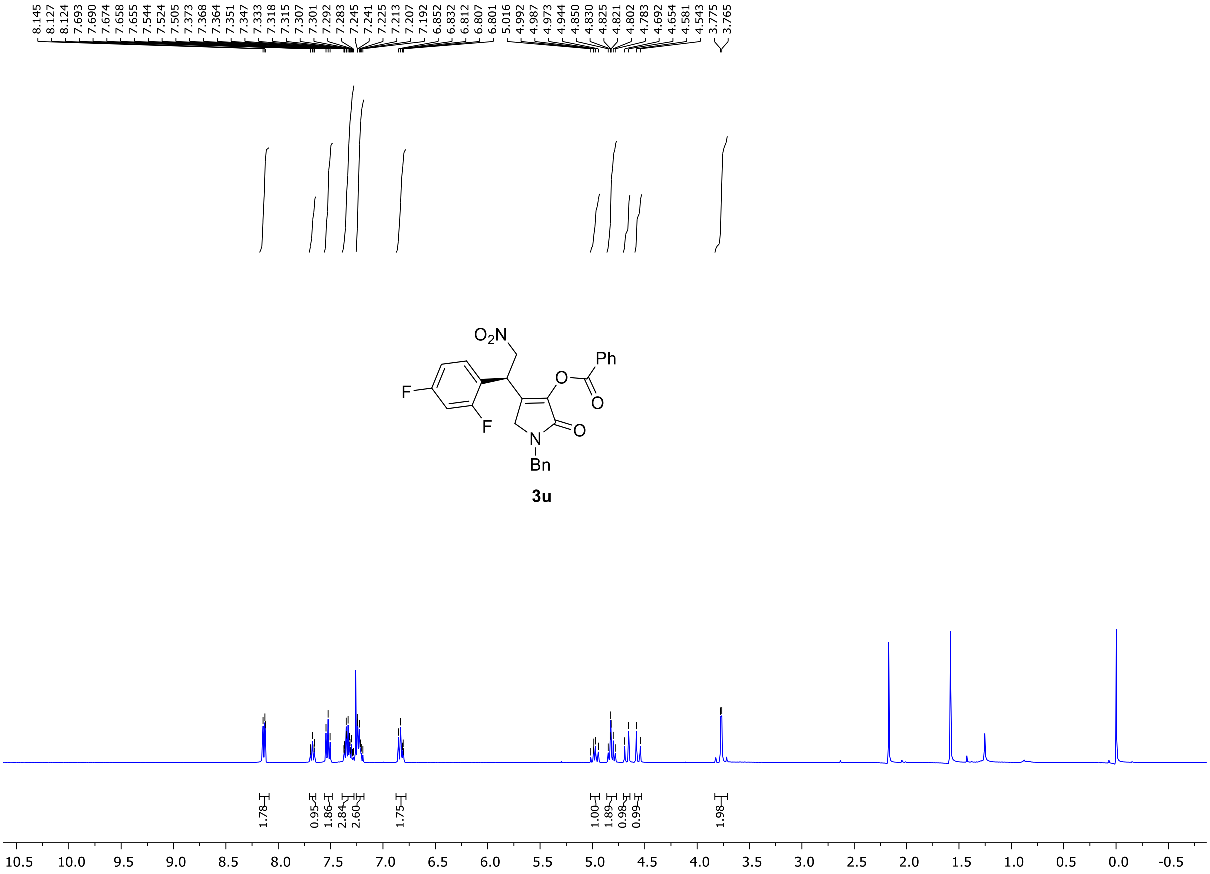
8.145
8.127
8.124
7.693
7.690
7.674
7.658
7.655
7.544
7.524
7.505
7.373
7.368
7.364
7.351
7.347
7.333
7.318
7.315
7.307
7.301
7.292
7.283
7.245
7.241
7.225
7.213
7.207
7.192
6.852
6.832
6.812
6.807
6.801
5.016
4.992
4.987
4.973
4.944
4.850
4.830
4.825
4.821
4.802
4.783
4.692
4.654
4.581
4.543
3.775
3.765
O_2N
Ph
F
F
O
O
O
N
Bn
3u
1.78
0.95
1.86
2.84
2.60
1.75
1.00
1.89
0.98
0.99
1.98
10.5
10.0
9.5
9.0
8.5
8.0
7.5
7.0
6.5
6.0
5.5
5.0
4.5
4.0
3.5
3.0
2.5
2.0
1.5
1.0
0.5
0.0
-0.5

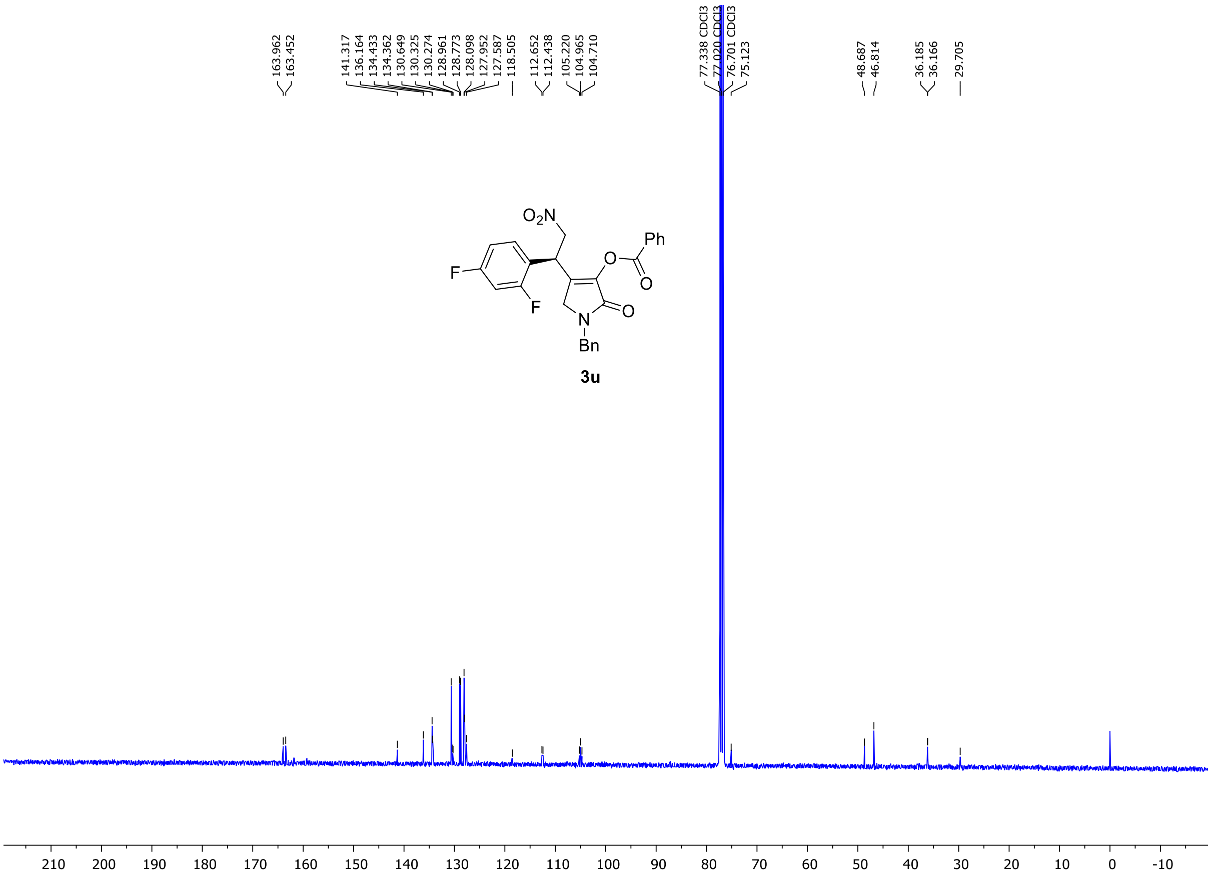
163.962
163.452
141.317
136.164
134.433
134.362
130.649
130.325
130.274
128.961
128.773
128.098
127.952
127.587
118.505
112.652
112.438
105.220
104.965
104.710
77.338 CDCl3
77.020 CDCl3
76.701 CDCl3
75.123
48.687
46.814
36.185
36.166
29.705
O_2N
Ph
F
F
O
O
O
N
Bn
3u
210
200
190
180
170
160
150
140
130
120
110
100
90
80
70
60
50
40
30
20
10
0
-10

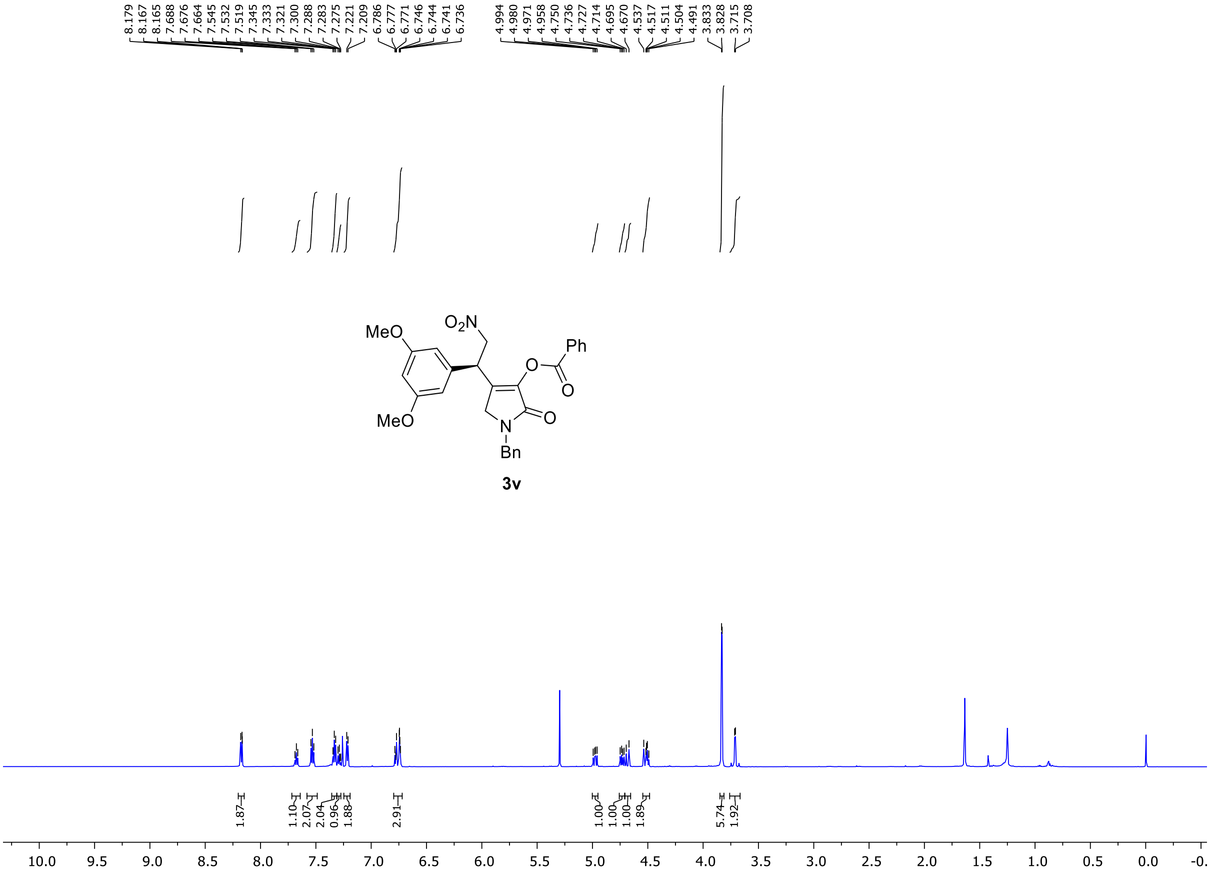

MeO
O_2N
Ph
O
O
O
MeO
N
Bn
3v
8.179 8.167 8.165 7.688 7.676 7.664 7.545 7.532 7.519 7.345 7.333 7.321 7.300 7.288 7.283 7.275 7.221 7.209 6.786 6.777 6.771 6.746 6.744 6.741 6.736
4.994 4.980 4.971 4.958 4.750 4.736 4.727 4.714 4.695 4.670 4.537 4.517 4.511 4.504 4.491 3.833 3.828 3.715 3.708
1.87 1.10 2.07 2.04 0.96 1.88 2.91 1.00 1.00 1.00 1.89 5.74 1.92
10.0 9.5 9.0 8.5 8.0 7.5 7.0 6.5 6.0 5.5 5.0 4.5 4.0 3.5 3.0 2.5 2.0 1.5 1.0 0.5 0.0

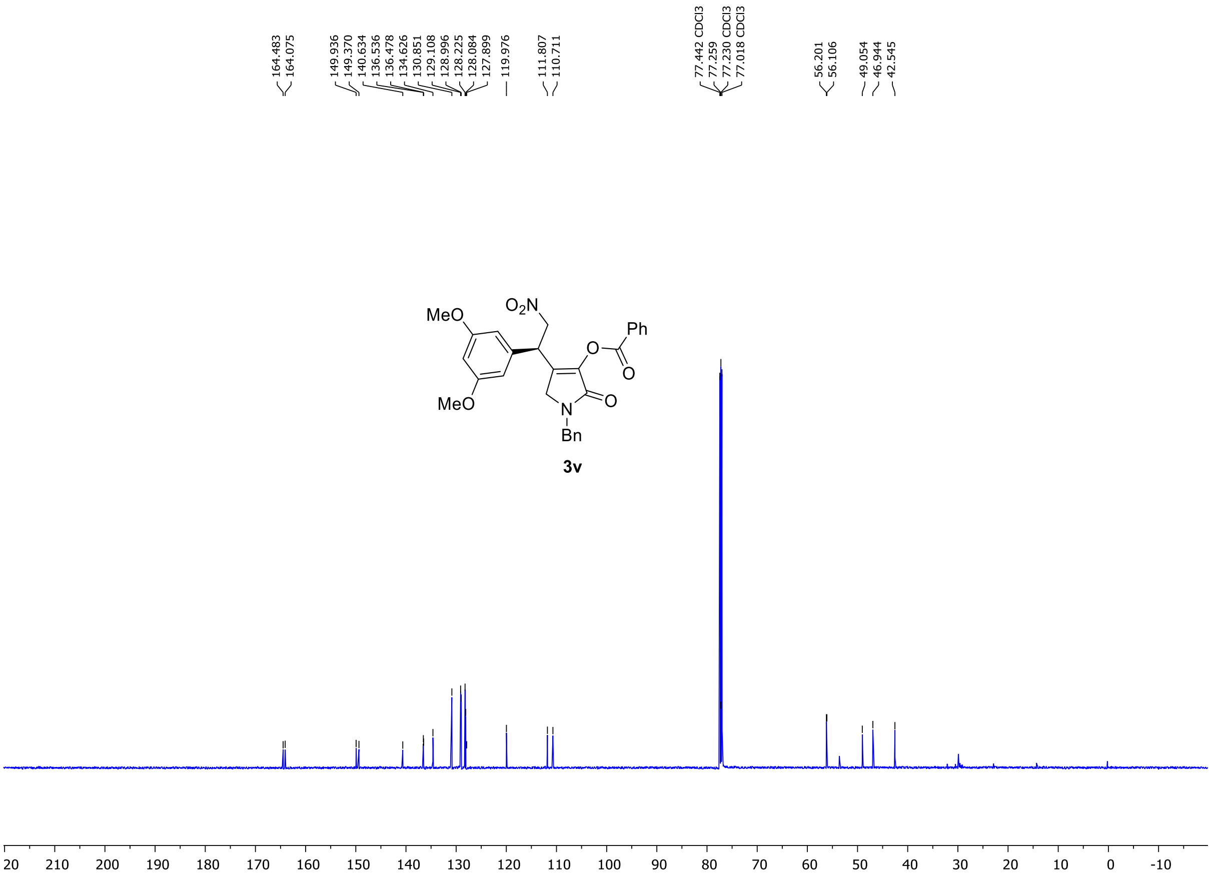

164.483 164.075 149.936 149.370 140.634 136.536 136.478 134.626 130.851 129.108 128.996 128.225 128.084 127.899 119.976 111.807 110.711
77.442 CDCl3 77.259 77.230 CDCl3 77.018 CDCl3
56.201 56.106 49.054 46.944 42.545
MeO
O_2N
Ph
O
O
O
MeO
N
Bn
3v
210 200 190 180 170 160 150 140 130 120 110 100 90 80 70 60 50 40 30 20 10 0 -10

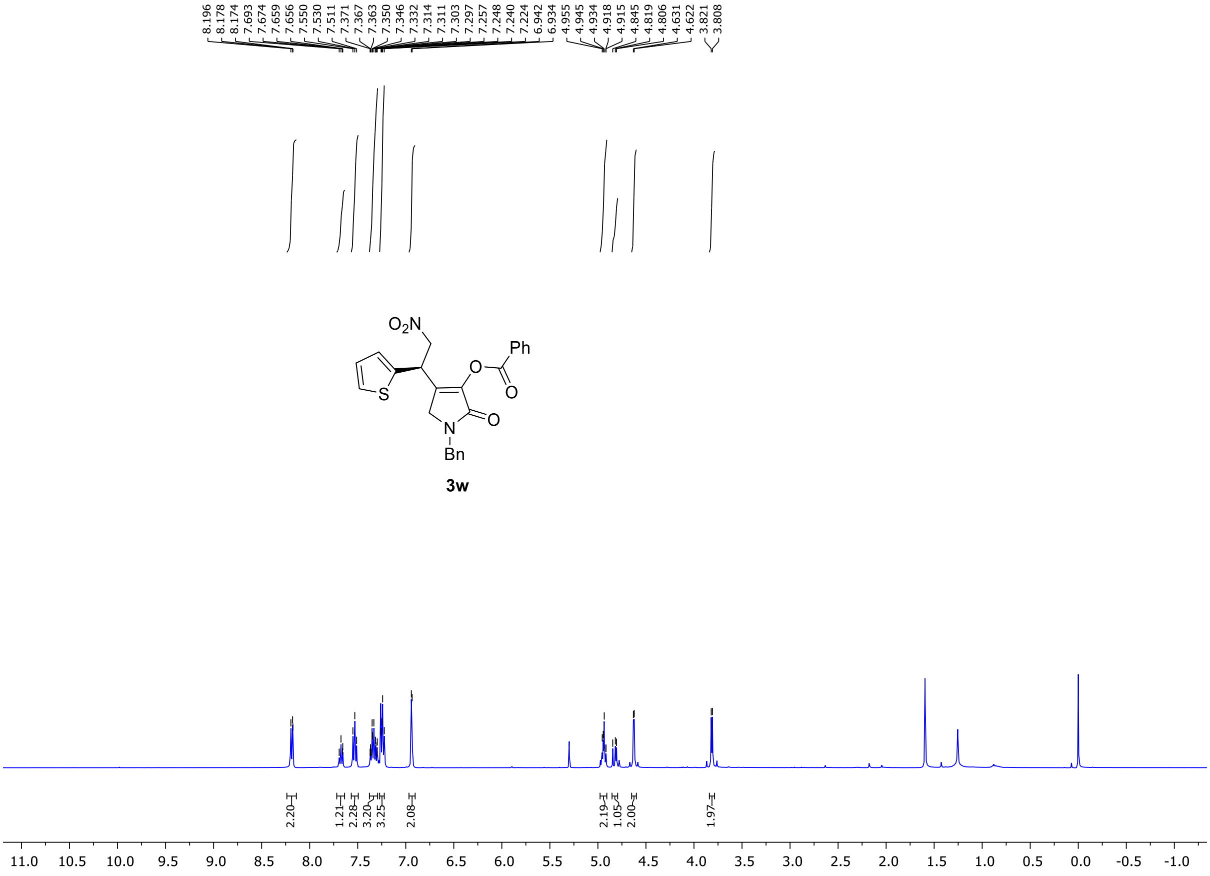
8.196
8.178
8.174
7.693
7.674
7.659
7.656
7.550
7.530
7.511
7.371
7.367
7.363
7.350
7.346
7.332
7.314
7.311
7.303
7.297
7.257
7.248
7.240
7.224
6.942
6.934
4.955
4.945
4.934
4.918
4.915
4.845
4.819
4.806
4.631
4.622
3.821
3.808
O_2N
Ph
O
O
O
S
N
Bn
3w
2.20
1.21
2.28
3.20
3.25
2.08
2.19
1.05
2.00
1.97
11.0 10.5 10.0 9.5 9.0 8.5 8.0 7.5 7.0 6.5 6.0 5.5 5.0 4.5 4.0 3.5 3.0 2.5 2.0 1.5 1.0 0.5 0.0 -0.5 -1.0

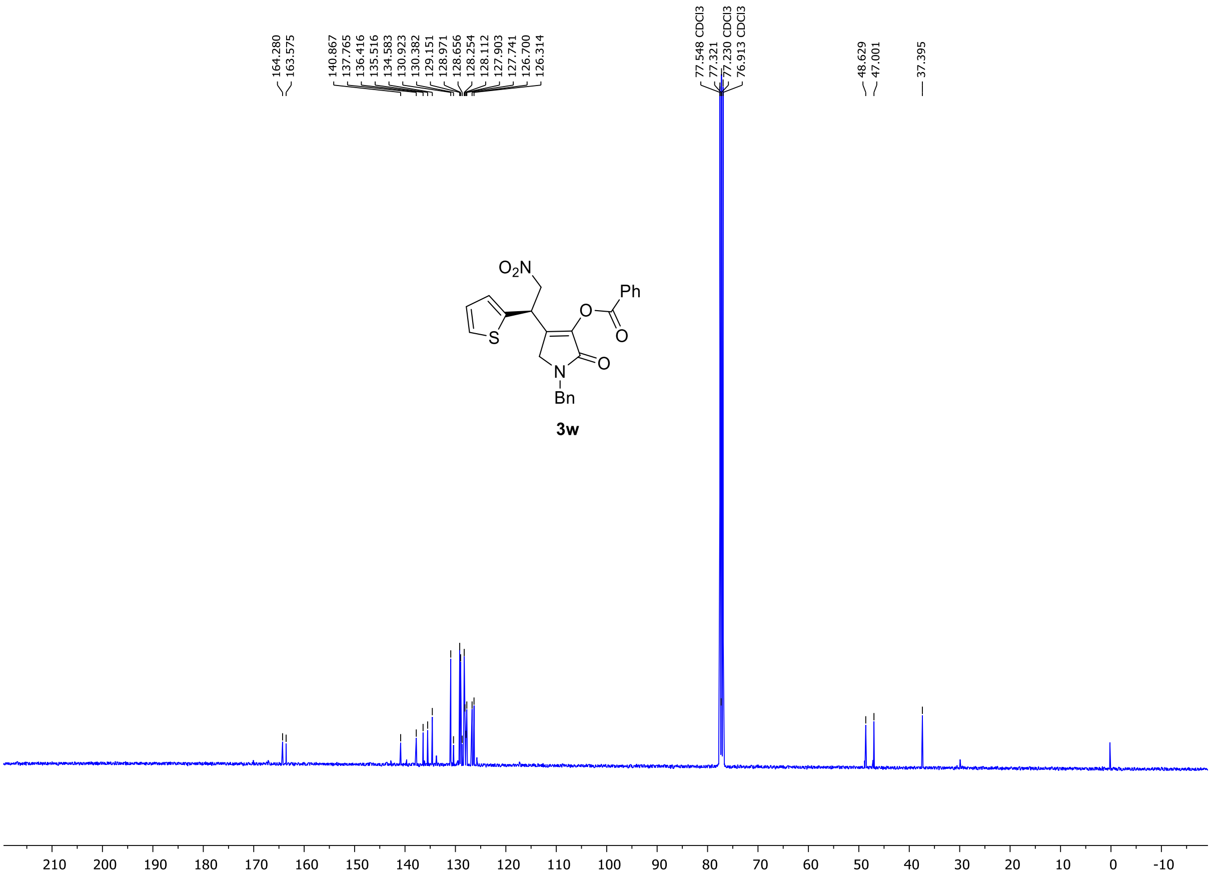
164.280
163.575
140.867
137.765
136.416
135.516
134.583
130.923
130.382
129.151
128.971
128.656
128.254
128.112
127.903
127.741
126.700
126.314
77.548 CDCl3
77.321
77.230 CDCl3
76.913 CDCl3
48.629
47.001
37.395
O_2N
Ph
O
O
O
S
N
Bn
3w
210 200 190 180 170 160 150 140 130 120 110 100 90 80 70 60 50 40 30 20 10 0 -10

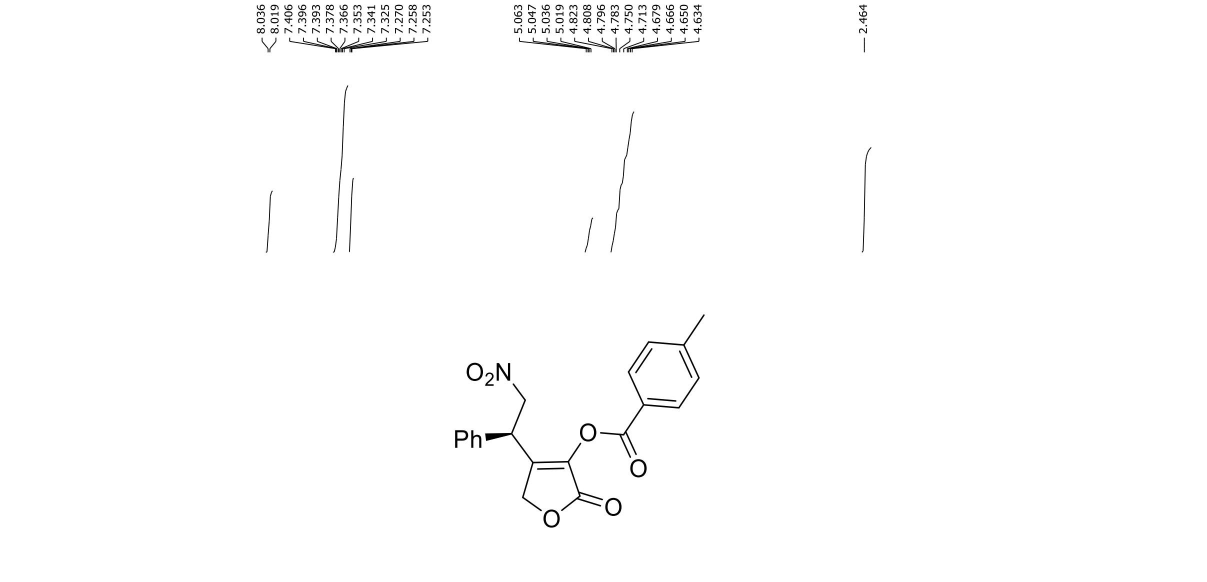

5a

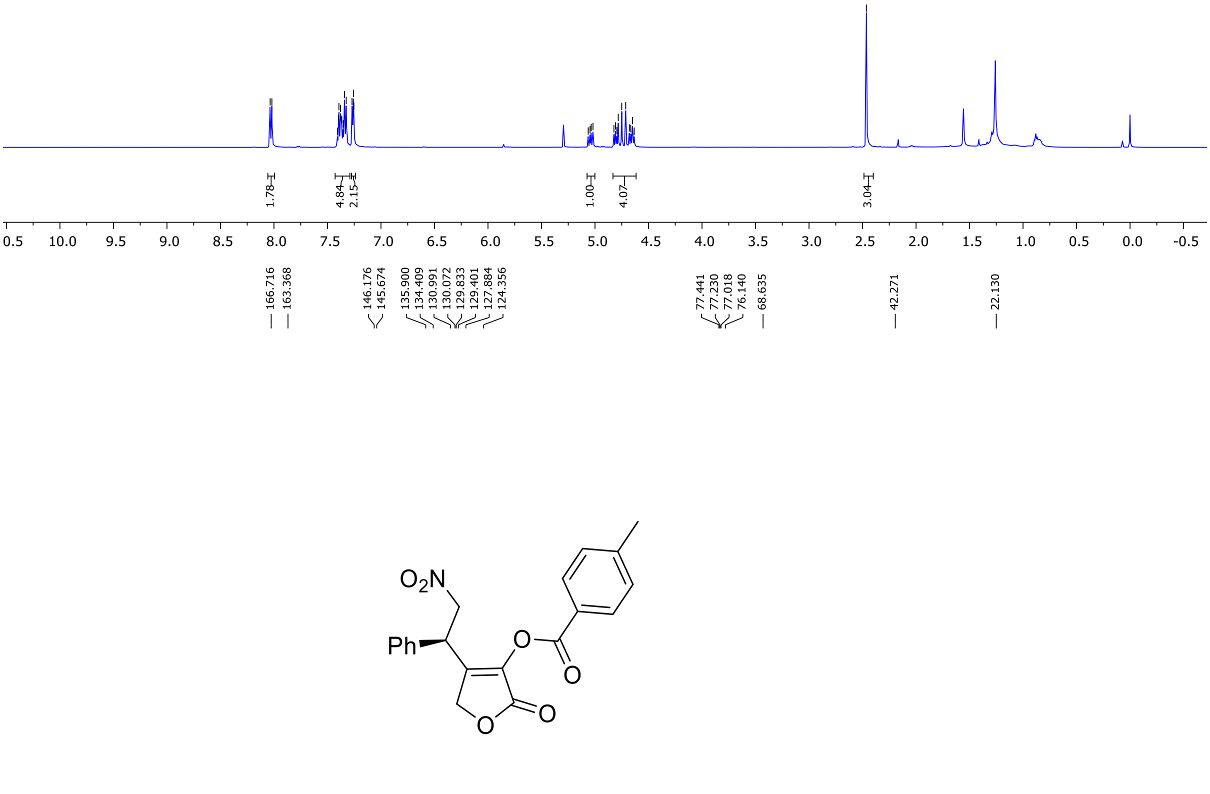

5a

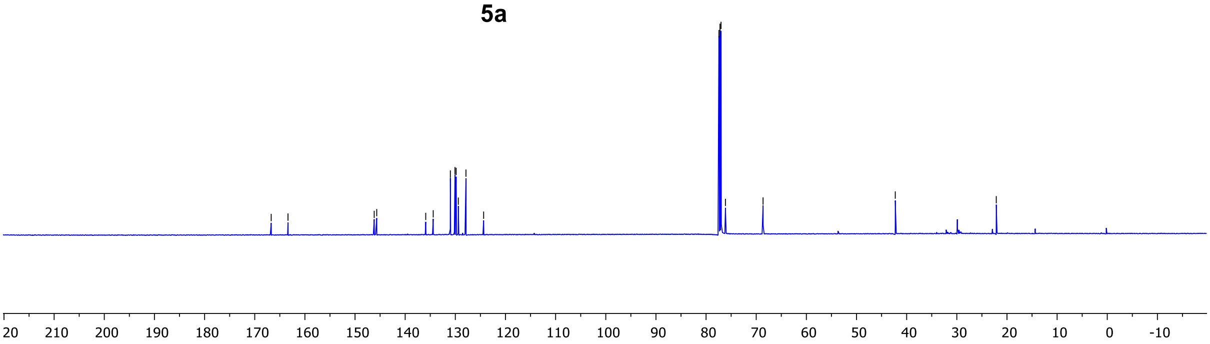

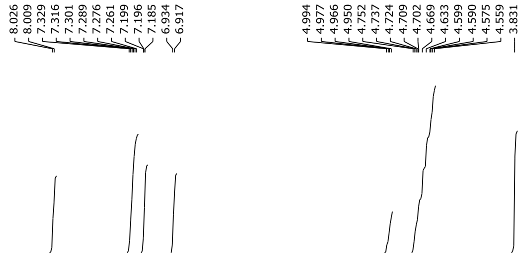

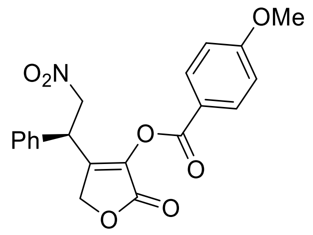

5b

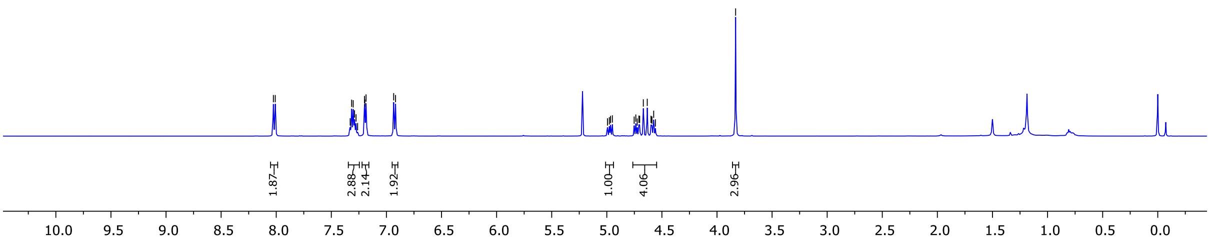

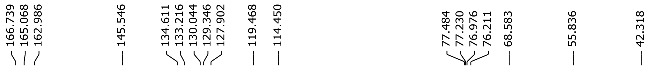

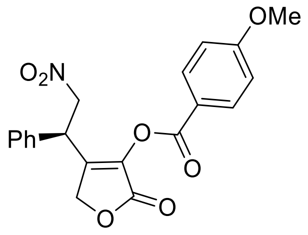

5b

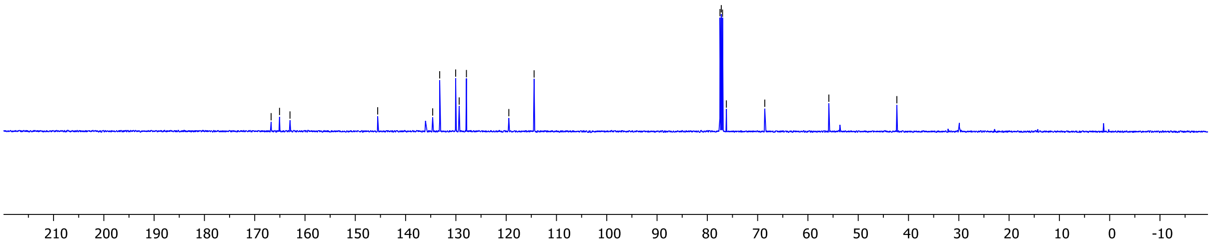

7. HPLC spectra of the products

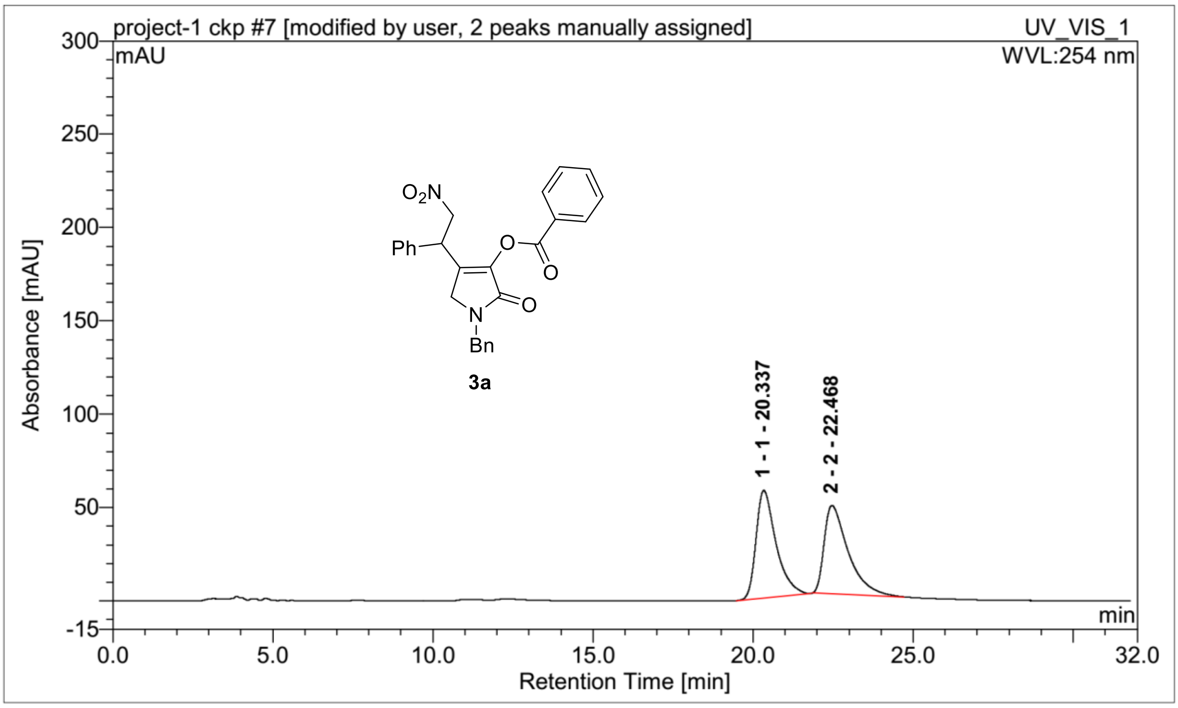

No.	Peak Name	Ret.Time (detected) min	Area mAU*min	Rel.Area(ident.) %	Height mAU	Amount
1	1	20.34	41.85811	50.38787979	57.64804	n.a.
2	2	22.47	41.214	49.61212021	47.394	n.a.

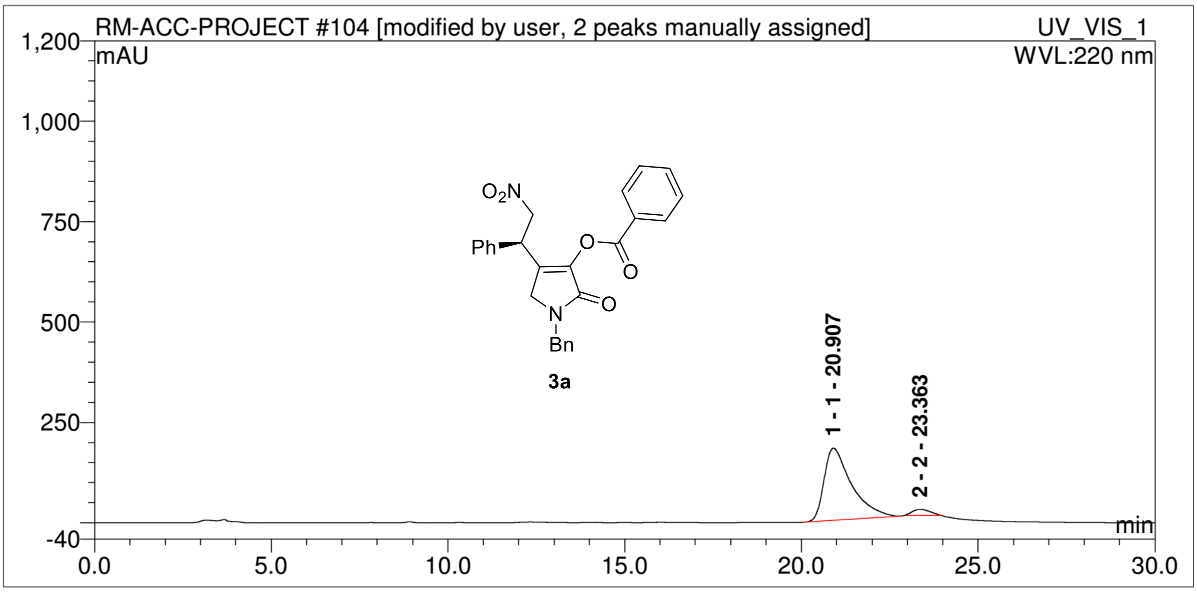

No.	Peak Name	Ret.Time (detected) min	Area mAU*min	Rel.Area(ident.) %	Height mAU	Amount
1	1	20.91	161.7176	94.83129978	179.7468	n.a.
2	2	23.36	8.814	5.168700222	14.981	n.a.

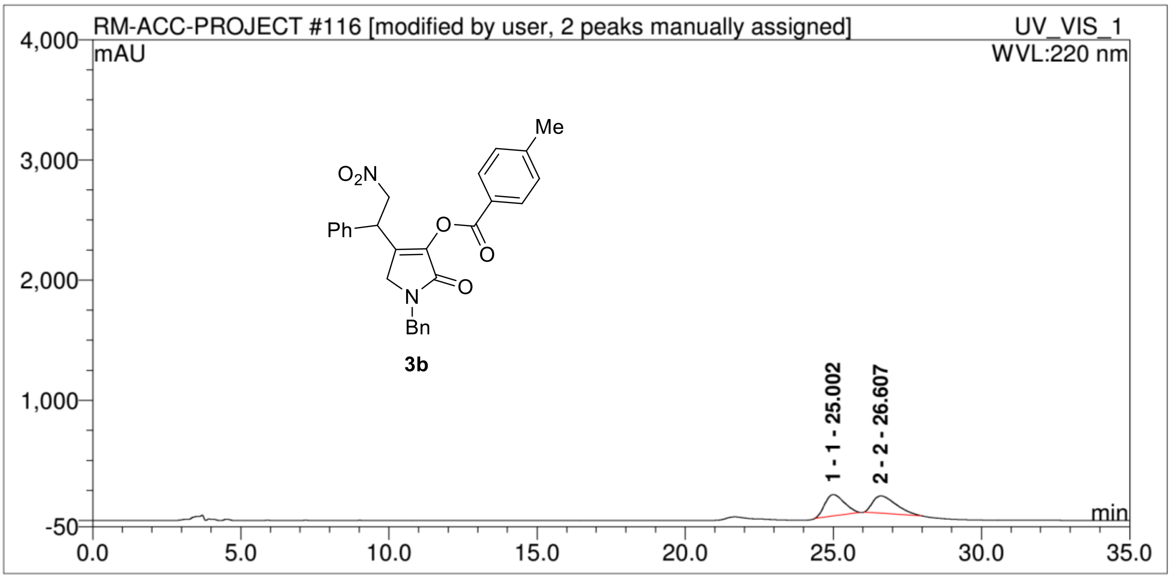

No.	Peak Name	Ret.Time (detected) min	Area mAU*min	Rel.Area(ident.) %	Height mAU	Amount
1	1	25.00	134.1507	50.49742646	177.5183	n.a.
2	2	26.61	131.508	49.50257354	143.190	n.a.

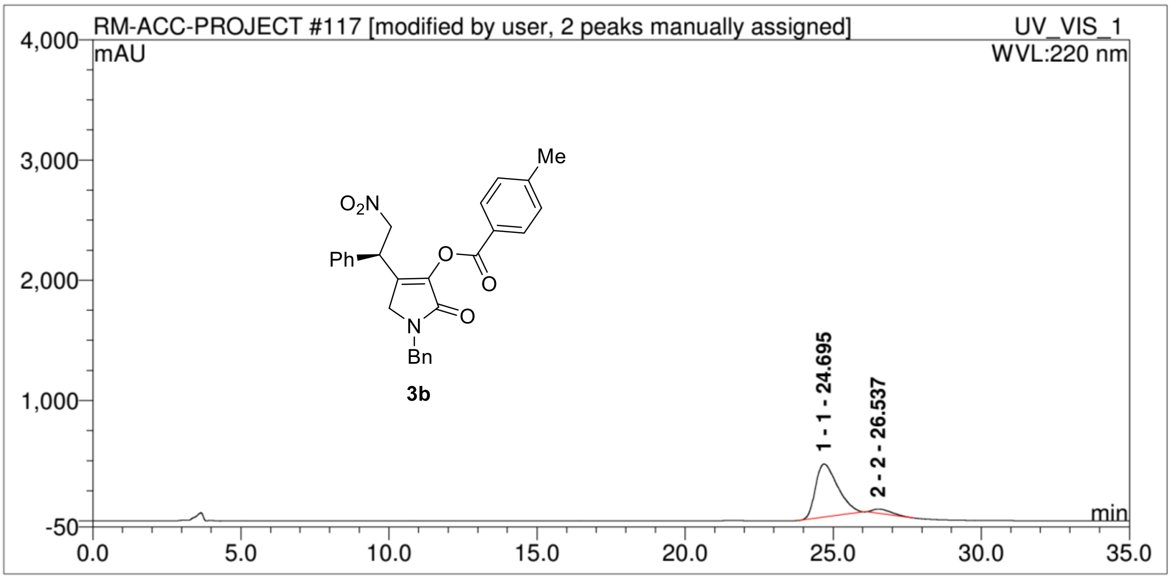

No.	Peak Name	Ret.Time (detected) min	Area mAU*min	Rel.Area(ident.) %	Height mAU	Amount
1	1	24.70	384.0506	93.66713089	440.6287	n.a.
2	2	26.54	25.966	6.332869113	34.231	n.a.

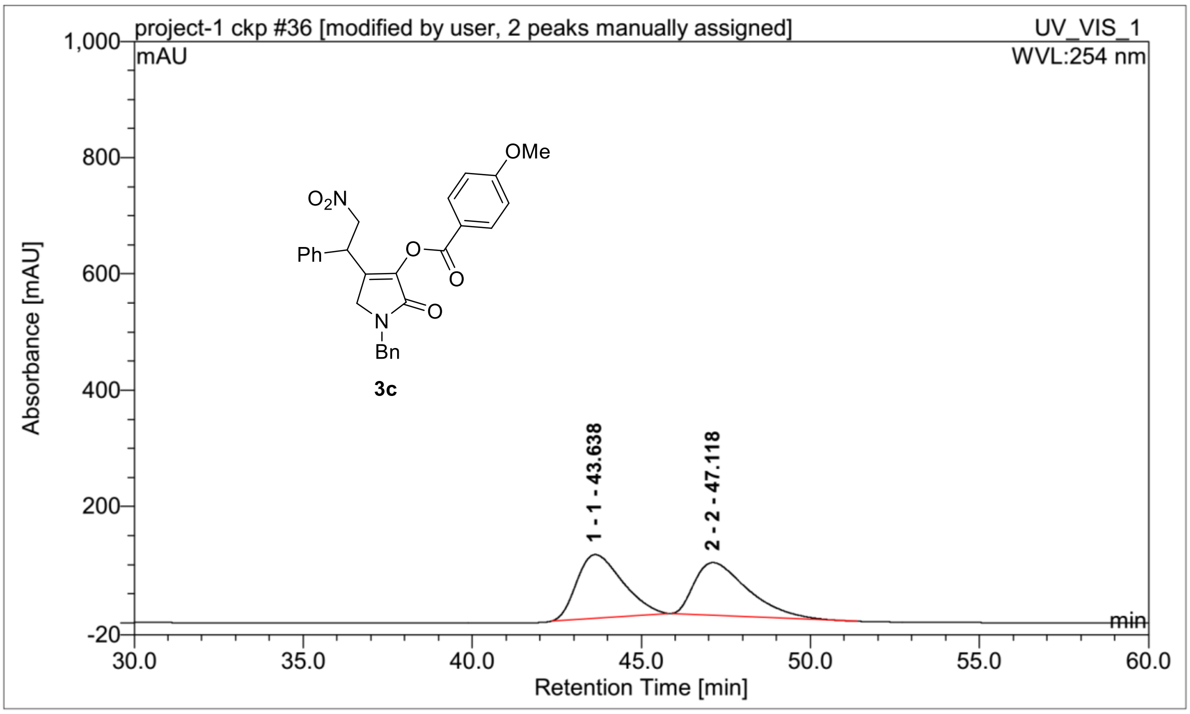

No.	Peak Name	Ret.Time (detected)	Area	Rel.Area(ident.)	Height	Amount
		min	mAU*min	%	mAU	
1	1	43.64	170.4751	50.71063113	109.3246	n.a.
2	2	47.12	165.697	49.28936887	90.290	n.a.

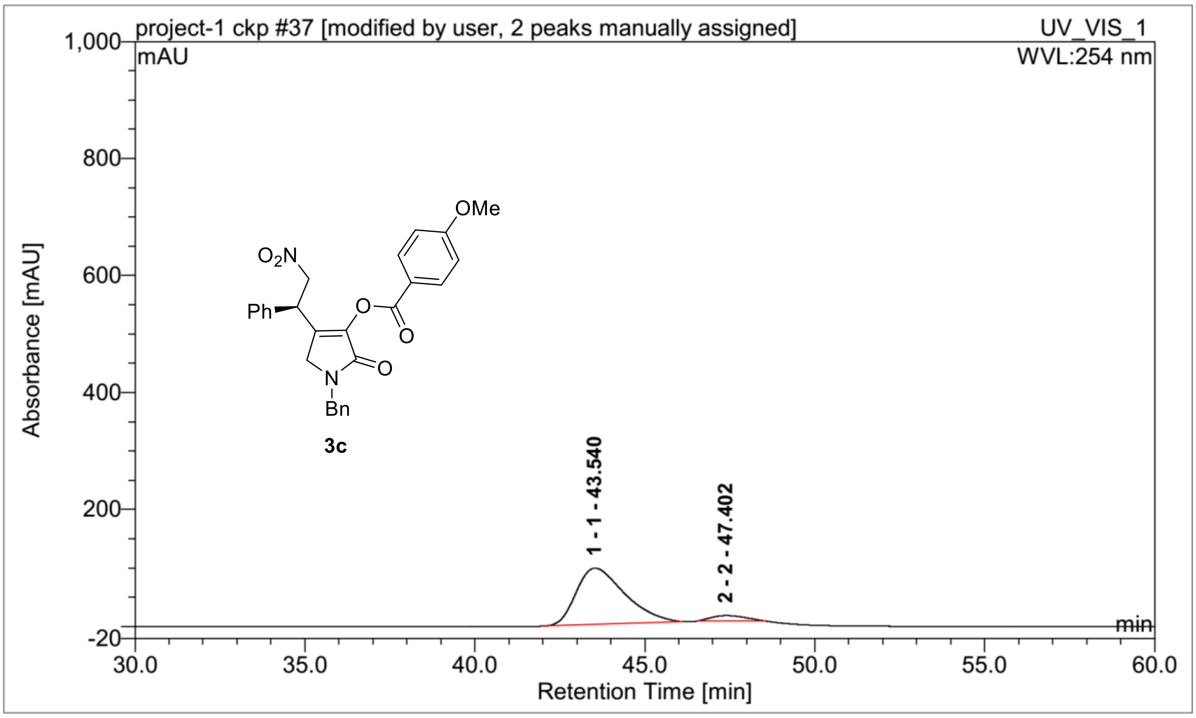

No.	Peak Name	Ret.Time (detected)	Area	Rel.Area(ident.)	Height	Amount
		min	mAU*min	%	mAU	
1	1	43.54	159.3508	93.55762674	96.30458	n.a.
2	2	47.40	10.973	6.442373257	9.368	n.a.

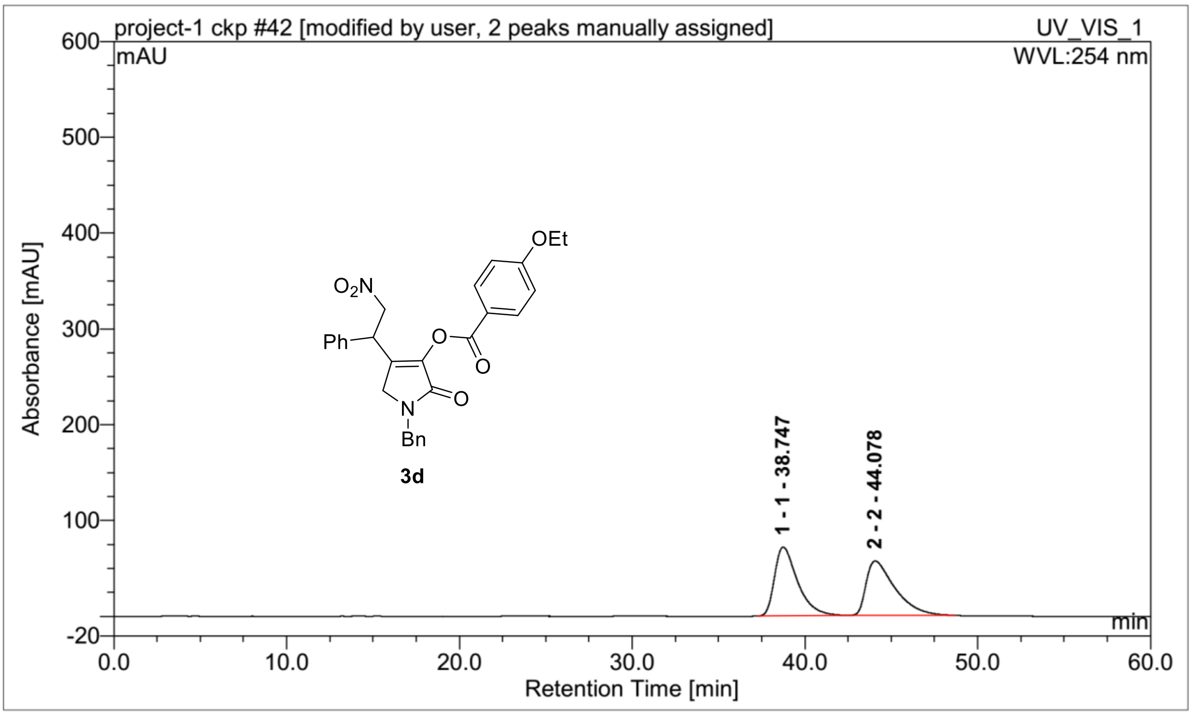

No.	Peak Name	Ret.Time (detected) min	Area mAU*min	Rel.Area(ident.) %	Height mAU	Amount
1	1	38.75	110.3236	50.23289711	71.69794	n.a.
2	2	44.08	109.301	49.76710289	56.554	n.a.

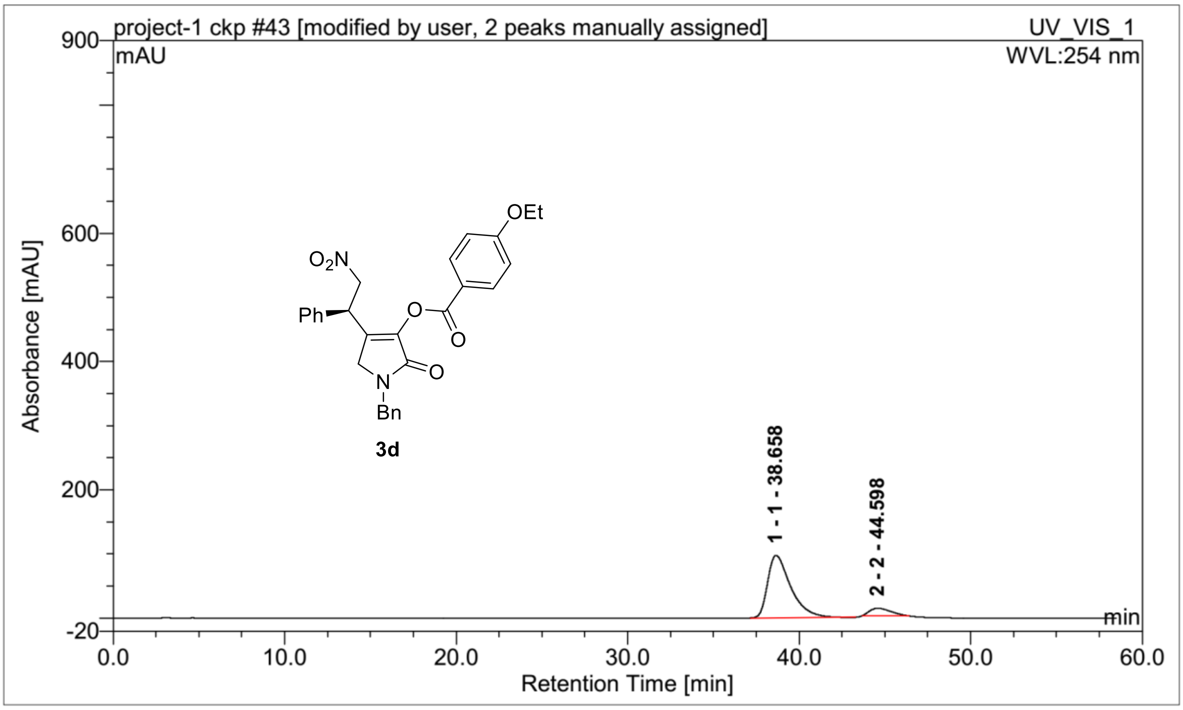

No.	Peak Name	Ret.Time (detected) min	Area mAU*min	Rel.Area(ident.) %	Height mAU	Amount
1	1	38.66	151.6129	89.74085595	97.43533	n.a.
2	2	44.60	17.332	10.25914405	11.910	n.a.

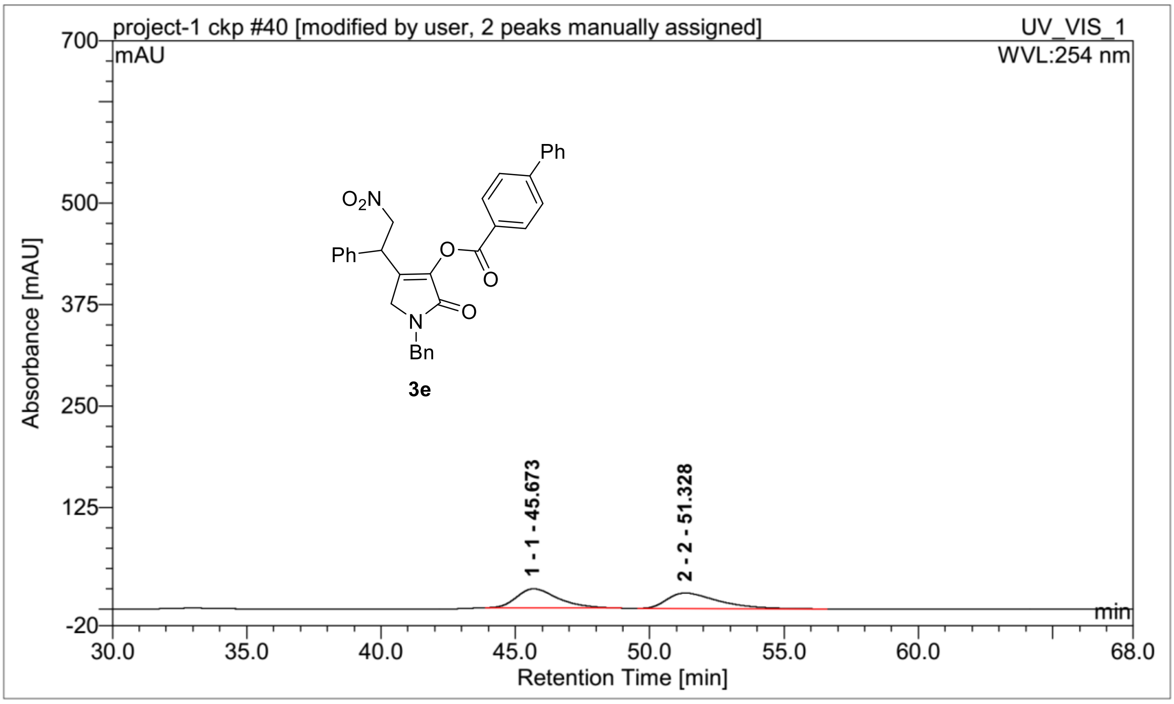

No.	Peak Name	Ret.Time (detected) min	Area mAU*min	Rel.Area(ident.) %	Height mAU	Amount
1	1	45.67	42.00401	49.96577891	23.43344	n.a.
2	2	51.33	42.062	50.03422109	19.054	n.a.

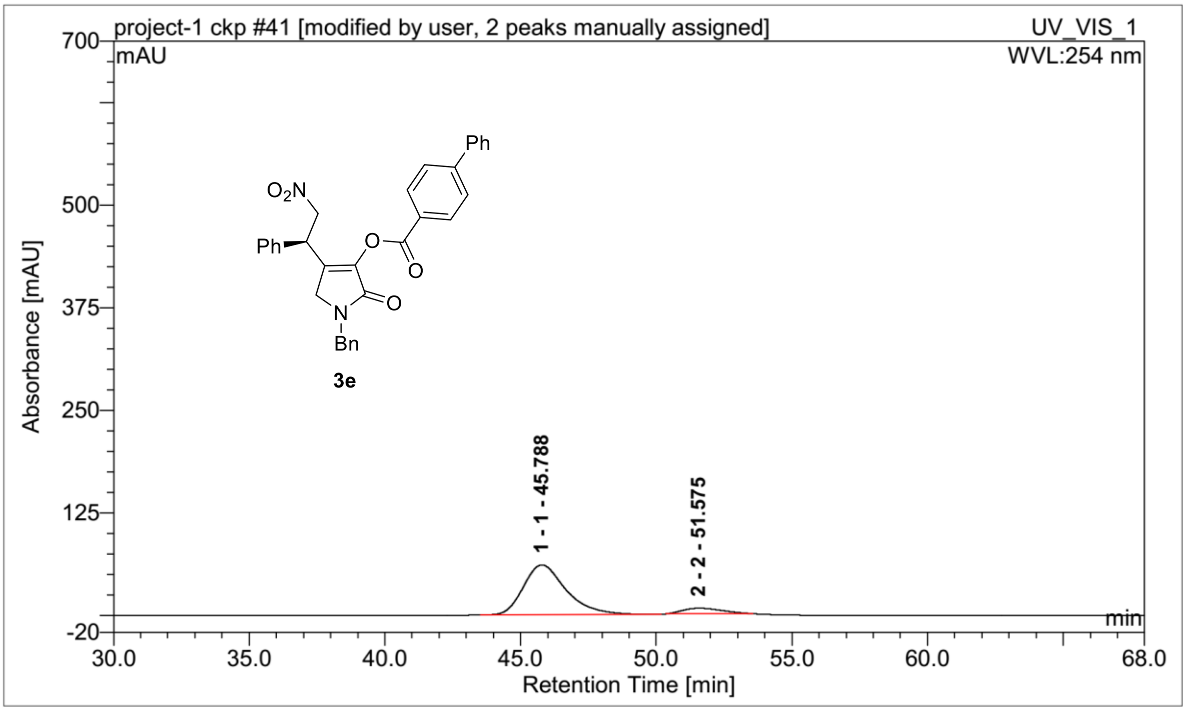

No.	Peak Name	Ret.Time (detected) min	Area mAU*min	Rel.Area(ident.) %	Height mAU	Amount
1	1	45.79	110.8823	90.50385156	60.79811	n.a.
2	2	51.58	11.634	9.496148443	6.870	n.a.

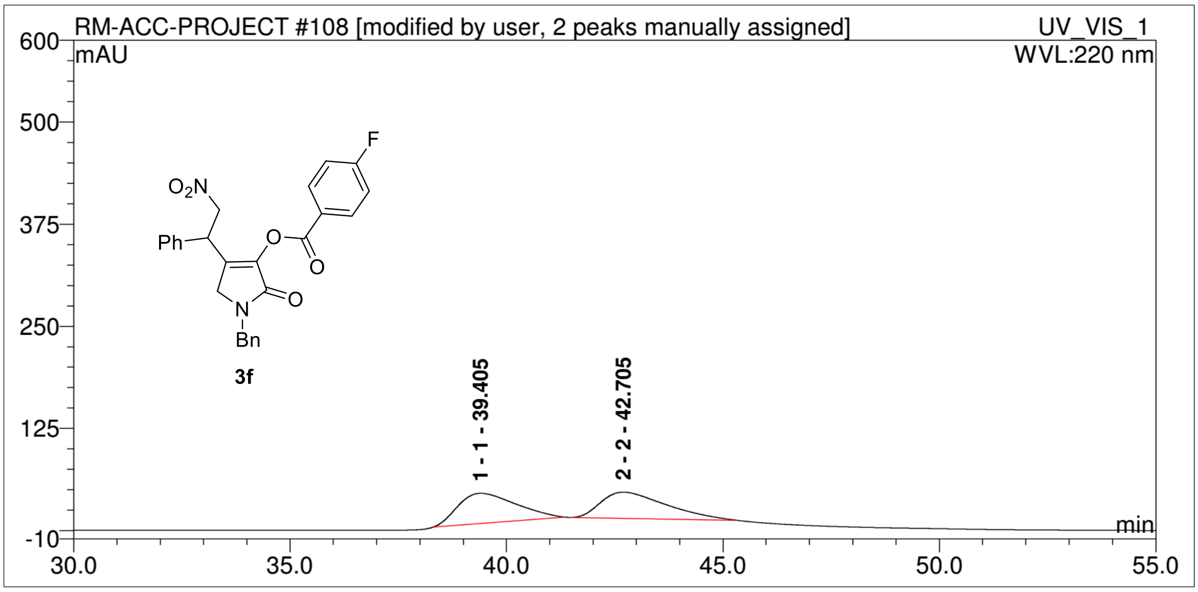

No.		Peak Name	Ret.Time (detected) min	Area mAU*min	Rel.Area(ident.) %	Height mAU	Amount
1	1		39.41	57.21393	50.46393603	36.941	n.a.
2	2		42.71	56.162	49.53606397	32.017	n.a.

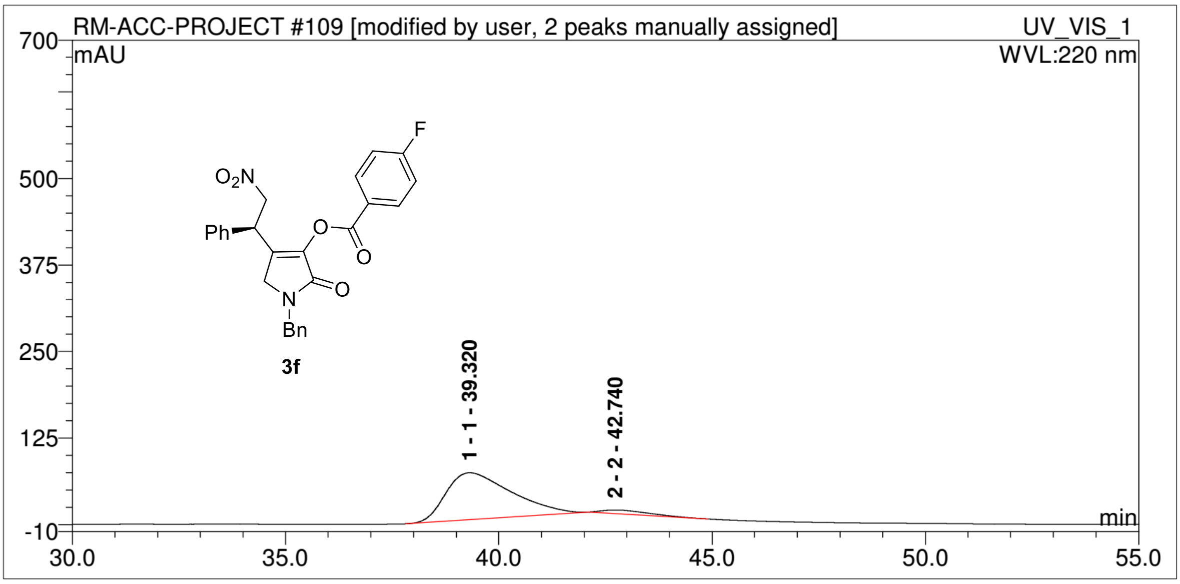

No.		Peak Name	Ret.Time (detected) min	Area mAU*min	Rel.Area(ident.) %	Height mAU	Amount
1	1		39.32	119.8521	94.53301927	67.85756	n.a.
2	2		42.74	6.931	5.466980731	5.153	n.a.

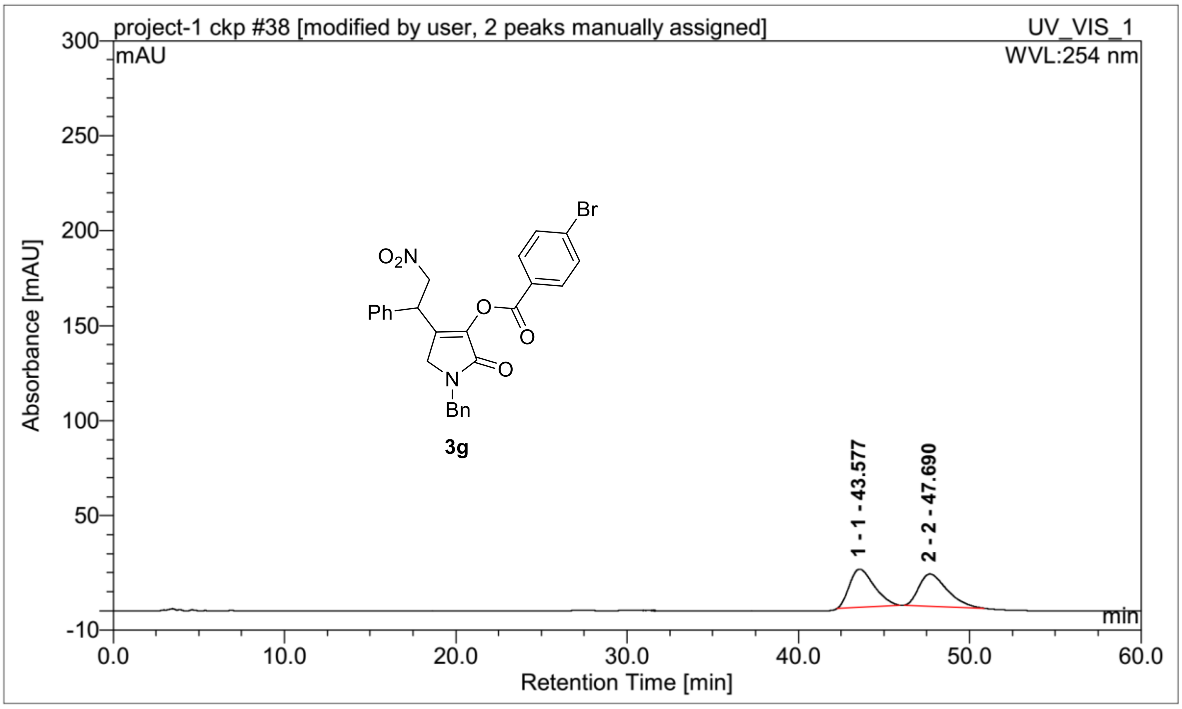

No.	Peak Name	Ret.Time (detected) min	Area mAU*min	Rel.Area(ident.) %	Height mAU	Amount
1	1	43.58	33.53818	50.82017884	20.08853	n.a.
2	2	47.69	32.456	49.17982116	16.978	n.a.

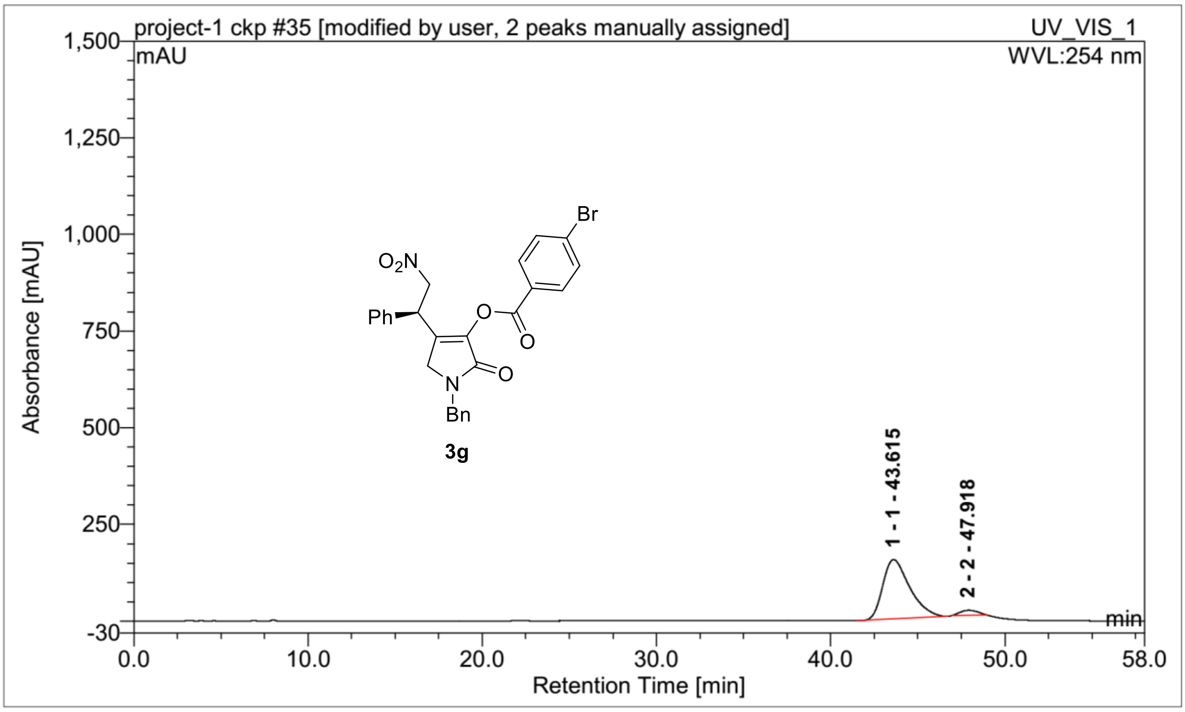

No.	Peak Name	Ret.Time (detected) min	Area mAU*min	Rel.Area(ident.) %	Height mAU	Amount
1	1	43.62	275.2487	94.52827747	153.6061	n.a.
2	2	47.92	15.933	5.47172253	13.348	n.a.

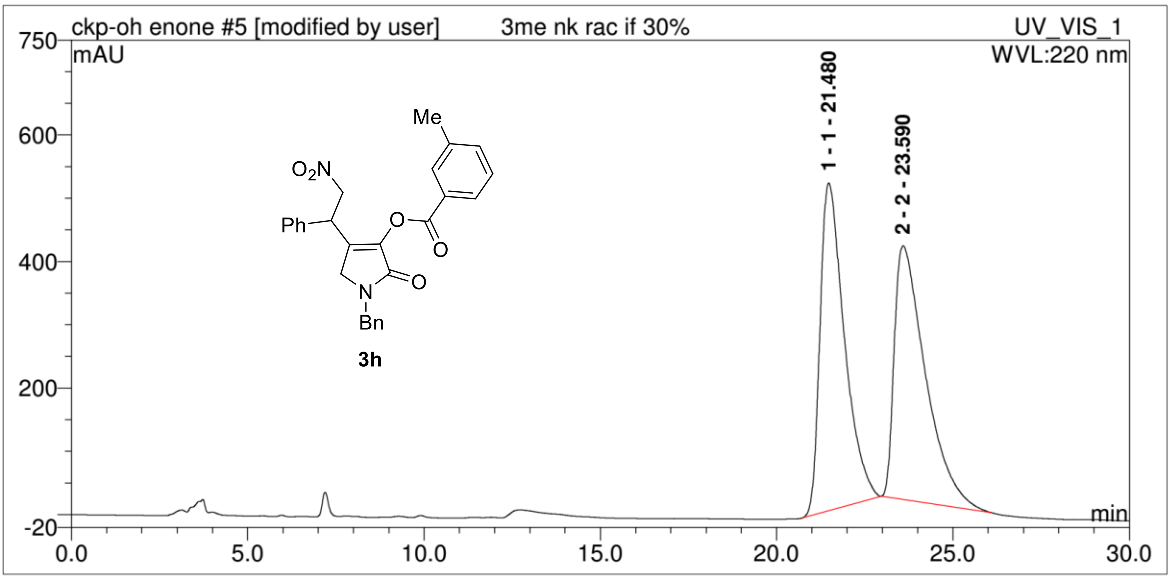

No.	Peak Name	Ret.Time (detected) min	Area mAU*min	Rel.Area(ident.) %	Height mAU	Amount
1	1	21.48	414.3443	50.20578505	518.0697	n.a.
2	2	23.59	410.948	49.79421495	400.873	n.a.

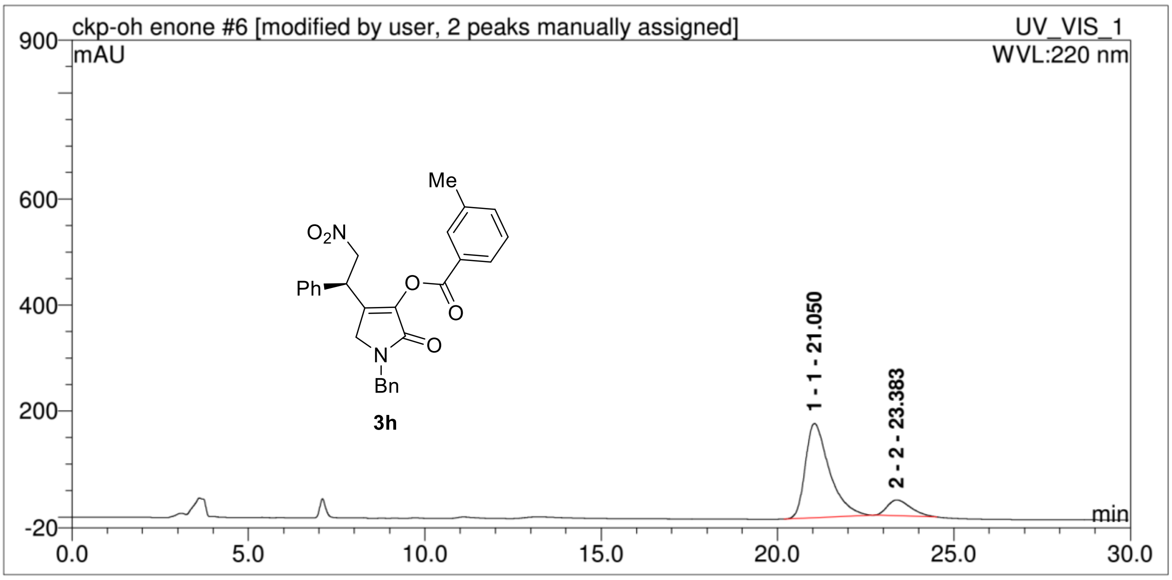

No.	Peak Name	Ret.Time (detected) min	Area mAU*min	Rel.Area(ident.) %	Height mAU	Amount
1	1	21.05	140.9552	86.09649053	177.7406	n.a.
2	2	23.38	22.763	13.90350947	29.591	n.a.

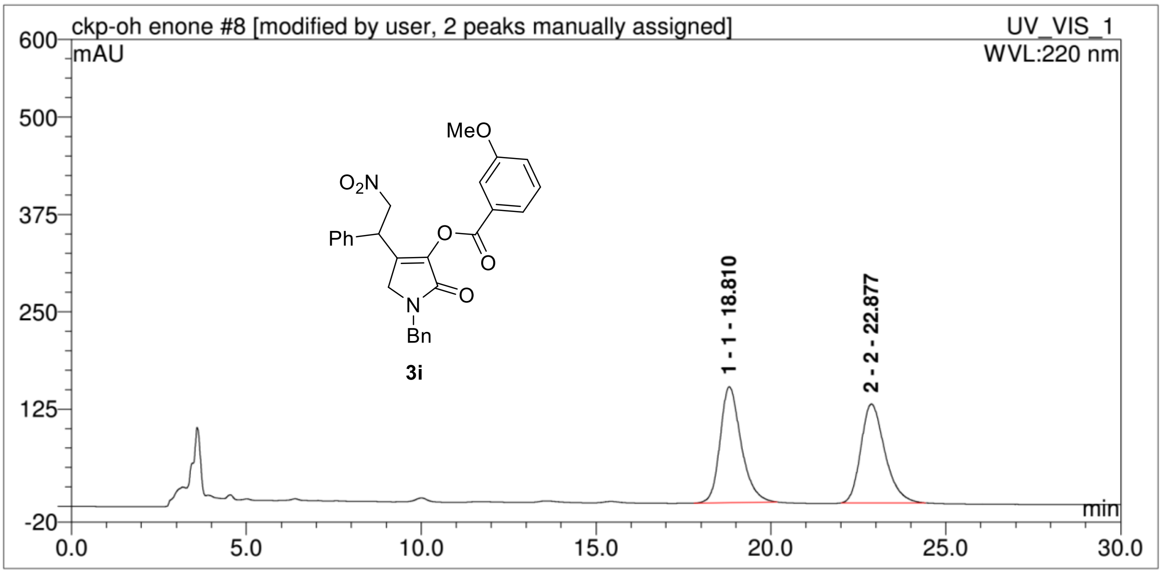

No.	Peak Name	Ret.Time (detected) min	Area mAU*min	Rel.Area(ident.) %	Height mAU	Amount
1	1	18.81	104.2595	50.75992276	148.9903	n.a.
2	2	22.88	101.138	49.24007724	127.020	n.a.

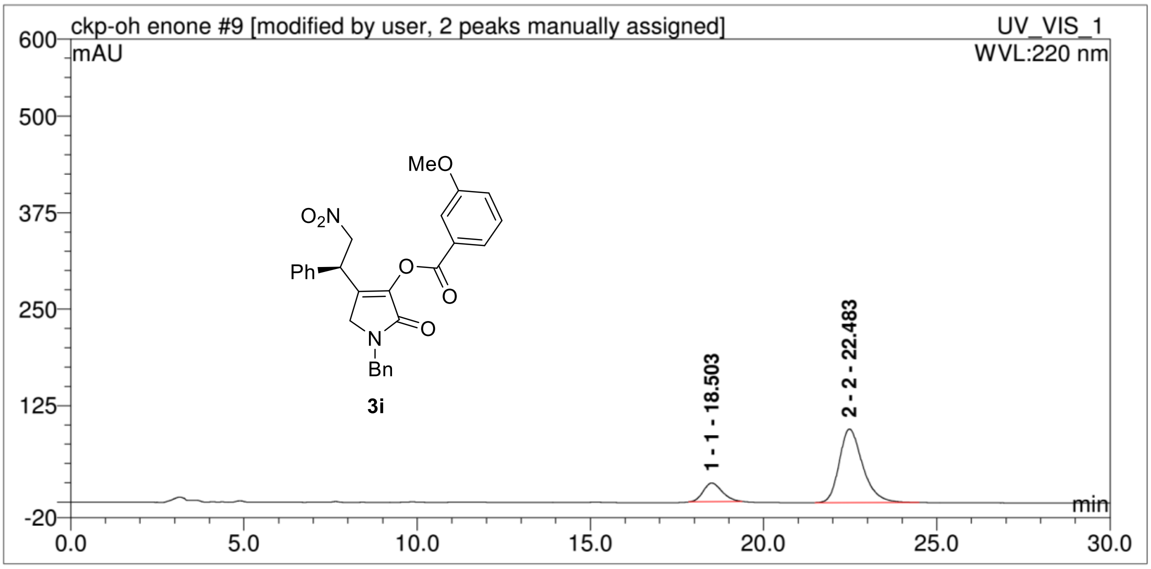

No.	Peak Name	Ret.Time (detected) min	Area mAU*min	Rel.Area(ident.) %	Height mAU	Amount
1	1	18.50	15.2793	17.00958056	24.15859	n.a.
2	2	22.48	74.548	82.99041944	95.068	n.a.

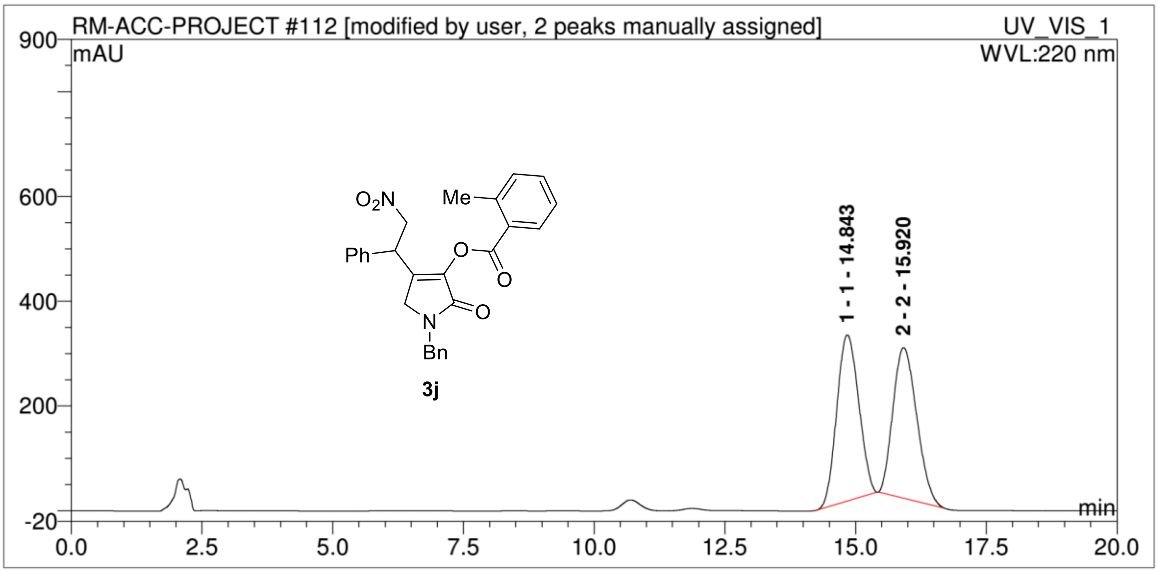

No.	Peak Name	Ret.Time (detected) min	Area mAU*min	Rel.Area(ident.) %	Height mAU	Amount
1	1	14.84	154.1613	50.35579852	316.8359	n.a.
2	2	15.92	151.983	49.64420148	287.411	n.a.

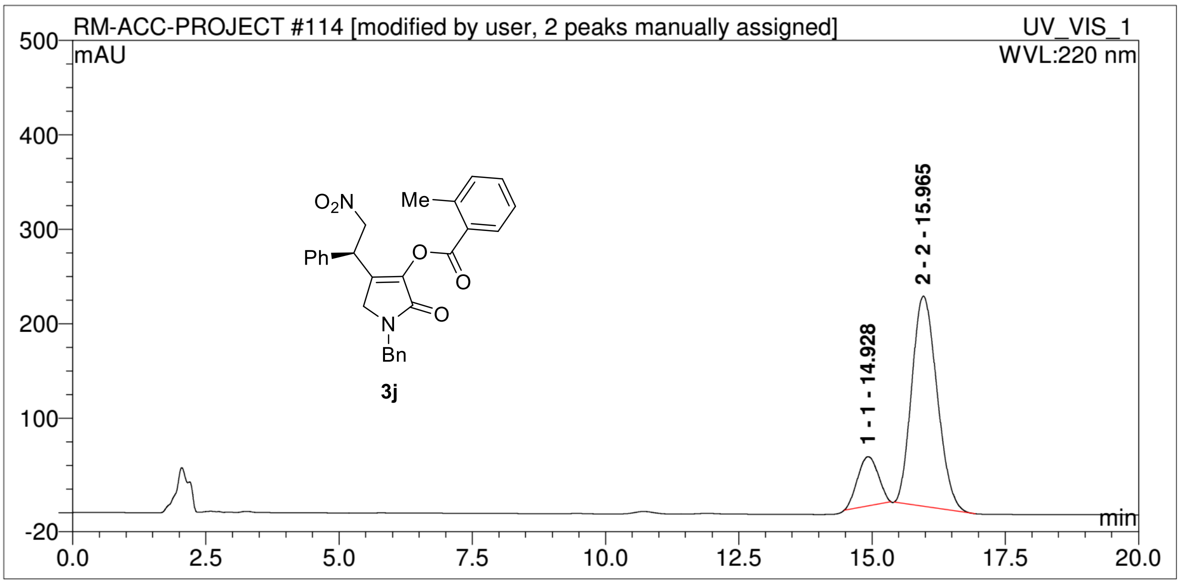

No.	Peak Name	Ret.Time (detected) min	Area mAU*min	Rel.Area(ident.) %	Height mAU	Amount
1	1	14.93	23.30586	16.01132578	52.02937	n.a.
2	2	15.97	122.253	83.98867422	222.411	n.a.

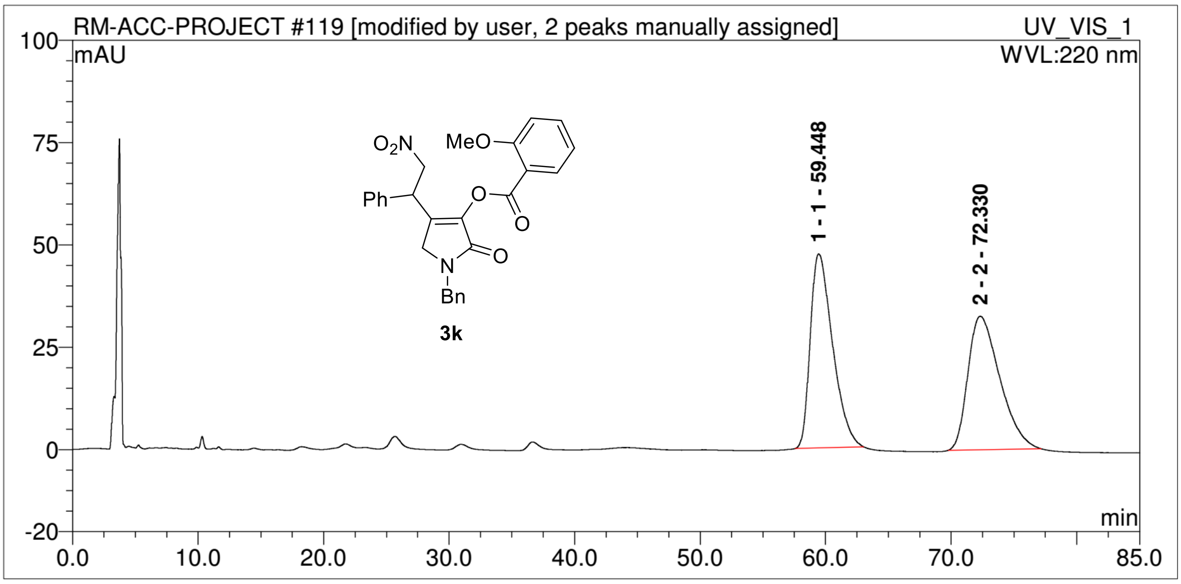

No.	Peak Name	Ret.Time (detected) min	Area mAU*min	Rel.Area(ident.) %	Height mAU	Amount
1	1	59.45	97.71814	50.42766045	47.38599	n.a.
2	2	72.33	96.061	49.57233955	32.610	n.a.

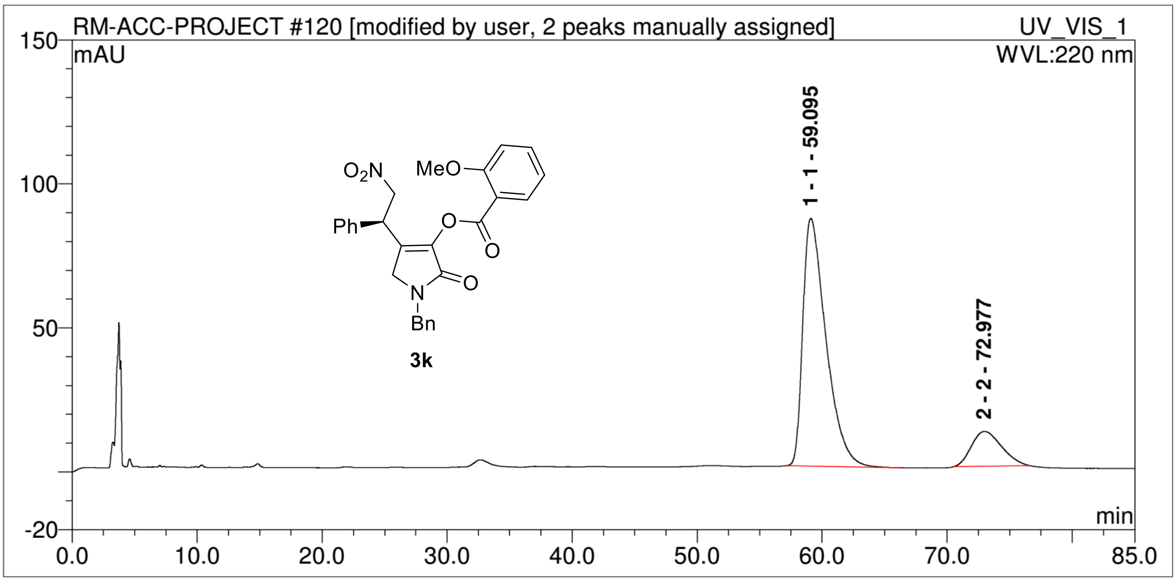

No.	Peak Name	Ret.Time (detected) min	Area mAU*min	Rel.Area(ident.) %	Height mAU	Amount
1	1	59.10	184.8975	84.49419517	86.0416	n.a.
2	2	72.98	33.931	15.50580483	12.084	n.a.

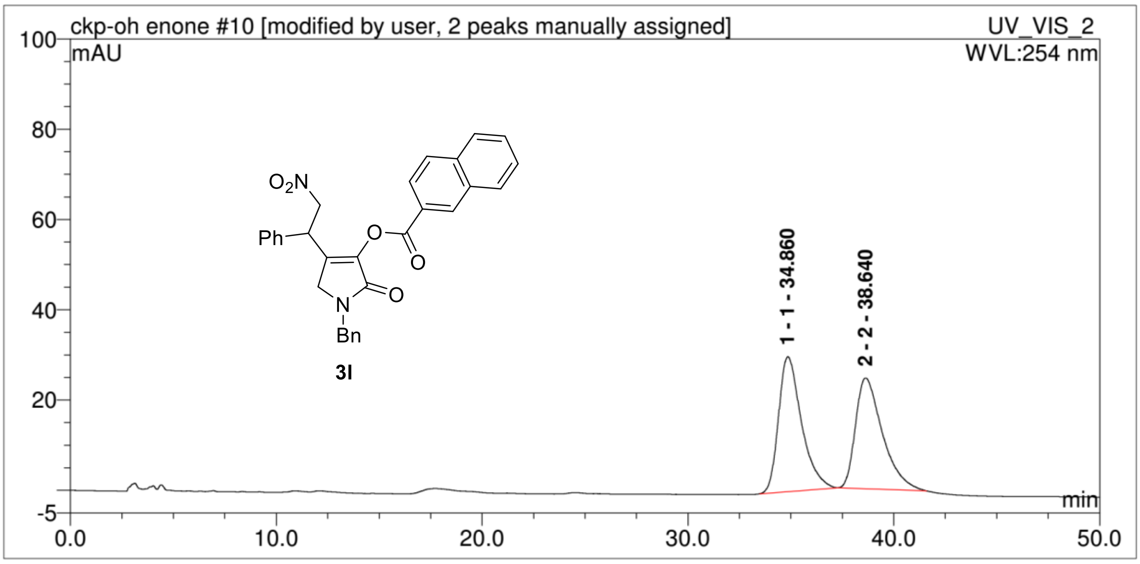

No.	Peak Name	Ret.Time (detected) min	Area mAU*min	Rel.Area(ident.) %	Height mAU	Amount
1	1	34.86	38.71484	50.95594947	29.89739	n.a.
2	2	38.64	37.262	49.04405053	24.533	n.a.

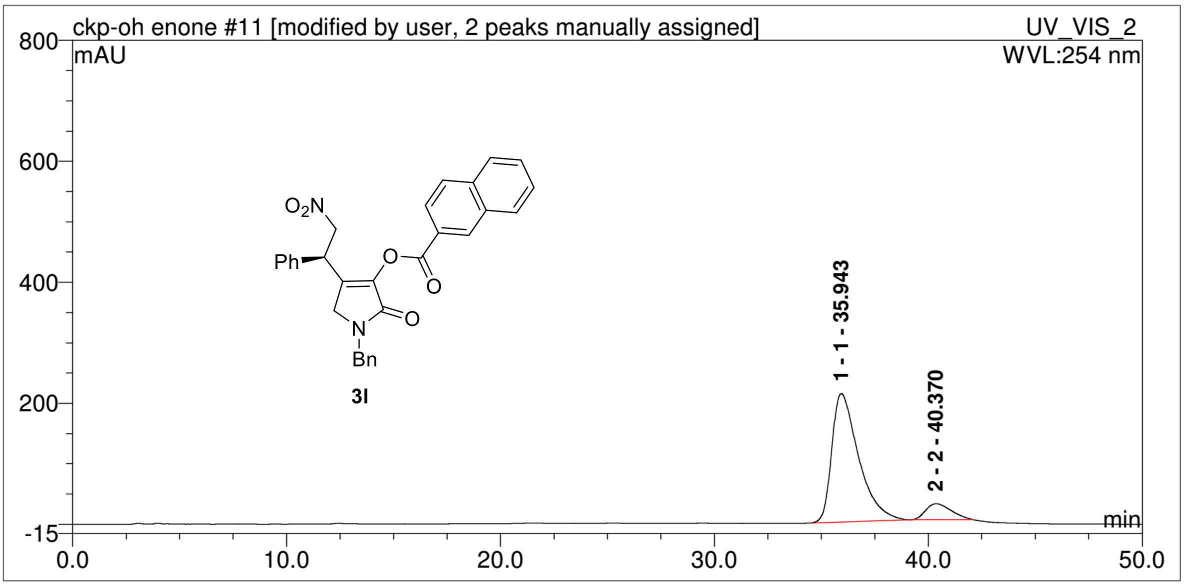

No.	Peak Name	Ret.Time (detected) min	Area mAU*min	Rel.Area(ident.) %	Height mAU	Amount
1	1	35.94	308.0256	89.59628812	212.0375	n.a.
2	2	40.37	35.767	10.40371188	26.049	n.a.

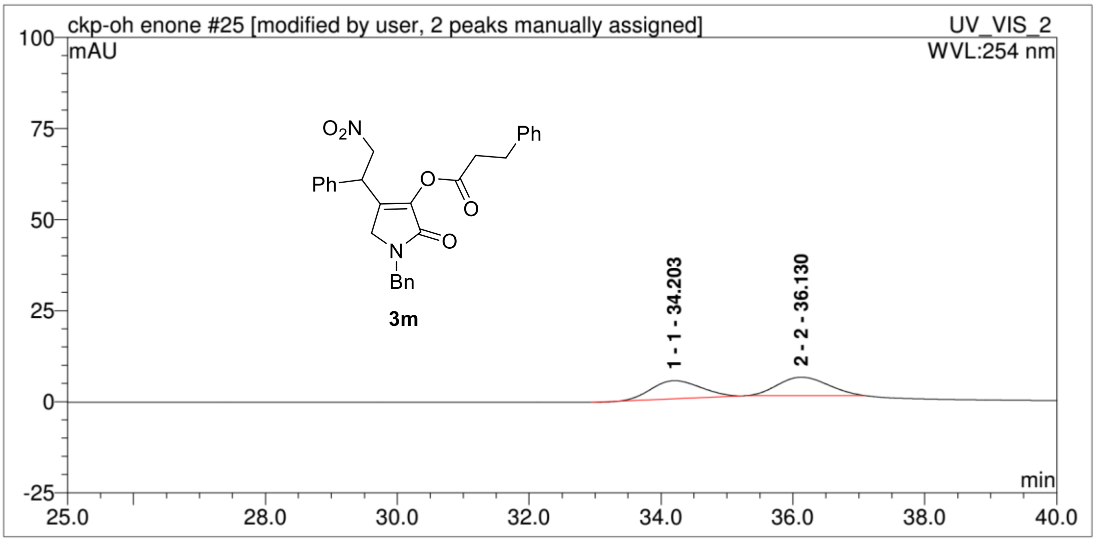

No.	Peak Name	Ret.Time (detected) min	Area mAU*min	Rel.Area(ident.) %	Height mAU	Amount
1	1	34.20	4.301127	48.88141027	4.97301	n.a.
2	2	36.13	4.498	51.11858973	5.077	n.a.

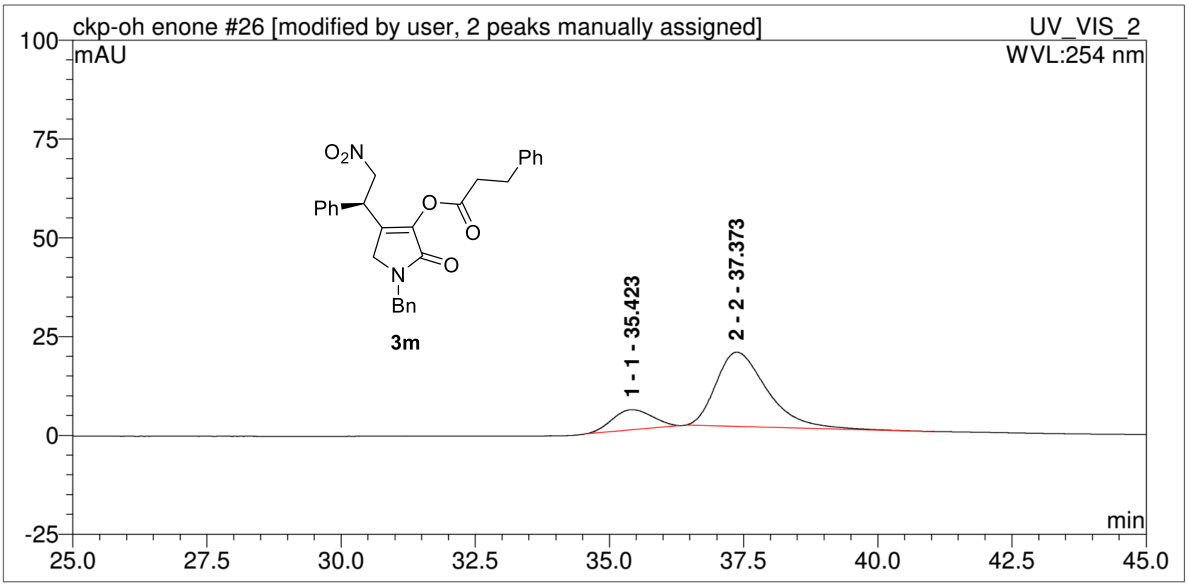

No.	Peak Name	Ret.Time (detected) min	Area mAU*min	Rel.Area(ident.) %	Height mAU	Amount
1	1	35.42	4.353664	17.34777353	5.06511	n.a.
2	2	37.37	20.743	82.65222647	18.790	n.a.

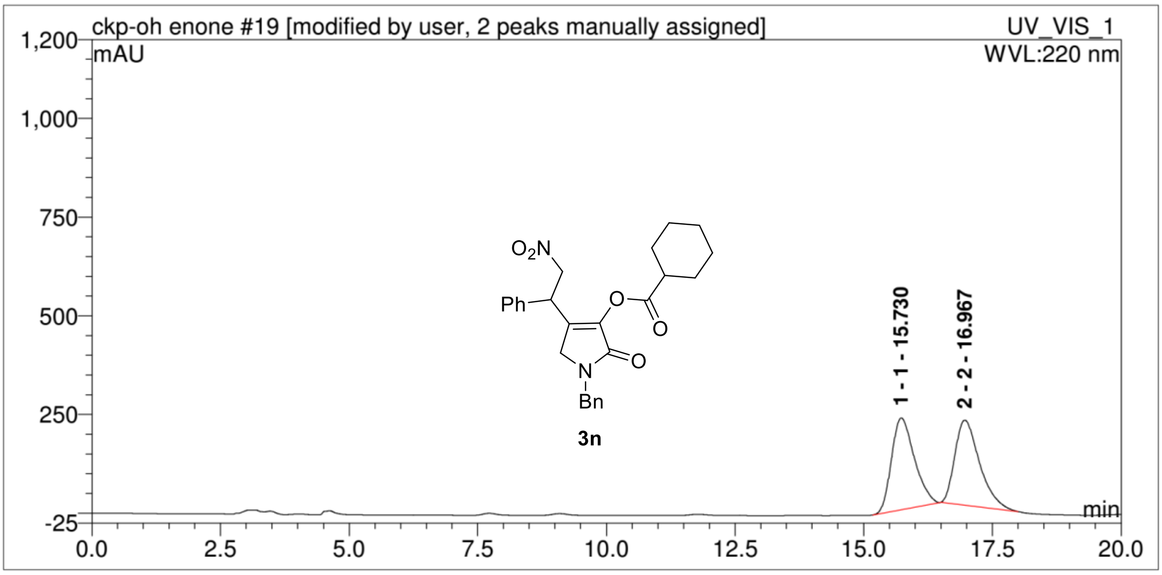

No.	Peak Name	Ret.Time (detected) min	Area mAU*min	Rel.Area(ident.) %	Height mAU	Amount
1	1	15.73	119.8505	49.94137103	232.0696	n.a.
2	2	16.97	120.132	50.05862897	215.356	n.a.

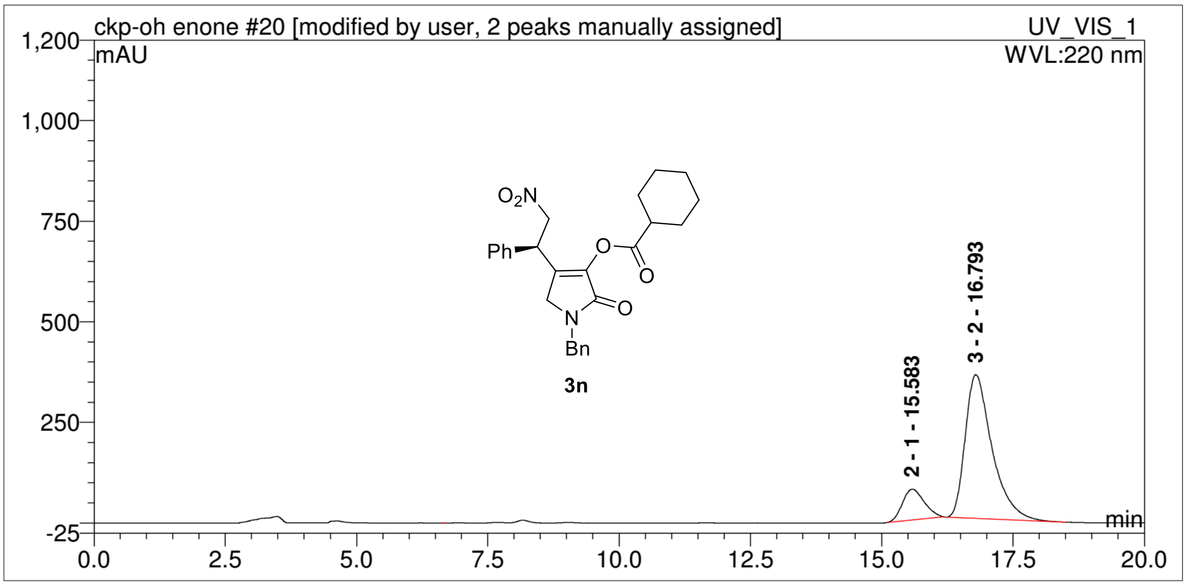

No.	Peak Name	Ret.Time (detected) min	Area mAU*min	Rel.Area(ident.) %	Height mAU	Amount
2	1	15.58	36.74058	14.74907585	76.5934	n.a.
3	2	16.79	212.364	85.25092415	357.231	n.a.

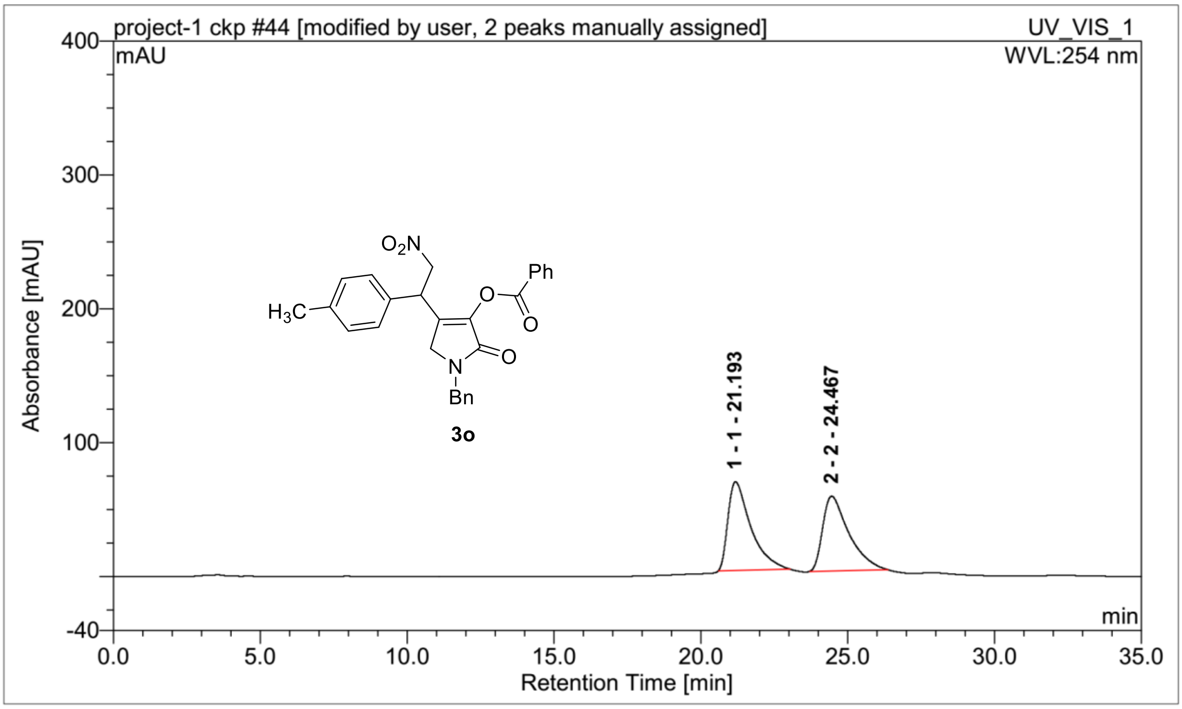

No.	Peak Name	Ret.Time (detected) min	Area mAU*min	Rel.Area(ident.) %	Height mAU	Amount
1	1	21.19	59.658	50.73121424	66.10262	n.a.
2	2	24.47	57.938	49.26878576	55.881	n.a.

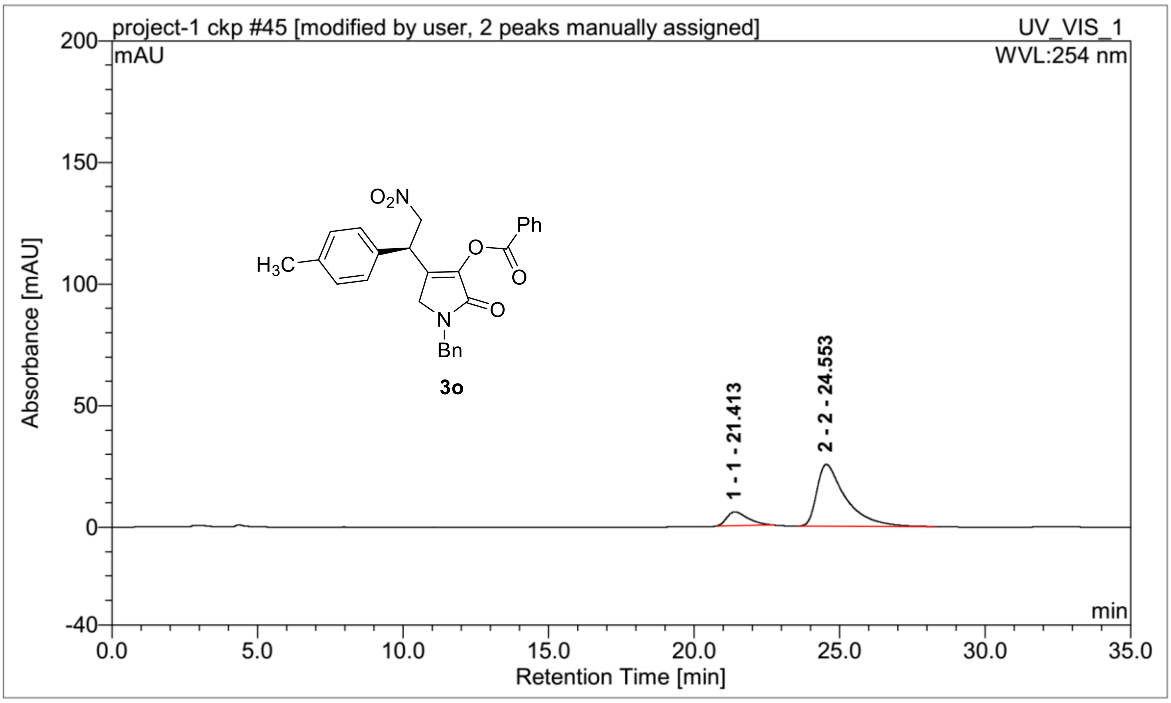

No.	Peak Name	Ret.Time (detected) min	Area mAU*min	Rel.Area(ident.) %	Height mAU	Amount
1	1	21.41	4.852714	14.22543367	5.73675	n.a.
2	2	24.55	29.260	85.77456633	25.409	n.a.

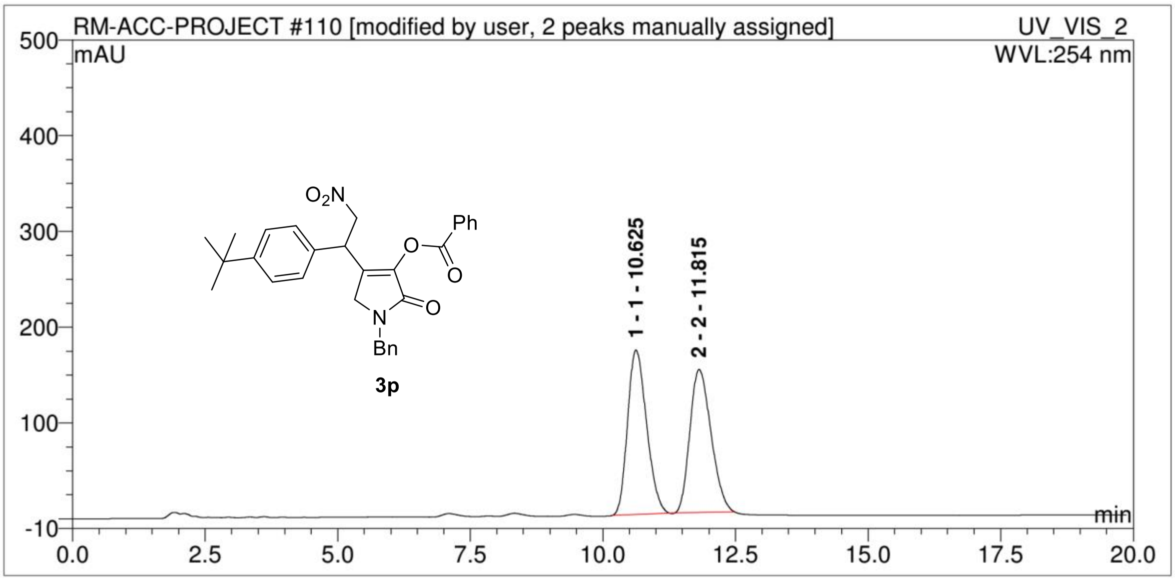

No.	Peak Name	Ret.Time (detected) min	Area mAU*min	Rel.Area(ident.) %	Height mAU	Amount
1	1	10.63	71.40227	50.73228303	171.5064	n.a.
2	2	11.82	69.341	49.26771697	149.375	n.a.

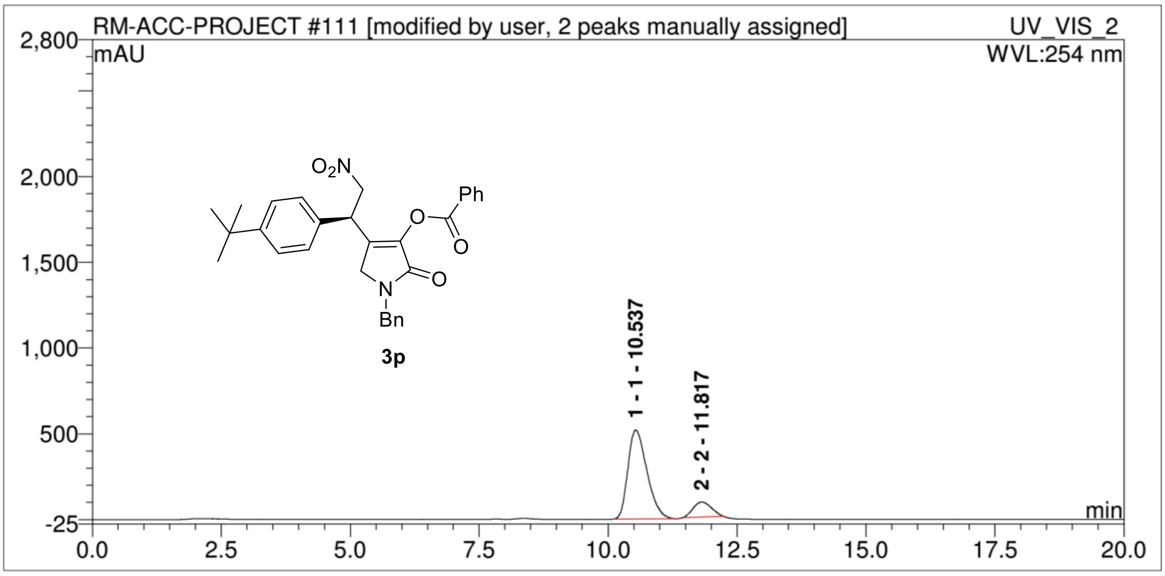

No.	Peak Name	Ret.Time (detected) min	Area mAU*min	Rel.Area(ident.) %	Height mAU	Amount
1	1	10.54	221.7415	86.06534162	519.0157	n.a.
2	2	11.82	35.902	13.93465838	87.205	n.a.

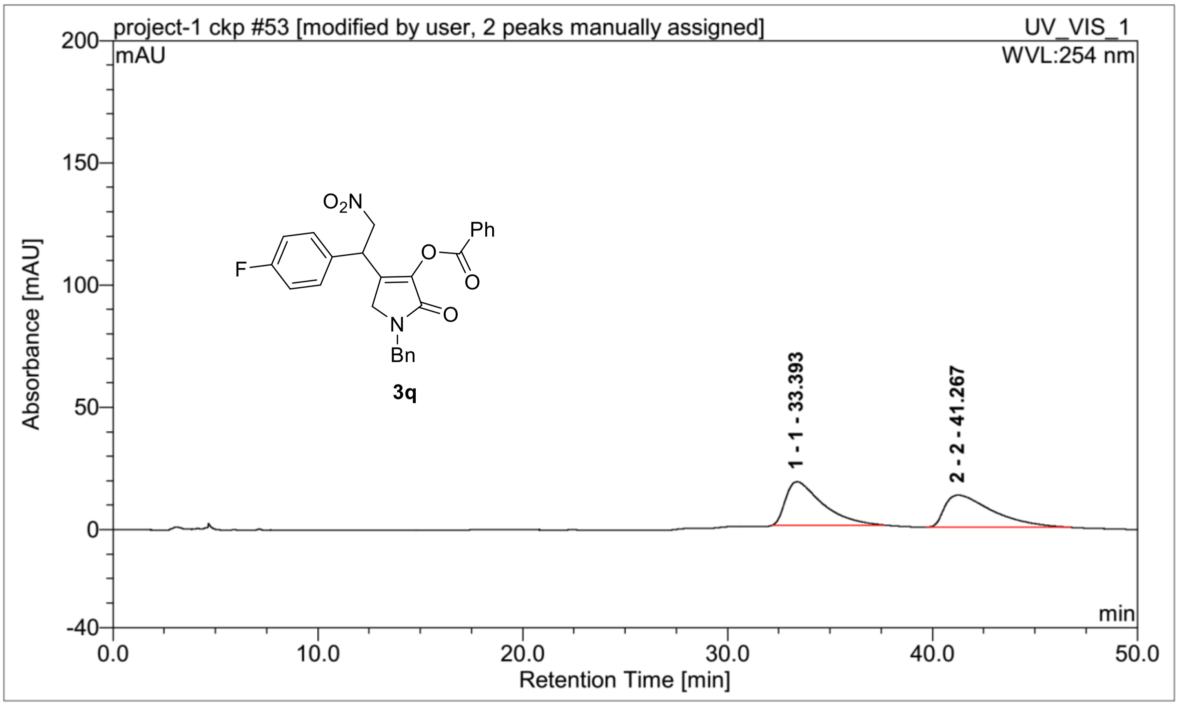

No.	Peak Name	Ret.Time (detected) min	Area mAU*min	Rel.Area(ident.) %	Height mAU	Amount
1	1	33.39	37.02522	50.80098629	17.79302	n.a.
2	2	41.27	35.858	49.19901371	13.123	n.a.

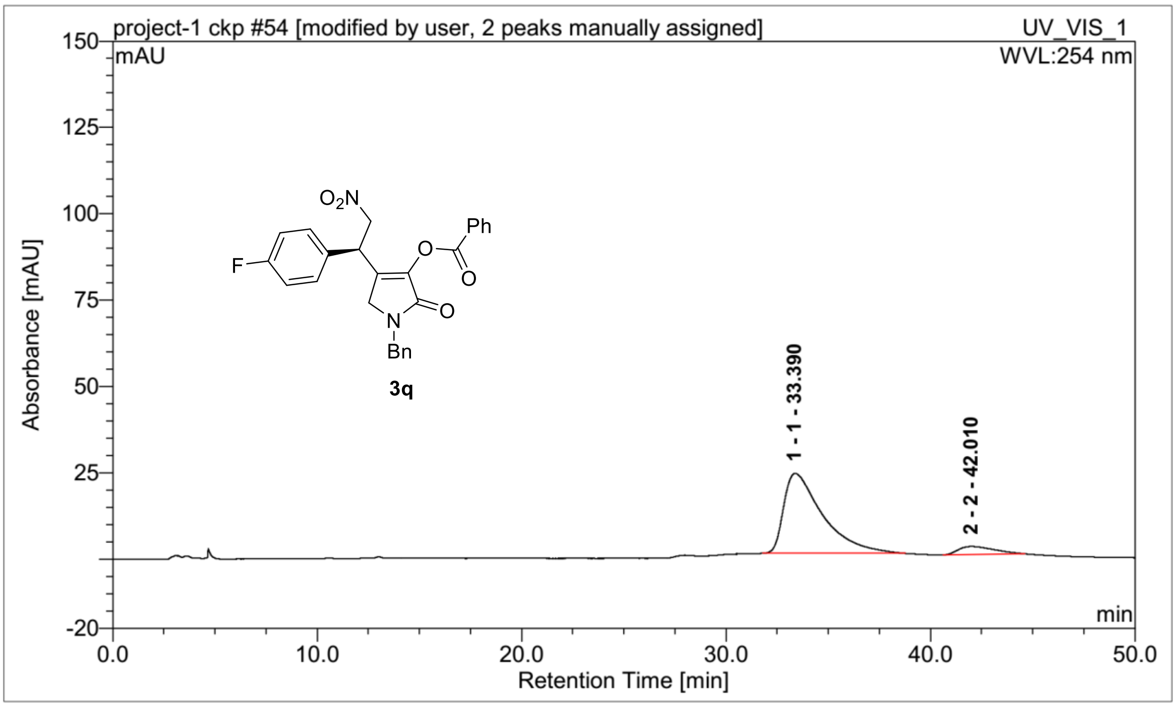

No.	Peak Name	Ret.Time (detected) min	Area mAU*min	Rel.Area(ident.) %	Height mAU	Amount
1	1	33.39	51.21042	91.49628513	23.13561	n.a.
2	2	42.01	4.760	8.503714867	2.337	n.a.

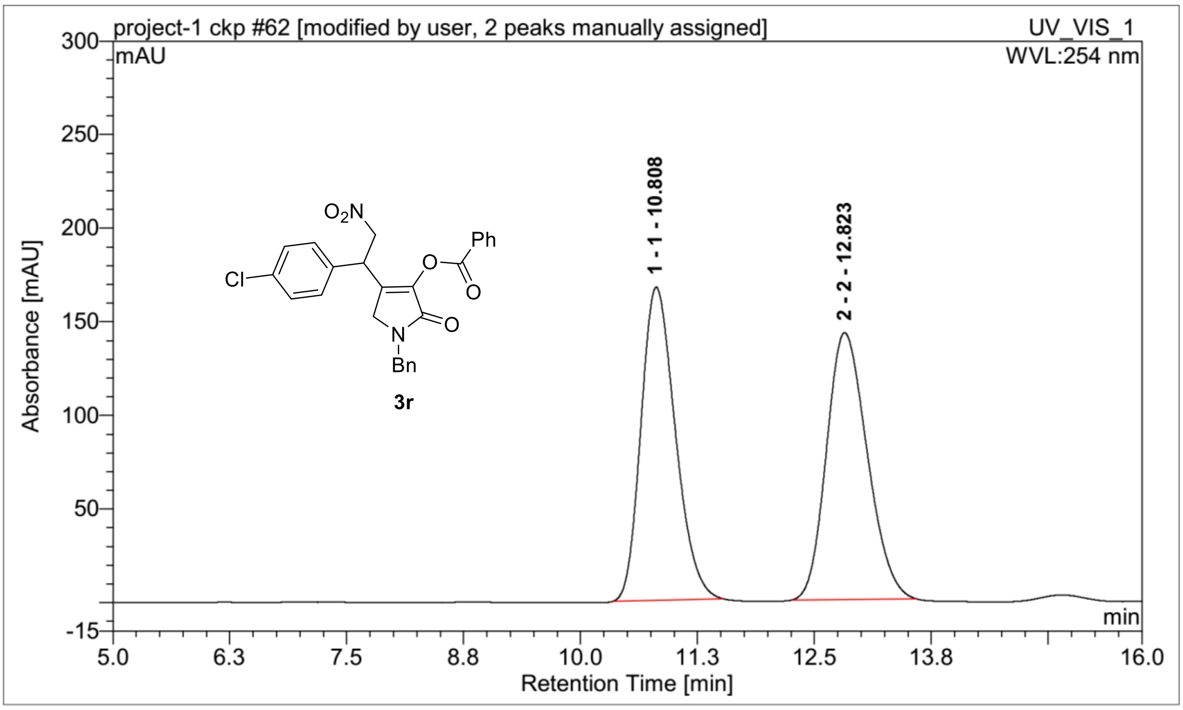

No.	Peak Name	Ret.Time (detected) min	Area mAU*min	Rel.Area(ident.) %	Height mAU	Amount
1	1	10.81	71.01245	49.72081463	167.281	n.a.
2	2	12.82	71.810	50.27918537	142.475	n.a.

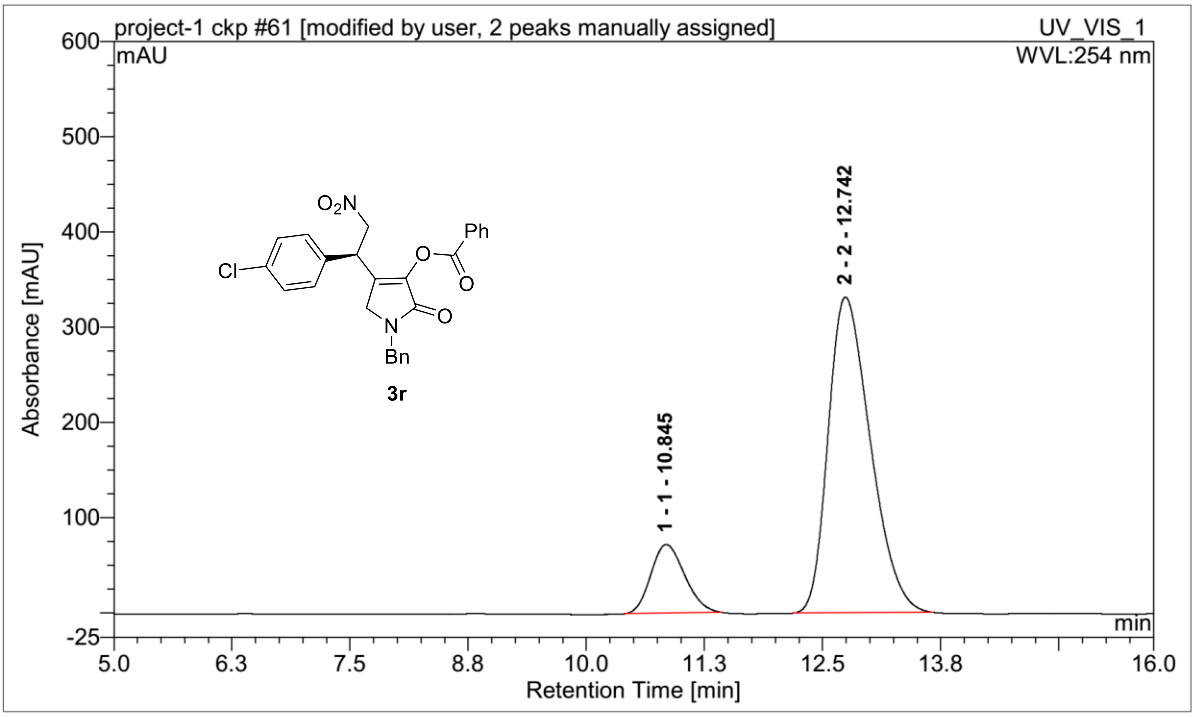

No.	Peak Name	Ret.Time (detected) min	Area mAU*min	Rel.Area(ident.) %	Height mAU	Amount
1	1	10.85	29.90189	14.84916038	72.00904	n.a.
2	2	12.74	171.469	85.15083962	331.303	n.a.

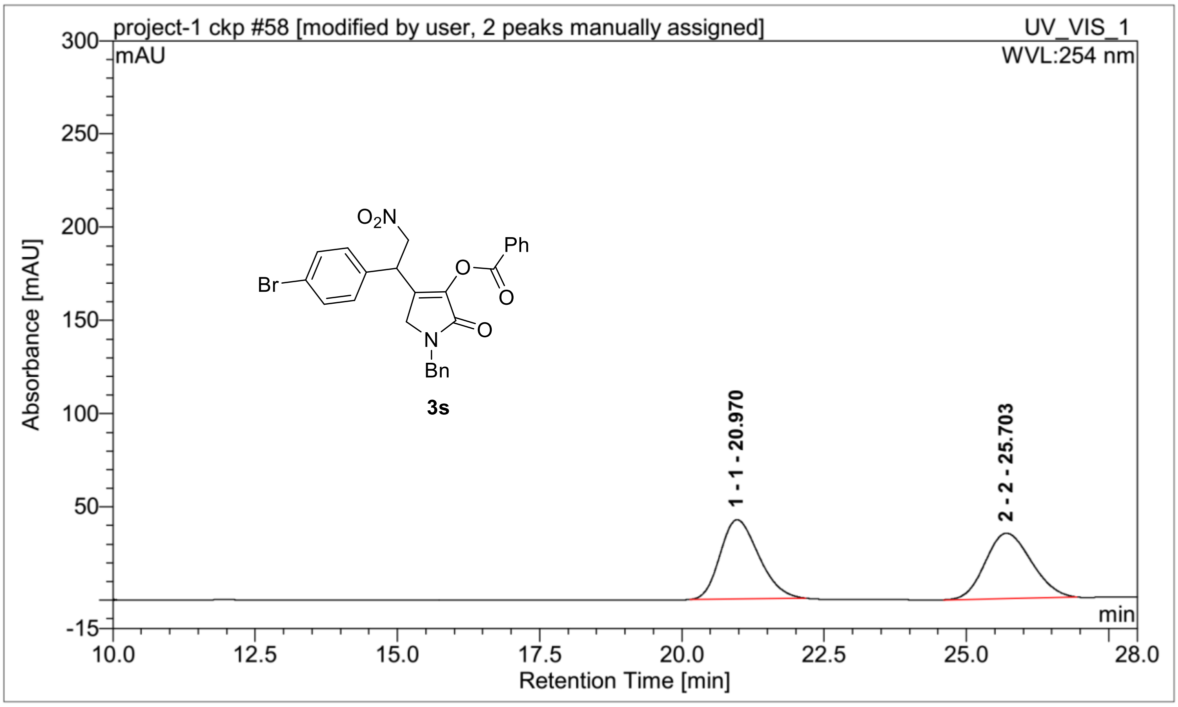

No.	Peak Name	Ret.Time (detected) min	Area mAU*min	Rel.Area(ident.) %	Height mAU	Amount
1	1	20.97	33.38294	50.47388693	42.41479	n.a.
2	2	25.70	32.756	49.52611307	34.960	n.a.

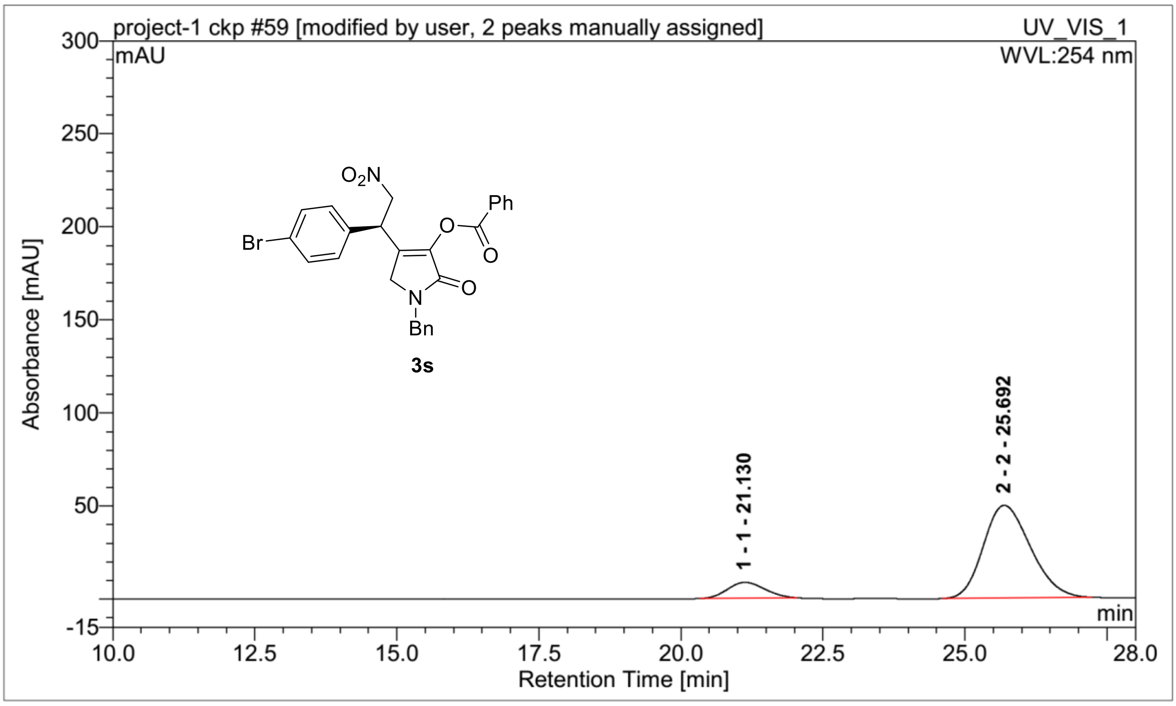

No.	Peak Name	Ret.Time (detected) min	Area mAU*min	Rel.Area(ident.) %	Height mAU	Amount
1	1	21.13	6.446094	11.86378467	8.49178	n.a.
2	2	25.69	47.888	88.13621533	49.821	n.a.

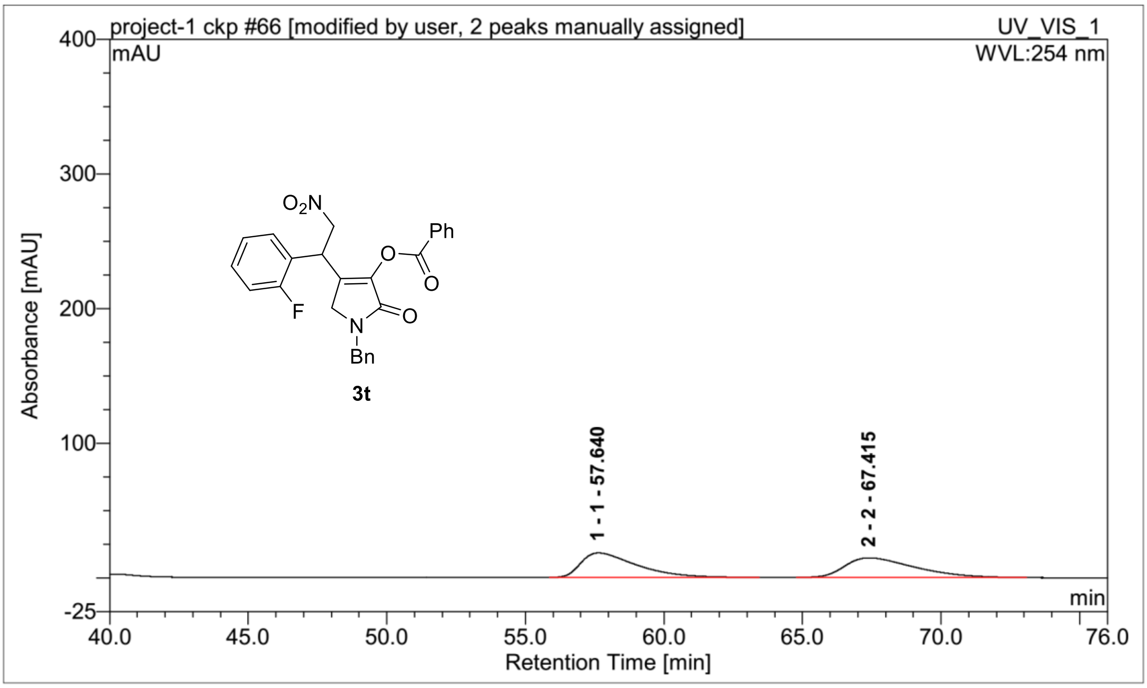

No.	Peak Name	Ret.Time (detected) min	Area mAU*min	Rel.Area(ident.) %	Height mAU	Amount
1	1	57.64	42.78868	50.11148574	18.26297	n.a.
2	2	67.42	42.598	49.88851426	14.589	n.a.

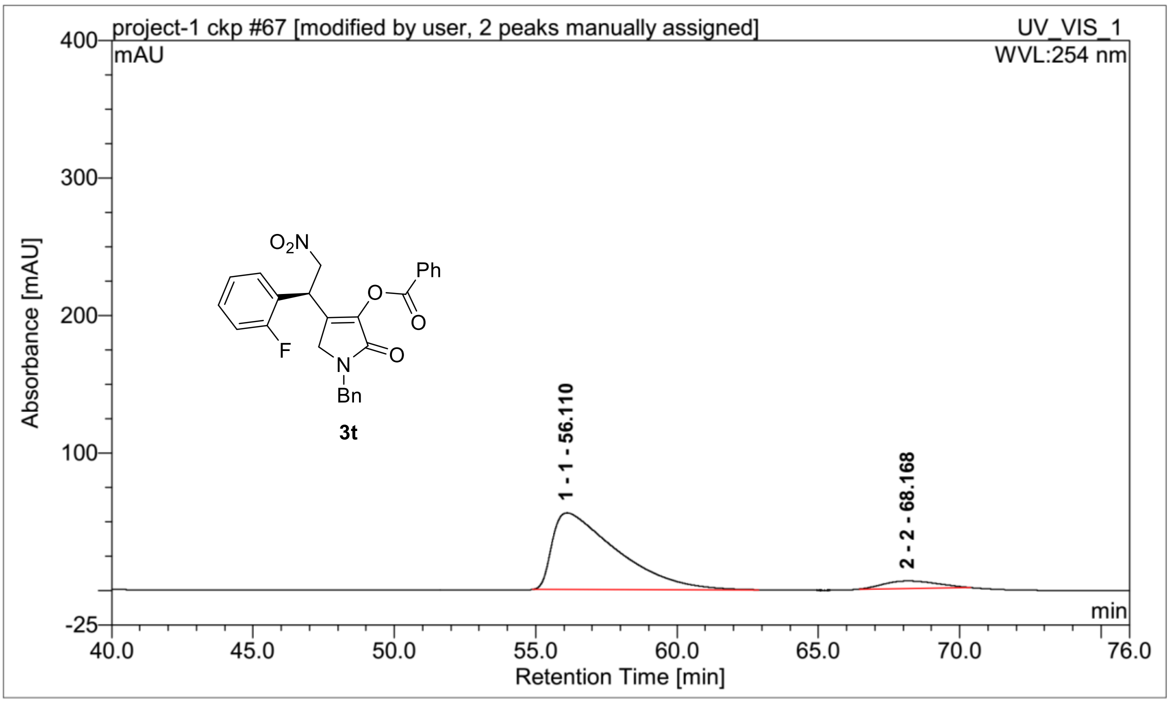

No.	Peak Name	Ret.Time (detected) min	Area mAU*min	Rel.Area(ident.) %	Height mAU	Amount
1	1	56.11	147.7052	92.5039058	55.76729	n.a.
2	2	68.17	11.969	7.496094196	5.467	n.a.

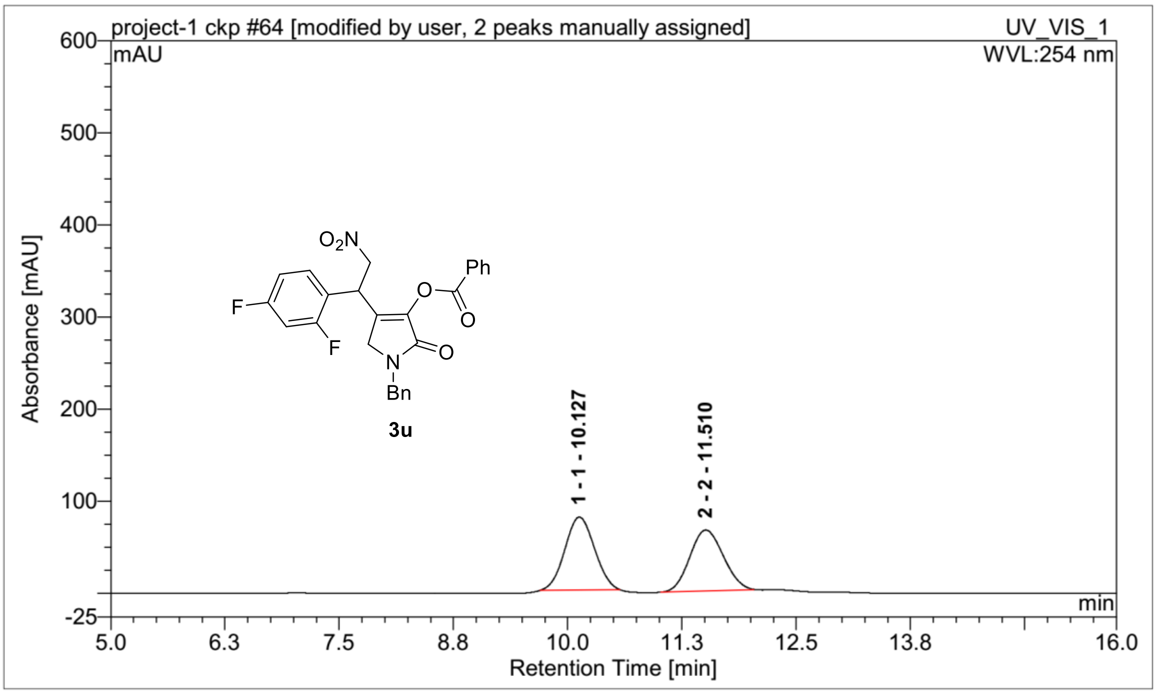

No.	Peak Name	Ret.Time (detected) min	Area mAU*min	Rel.Area(ident.) %	Height mAU	Amount
1	1	10.13	29.83827	51.55143988	79.08016	n.a.
2	2	11.51	28.042	48.44856012	66.056	n.a.

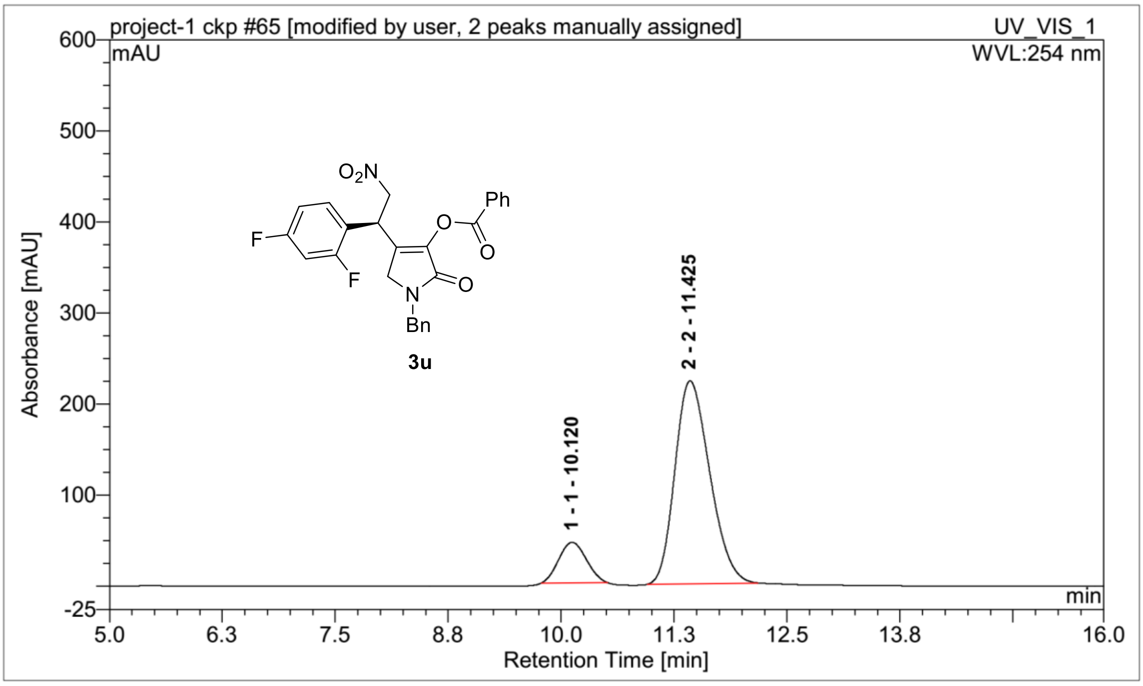

No.	Peak Name	Ret.Time (detected) min	Area mAU*min	Rel.Area(ident.) %	Height mAU	Amount
1	1	10.12	15.70124	13.6428715	44.45621	n.a.
2	2	11.43	99.386	86.3571285	222.589	n.a.

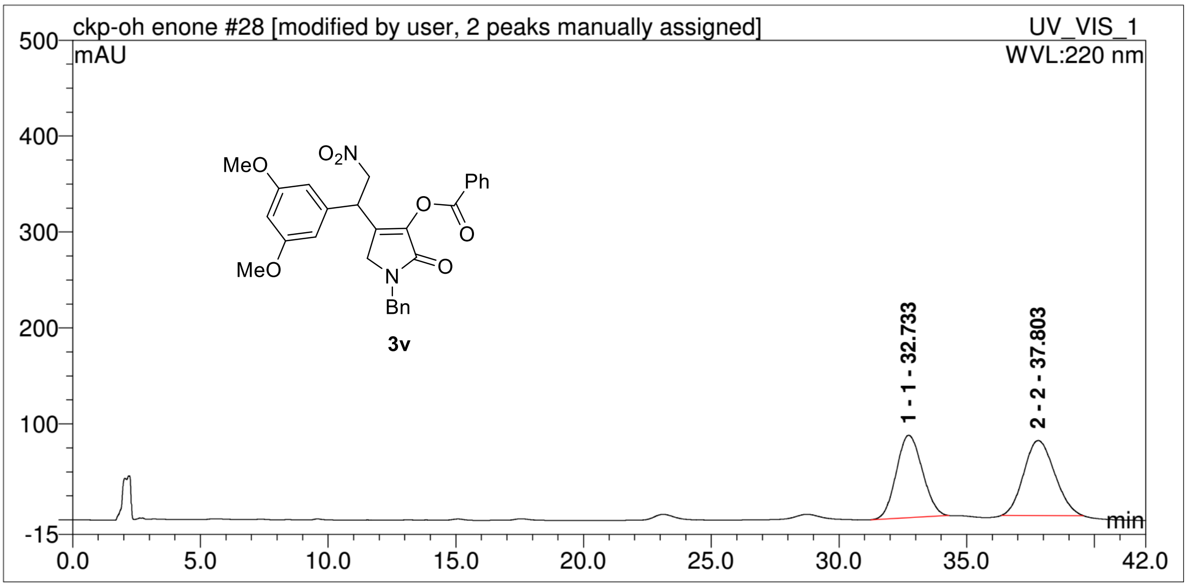

No.	Peak Name	Ret.Time (detected) min	Area mAU*min	Rel.Area(ident.) %	Height mAU	Amount
1	1	32.73	101.7329	48.11551612	85.81185	n.a.
2	2	37.80	109.702	51.88448388	78.151	n.a.

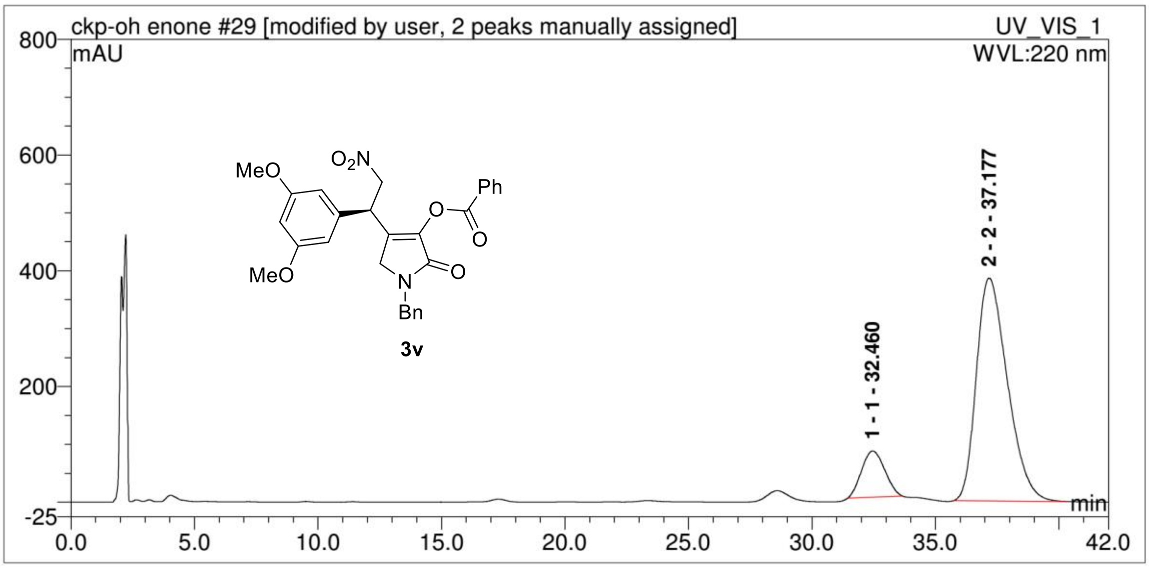

No.	Peak Name	Ret.Time (detected) min	Area mAU*min	Rel.Area(ident.) %	Height mAU	Amount
1	1	32.46	86.86257	13.3890642	79.82599	n.a.
2	2	37.18	561.895	86.6109358	385.027	n.a.

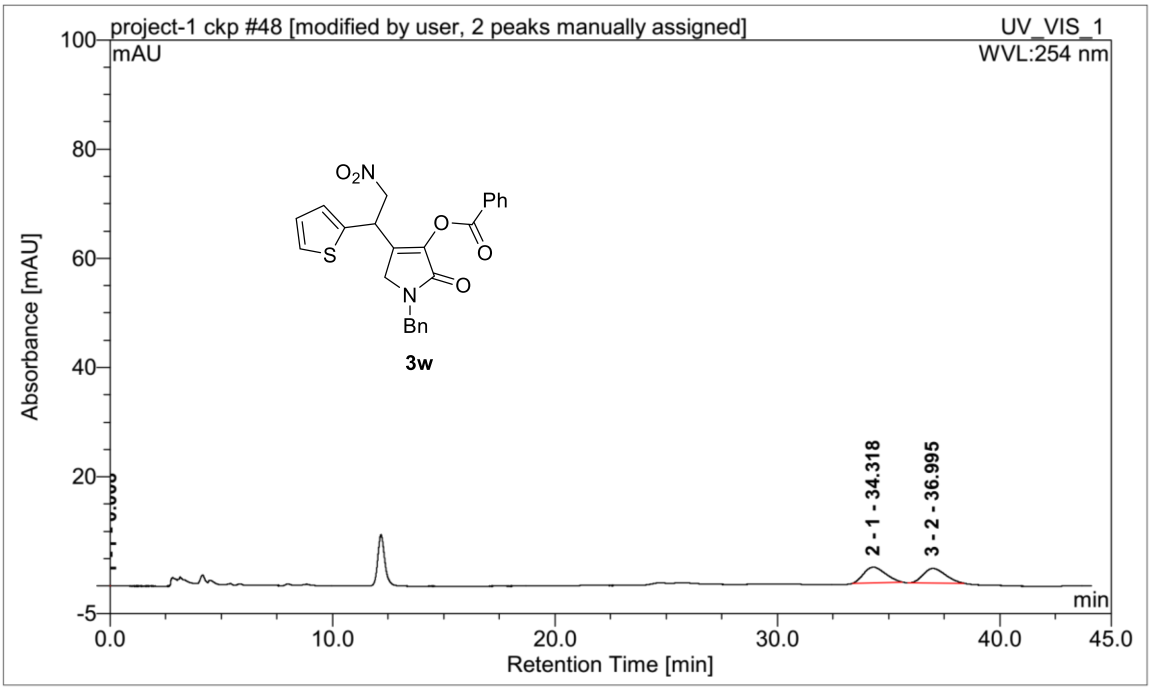

No.	Peak Name	Ret.Time (detected) min	Area mAU*min	Rel.Area(ident.) %	Height mAU	Amount
2	1	34.32	3.167195	50.68666617	2.82844	n.a.
3	2	37.00	3.081	49.31293107	2.667	n.a.

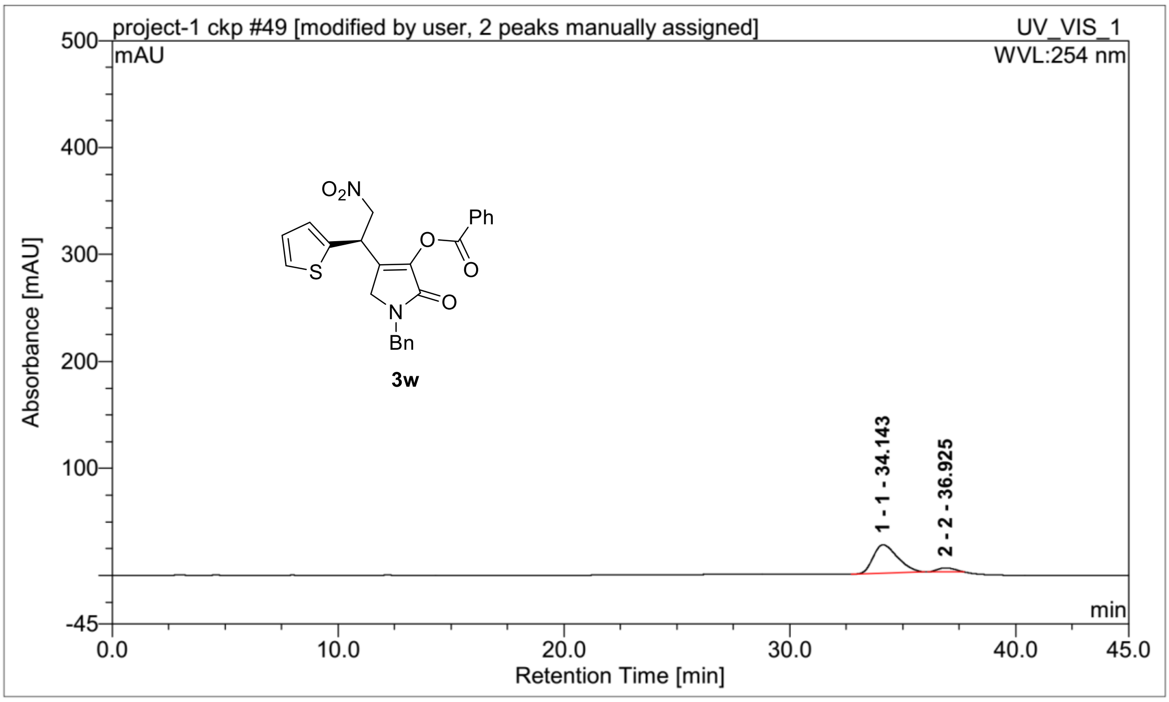

No.	Peak Name	Ret.Time (detected) min	Area mAU*min	Rel.Area(ident.) %	Height mAU	Amount
1	1	34.14	33.43863	90.59762485	26.51936	n.a.
2	2	36.93	3.470	9.402375147	3.852	n.a.

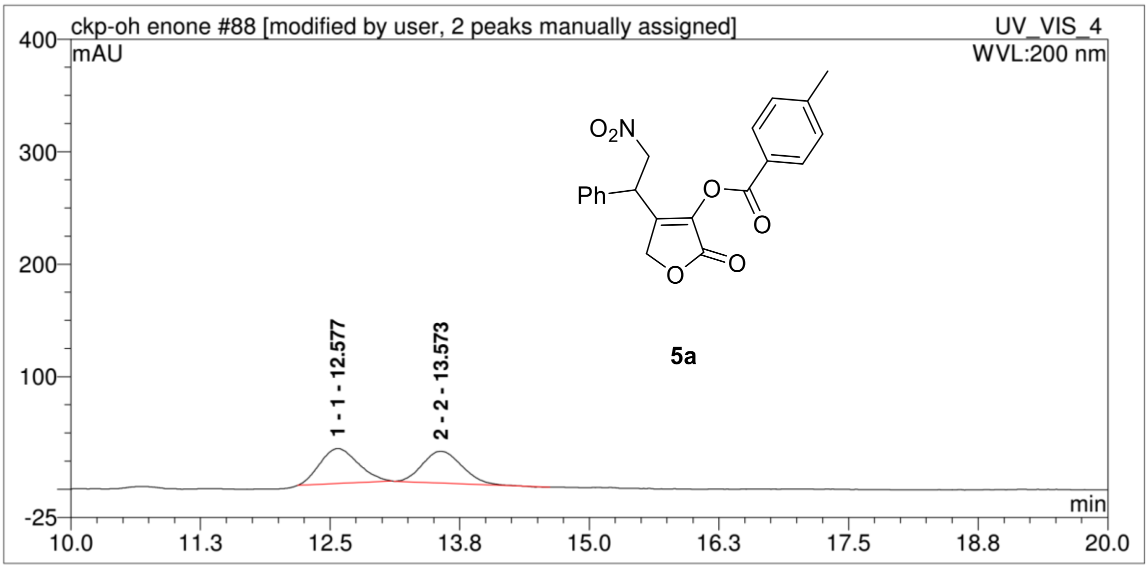

No.	Peak Name	Ret.Time (detected) min	Area mAU*min	Rel.Area(ident.) %	Height mAU	Amount
1	1	12.58	13.26235	51.51301704	31.1916	n.a.
2	2	13.57	12.483	48.48698296	28.325	n.a.

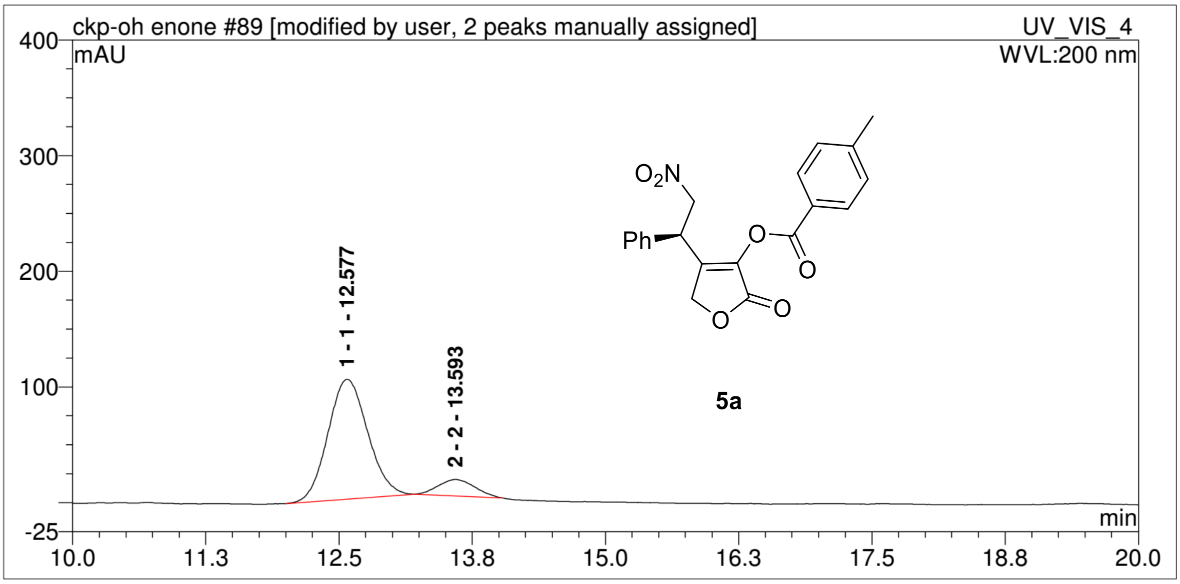

No.	Peak Name	Ret.Time (detected) min	Area mAU*min	Rel.Area(ident.) %	Height mAU	Amount
1	1	12.58	44.85525	88.81662632	103.6058	n.a.
2	2	13.59	5.648	11.18337368	14.185	n.a.

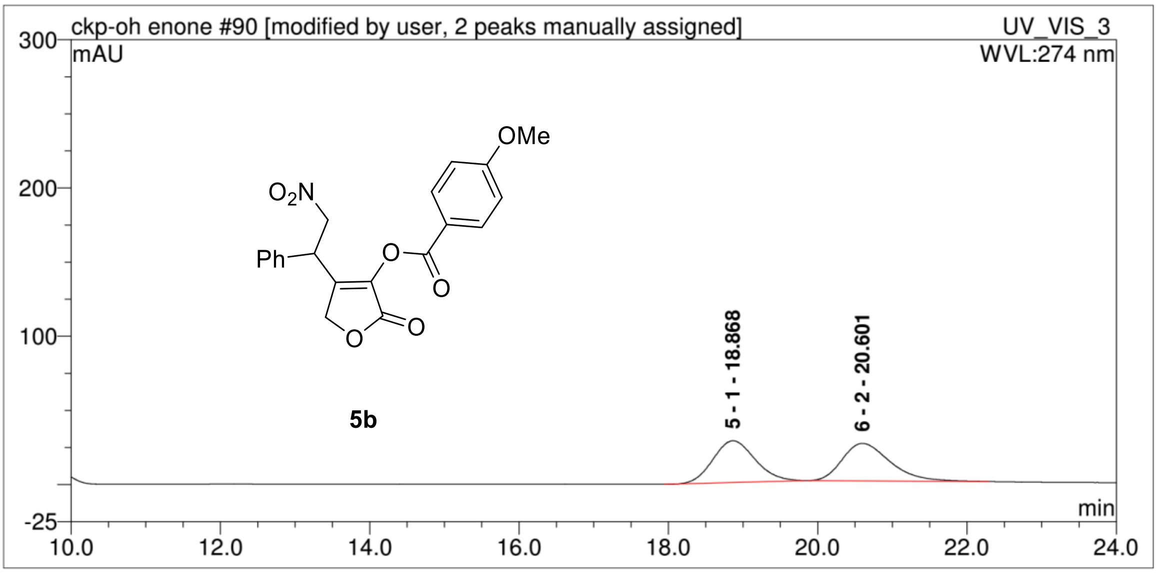

No.	Peak Name	Ret.Time (detected) min	Area mAU*min	Rel.Area(ident.) %	Height mAU	Amount
5	1	18.87	18.42876	49.46542198	28.12987	n.a.
6	2	20.60	18.827	50.53457802	25.268	n.a.

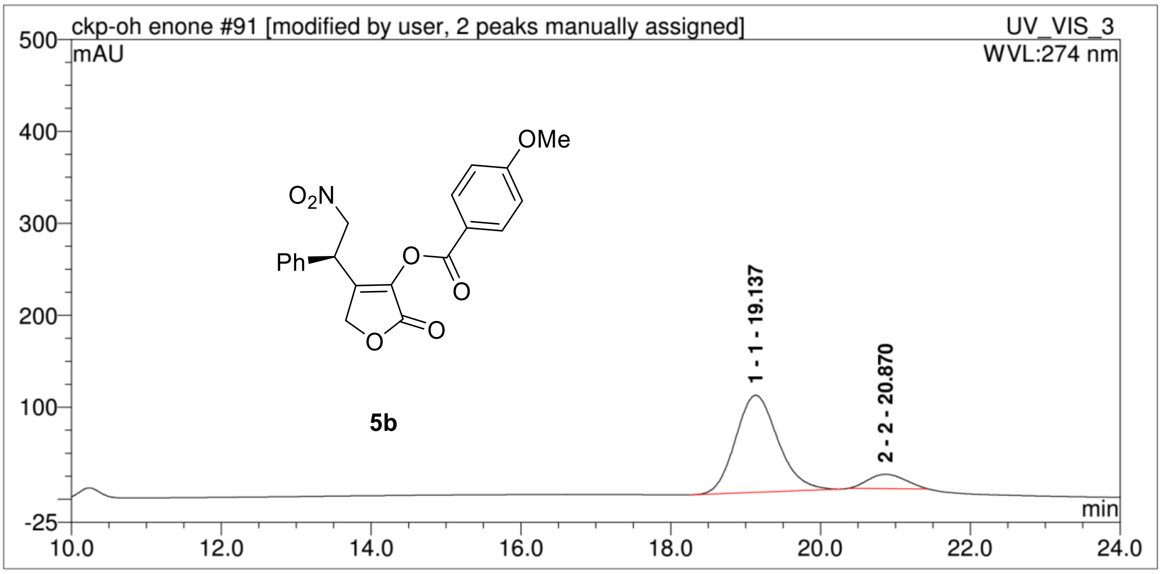

No.	Peak Name	Ret.Time (detected) min	Area mAU*min	Rel.Area(ident.) %	Height mAU	Amount
1	1	19.14	69.07896	88.55887293	105.4466	n.a.
2	2	20.87	8.924	11.44112707	15.558	n.a.

8. References

1. Fofana, M.; Dudognon, Y.; Bertrand, L.; Constantieux, T.; Rodriguez, J.; Ndiaye, I.; Bonne, D.; Bugaut, X. *Eur. J. Org. Chem*. **2020**, 3486–3490.
2. Maity, R.; Gharui. C.; Sil. A. K.; Pan. S. C. *Org. Lett*. **2017**, *19*, 662–665
